# Supplementary material for: Genome-wide association study identifies multiple risk loci for renal cell carcinoma
Source: Nat Commun. 2017 Jun 9;8:15724. doi: 10.1038/ncomms15724 (PMC5472706; doi:10.1038/ncomms15724)
Supplement: Supplementary Information — Supplementary Figures, Supplementary Tables, Supplementary Notes and Supplementary References [file ncomms15724-s1.pdf]

Supplementary Figures

Supplementary Figure 1. Quantile-quantile plots for the stage 1 meta-analysis.

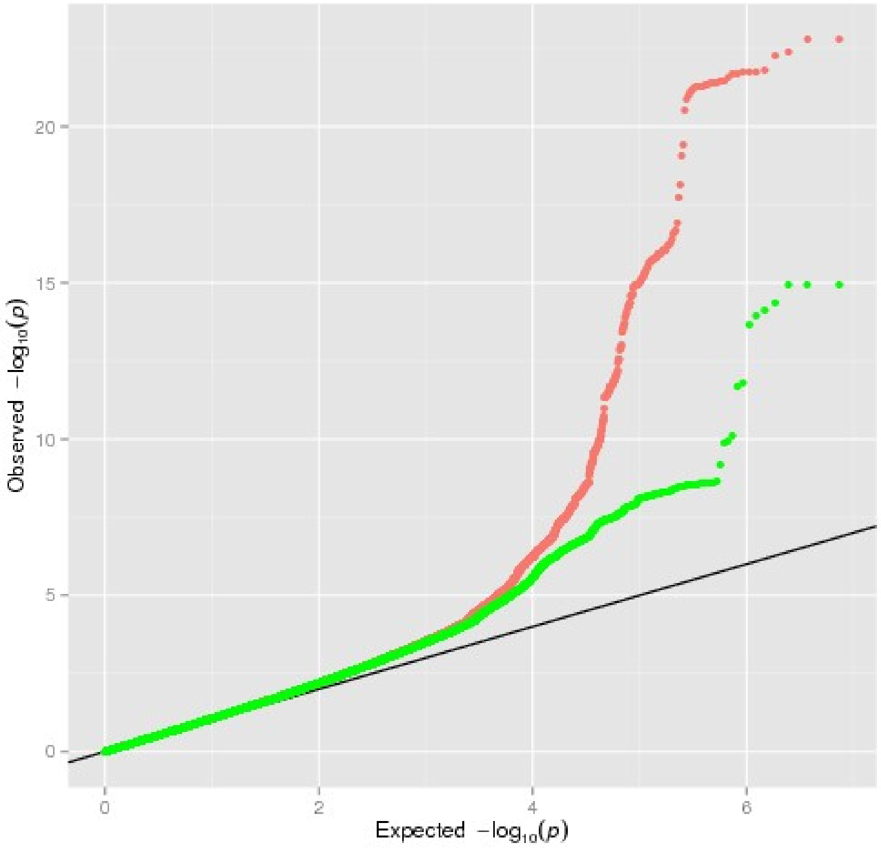

QQ plot after filtering out SNPs with MAF < 0.01. Red line includes all SNPs; green line excludes SNPs +/- 500kb of previously identified locus.

Supplementary Figure 2. Manhattan plot of GWAS association P-values.

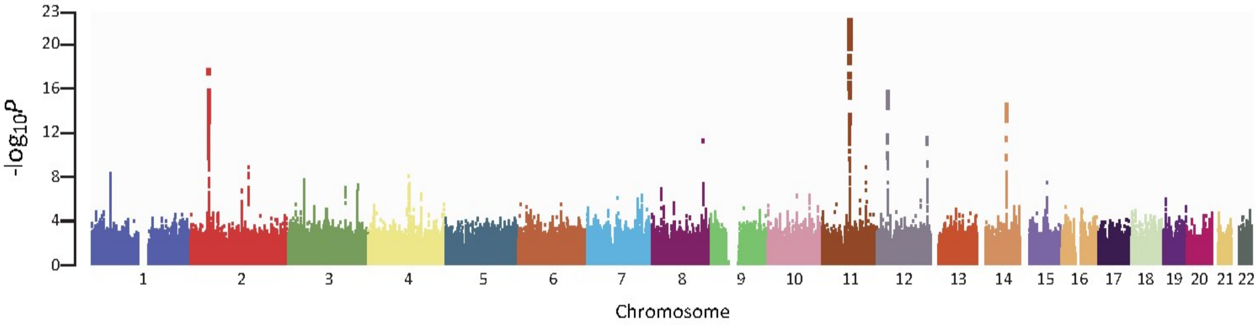

Supplementary Figure 3. Regional association LD plots

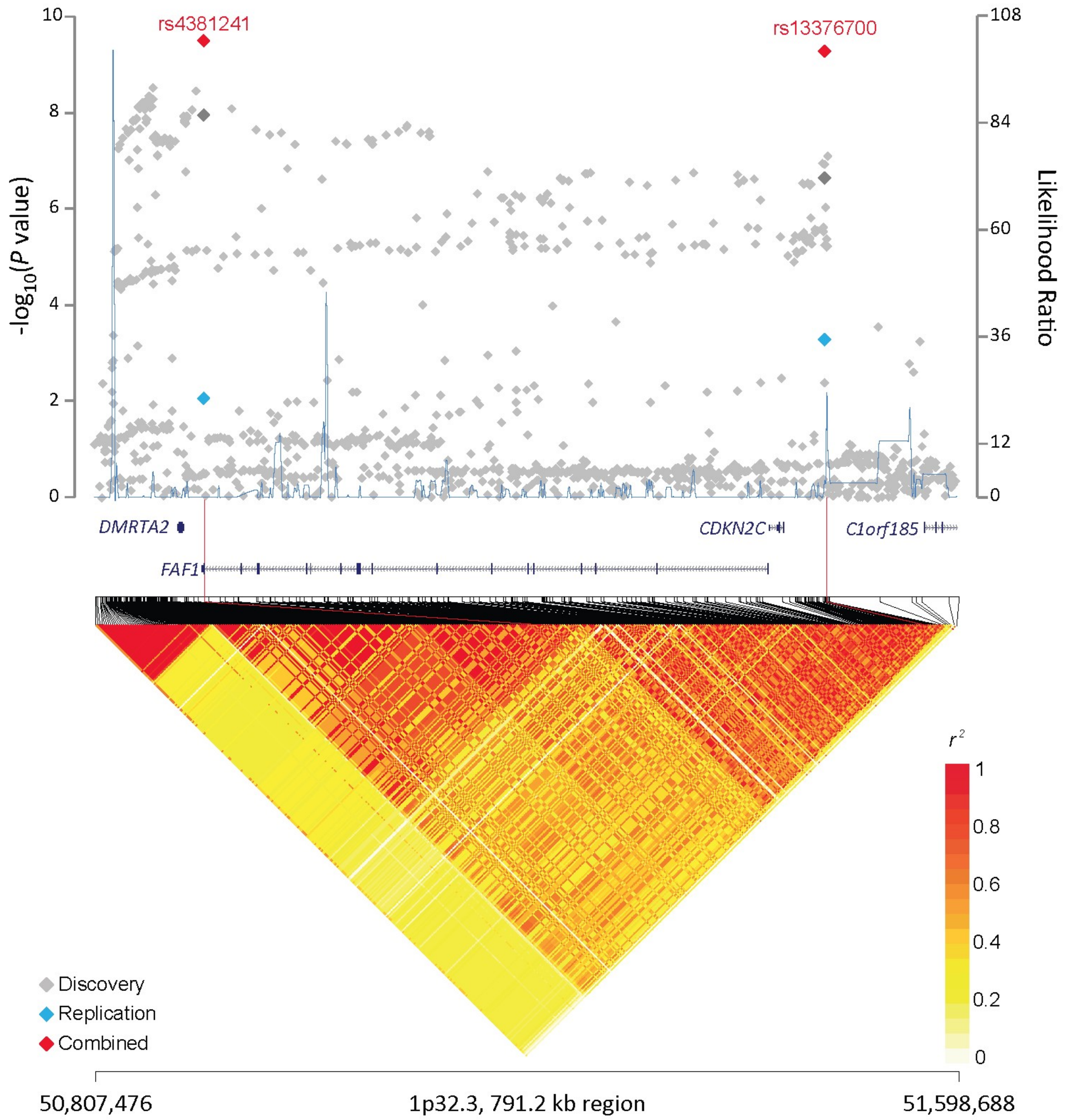

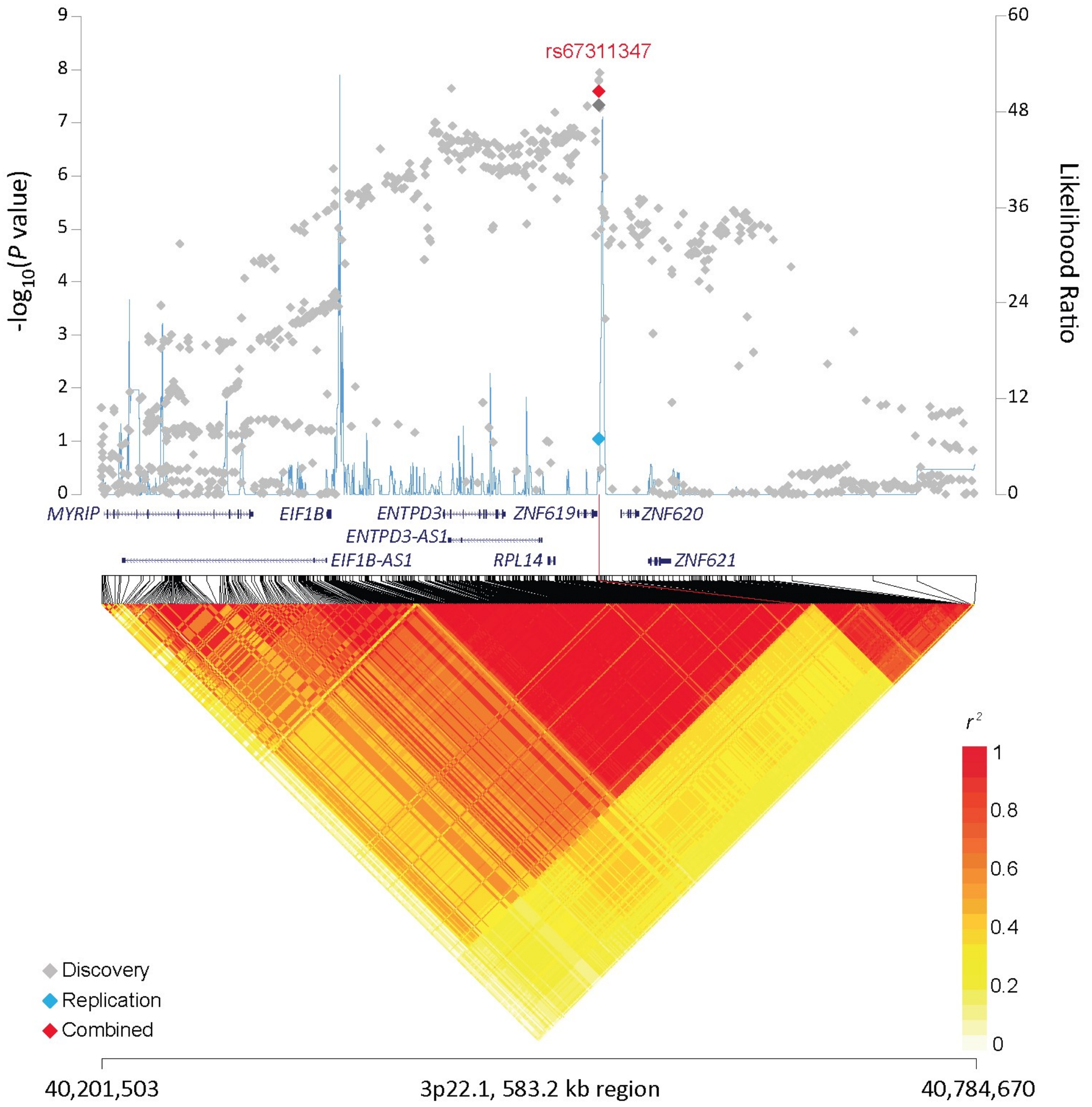

rs67311347
-log10(P value)
Likelihood Ratio
MYRIP
EIF1B
ENTPD3
ZNF619
ZNF620
ENTPD3-AS1
EIF1B-AS1
RPL14
ZNF621
r2
Discovery
Replication
Combined
40,201,503
3p22.1, 583.2 kb region
40,784,670

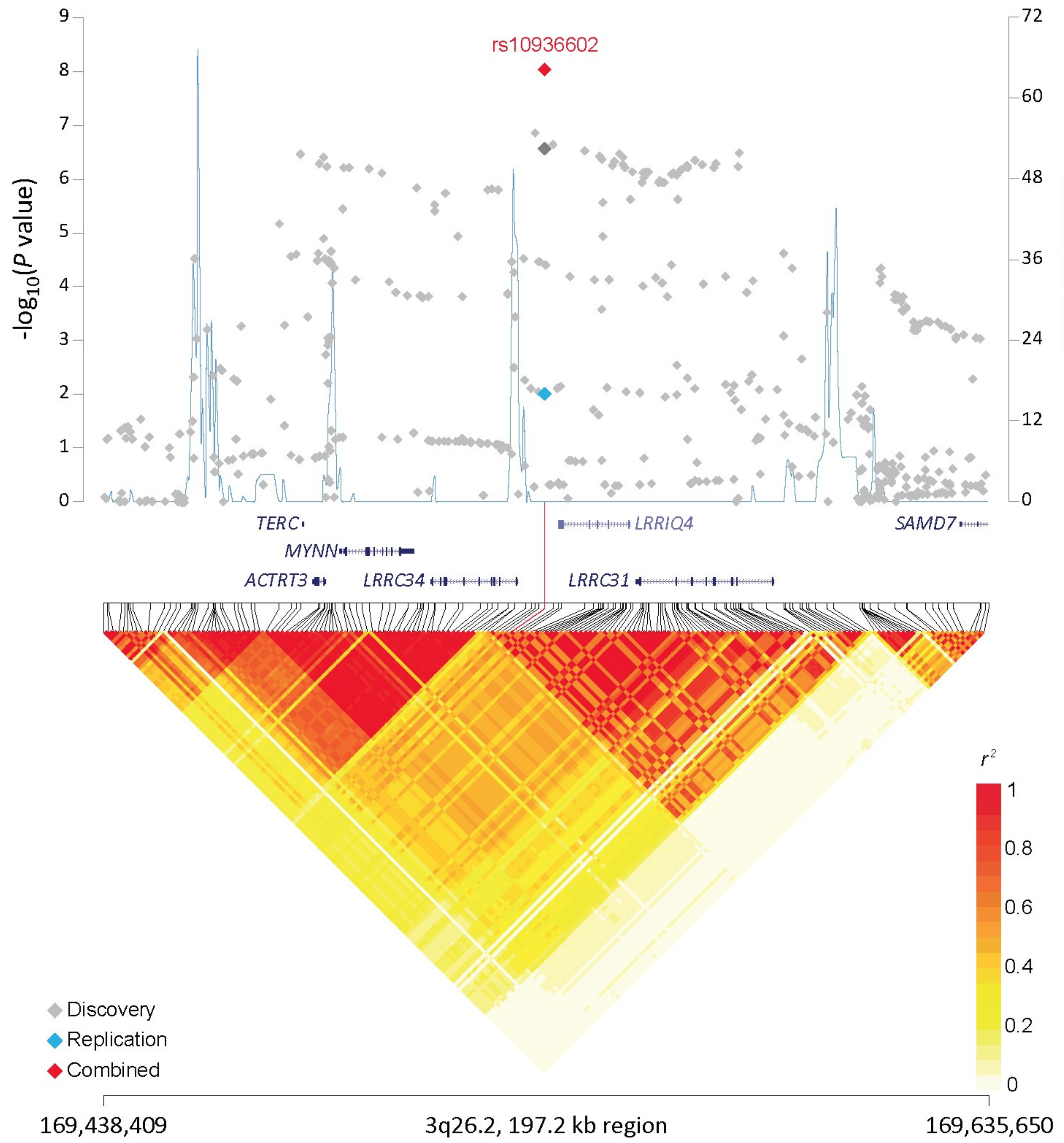

rs10936602
-log10(P value)
Likelihood Ratio
TERC
MYNN
ACTRT3
LRRC34
LRRIQ4
LRRC31
SAMD7
r2
Discovery
Replication
Combined
169,438,409
3q26.2, 197.2 kb region
169,635,650

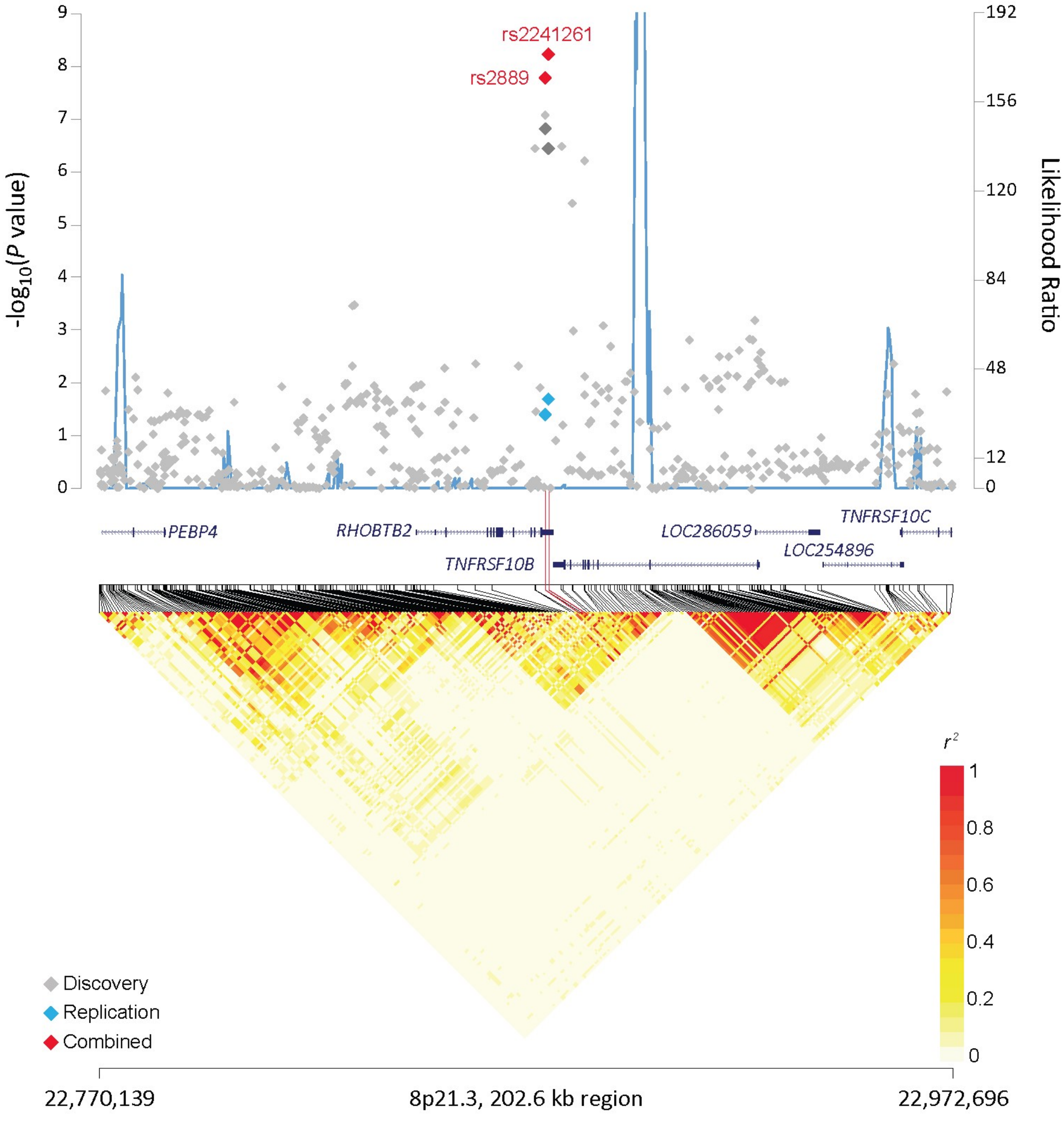

-log10(P value)
Likelihood Ratio
rs2241261
rs2889
PEBP4
RHOBTB2
TNFRSF10B
LOC286059
TNFRSF10C
LOC254896
r2
Discovery
Replication
Combined
22,770,139
8p21.3, 202.6 kb region
22,972,696

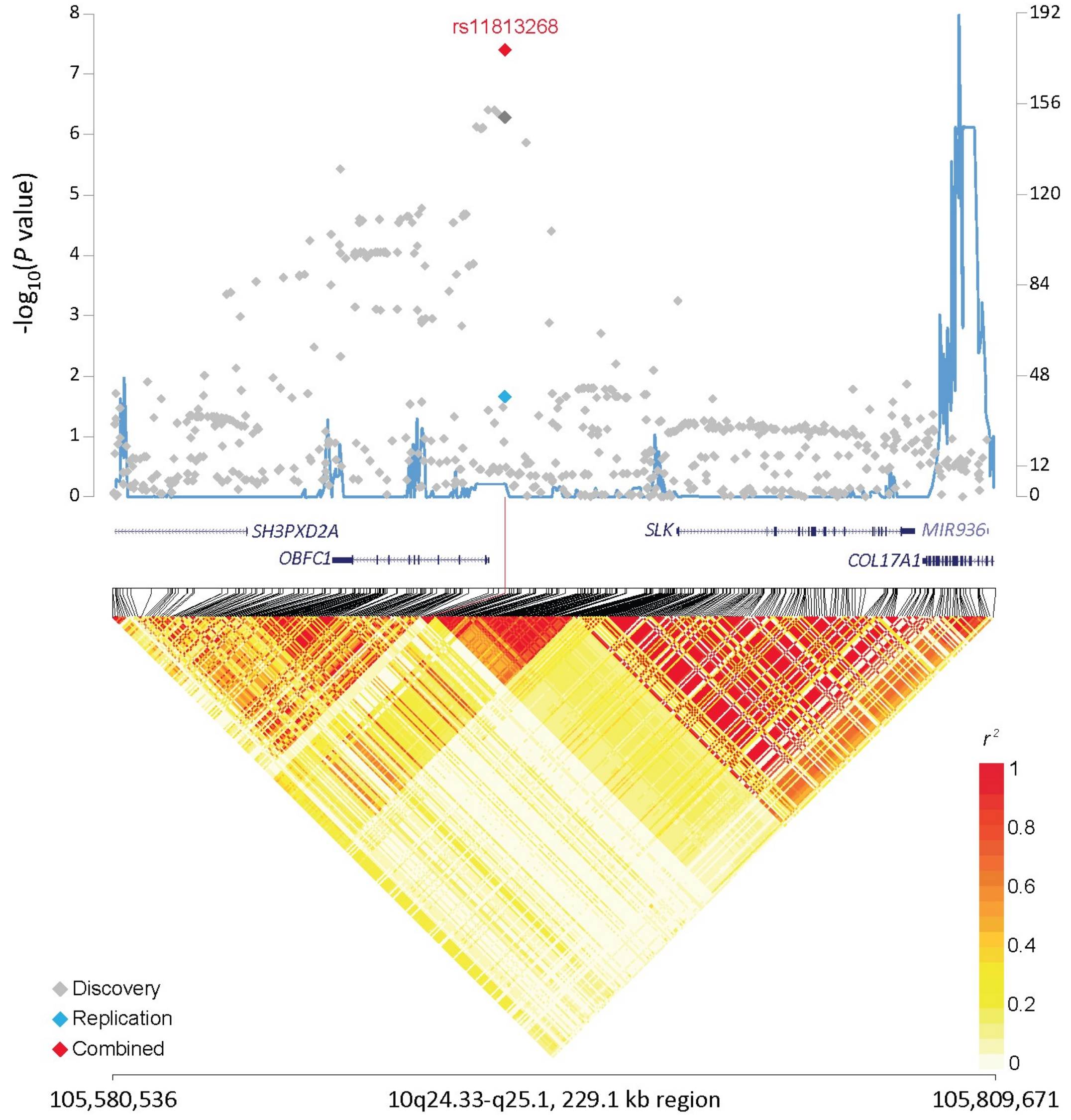

rs11813268
-log10(P value)
Likelihood Ratio
0
1
2
3
4
5
6
7
8
0
12
48
84
120
156
192
SH3PXD2A
OBFC1
SLK
MIR936
COL17A1
r2
1
0.8
0.6
0.4
0.2
0
Discovery
Replication
Combined
105,580,536
10q24.33-q25.1, 229.1 kb region
105,809,671

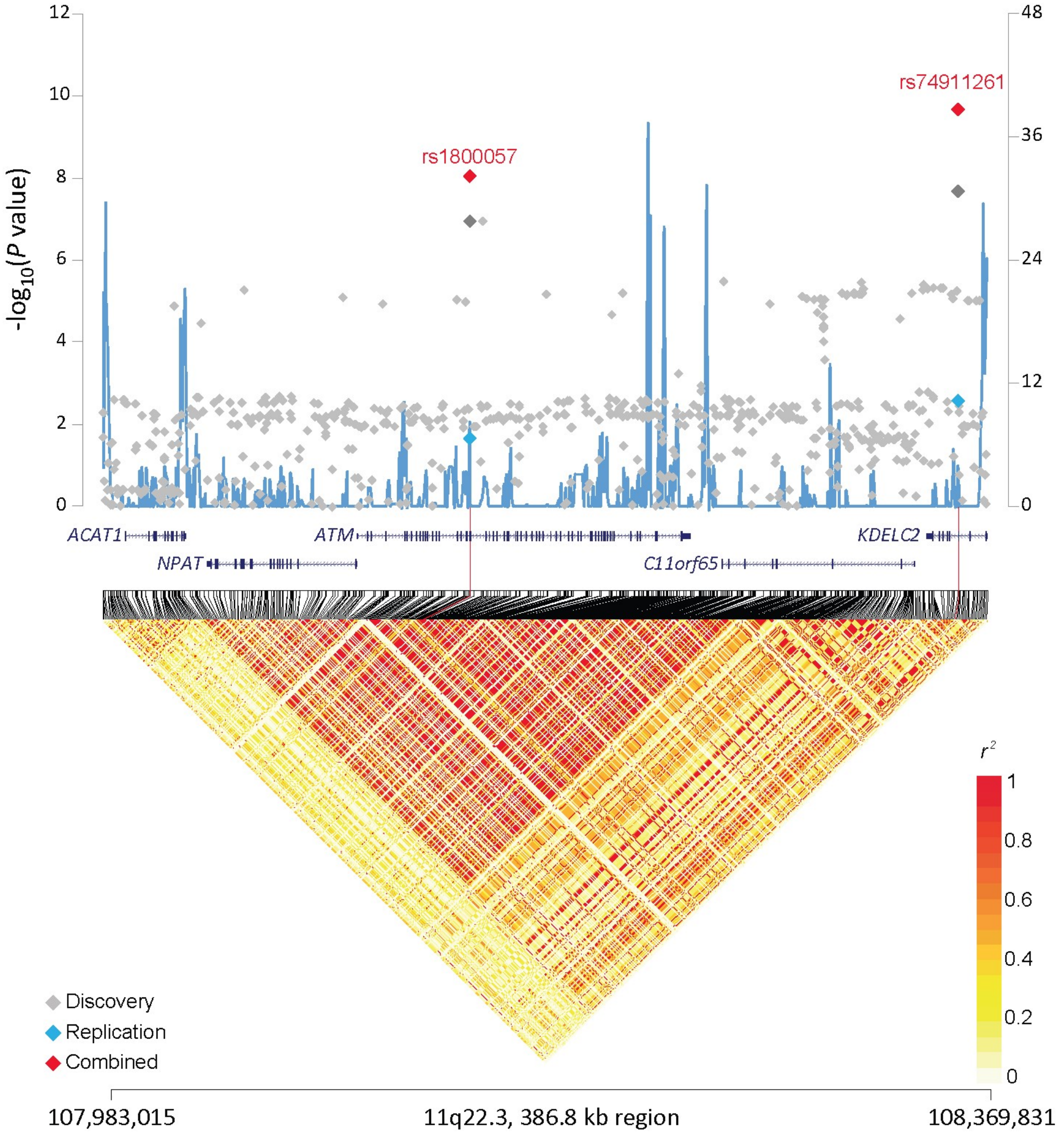
-log10(P value)
Likelihood Ratio
rs1800057
rs74911261
ACAT1
NPAT
ATM
C11orf65
KDELC2
r2
Discovery
Replication
Combined
107,983,015
11q22.3, 386.8 kb region
108,369,831

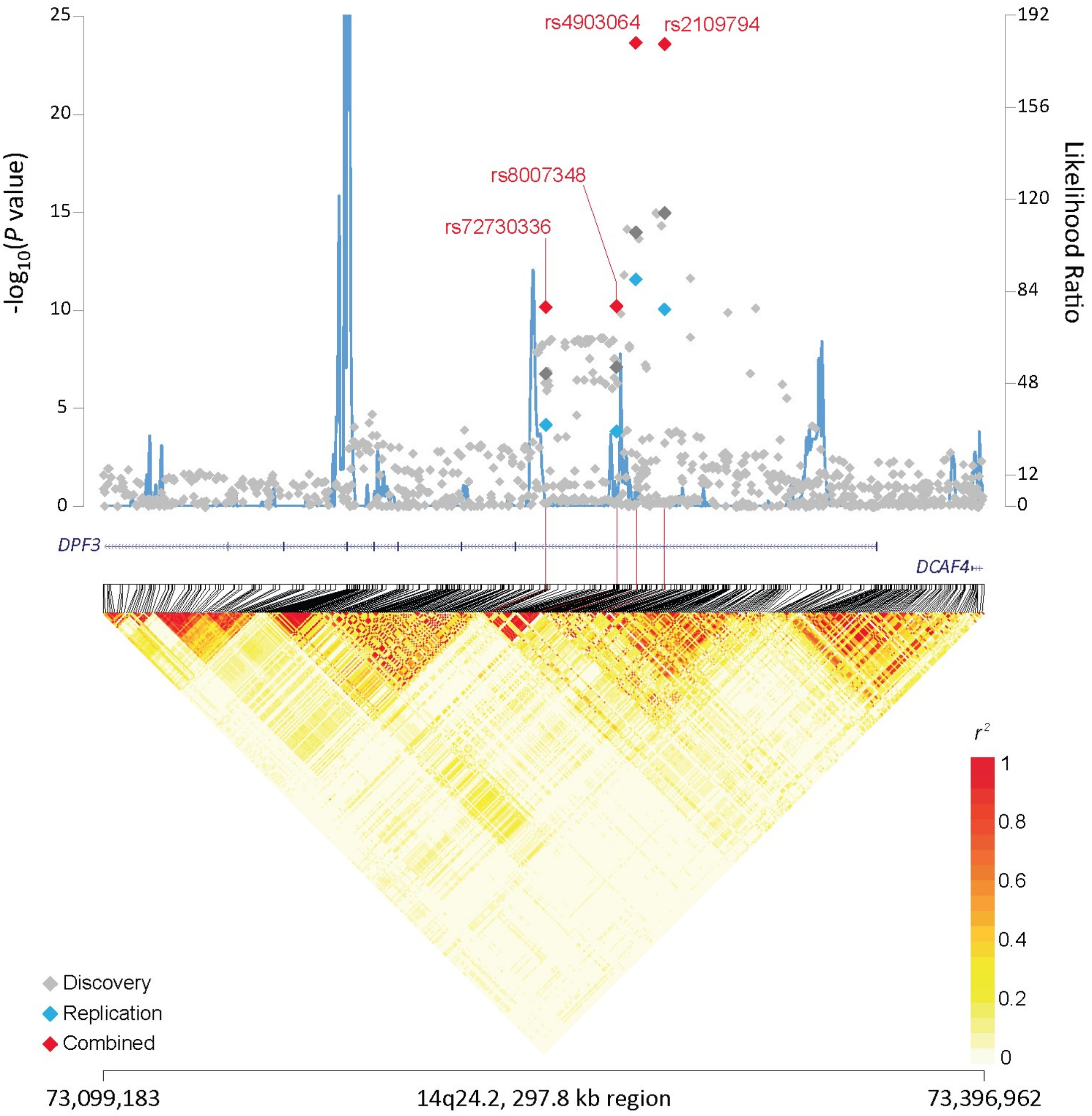

25
20
15
10
5
0
-log10(P value)
192
156
120
84
48
12
0
Likelihood Ratio
rs4903064
rs2109794
rs8007348
rs72730336
DPF3
DCAF4
r2
1
0.8
0.6
0.4
0.2
0
Discovery
Replication
Combined
73,099,183
14q24.2, 297.8 kb region
73,396,962

Supplementary Figure 4. SNP analyses stratified by sex, body mass index (BMI; <25, 25-29, 30+ kg/m2), smoking status (never, former, current), and history of diagnosed hypertension

		Heterogeneity P-value				
**RSID**	**Position**	**BMI**	**Hypertension**	**Sex**	**Smoking**	**Notes**
rs4381241	chr1:50907438	0.1260	0.2971	0.9800	0.4365	
rs13376700	chr1:51477643	0.1441	0.8296	0.5867	0.7499	
rs6706003	chr2:46563392	0.7553	0.5484	0.4456	0.9480	
rs6755594	chr2:46589295	0.0072	0.5257	0.3656	0.4870	
rs72851889	chr2:144694129	0.4763	0.0759	0.9594	0.7222	
rs72855540	chr2:144990548	0.6065	0.1736	0.3559	0.7988	
rs67311347	chr3:40533243	0.9734	0.9460	0.2067	0.4066	
rs2203002	chr3:141796239	0.6793	0.3010	0.5532	0.8355	
rs10936602	chr3:169536637	0.0577	0.6883	0.0866	0.2242	
rs234043	chr3:172313367	0.4390	0.3124	0.9282	0.2813	
rs7697932	chr4:101005318	0.7494	0.0033	0.4040	0.0074	
rs17050872	chr4:131097748	0.5047	0.8624	0.4159	0.5546	
rs76912165	chr5:92965344	0.0322	0.8506	0.1022	0.5913	
rs1266819	chr6:52094946	0.8495	0.4585	0.0836	0.8467	
rs59294613	chr7:124554267	0.8329	0.3840	0.9974	0.0082	
rs73149977	chr7:134488864	0.8895	0.4190	0.6981	0.0130	
rs2889	chr8:22875909	0.2791	0.5079	0.2006	0.4373	
rs2241261	chr8:22876739	0.1427	0.4912	0.5391	0.6539	
rs7913447	chr10:73992040	0.7282	0.8890	0.8155	0.1781	
rs11813268	chr10:105682296	0.8877	0.6983	0.1433	0.2921	
rs77774900	chr11:95830733	0.5445	0.0733	0.8029	0.1773	
rs1800057	chr11:108143456	0.2800	0.6833	0.0614	0.8114	
rs74911261	chr11:108357137	0.2001	0.6537	0.1407	0.5375	
rs77736197	chr12:107623706	0.9695	0.4229	0.5077	0.0973	
rs72730336	chr14:73248661	0.2424	0.7131	0.0043	0.9807	Stronger effect in females; some heterogeneity observed among males
rs8007348	chr14:73272730	0.2646	0.7935	0.0026	0.9964	Stronger effect in females
rs4903064	chr14:73279420	0.2630	0.8311	**<0.0001**	0.1338	Stronger effect in females; some heterogeneity observed among females
rs2109794	chr14:73288855	0.2743	0.6162	**<0.0001**	0.2676	Stronger effect in females
rs11637556	chr15:66728951	0.2492	0.8158	0.9727	0.7230	
rs4804368	chr19:7190290	0.6837	0.1725	0.5010	0.0194	

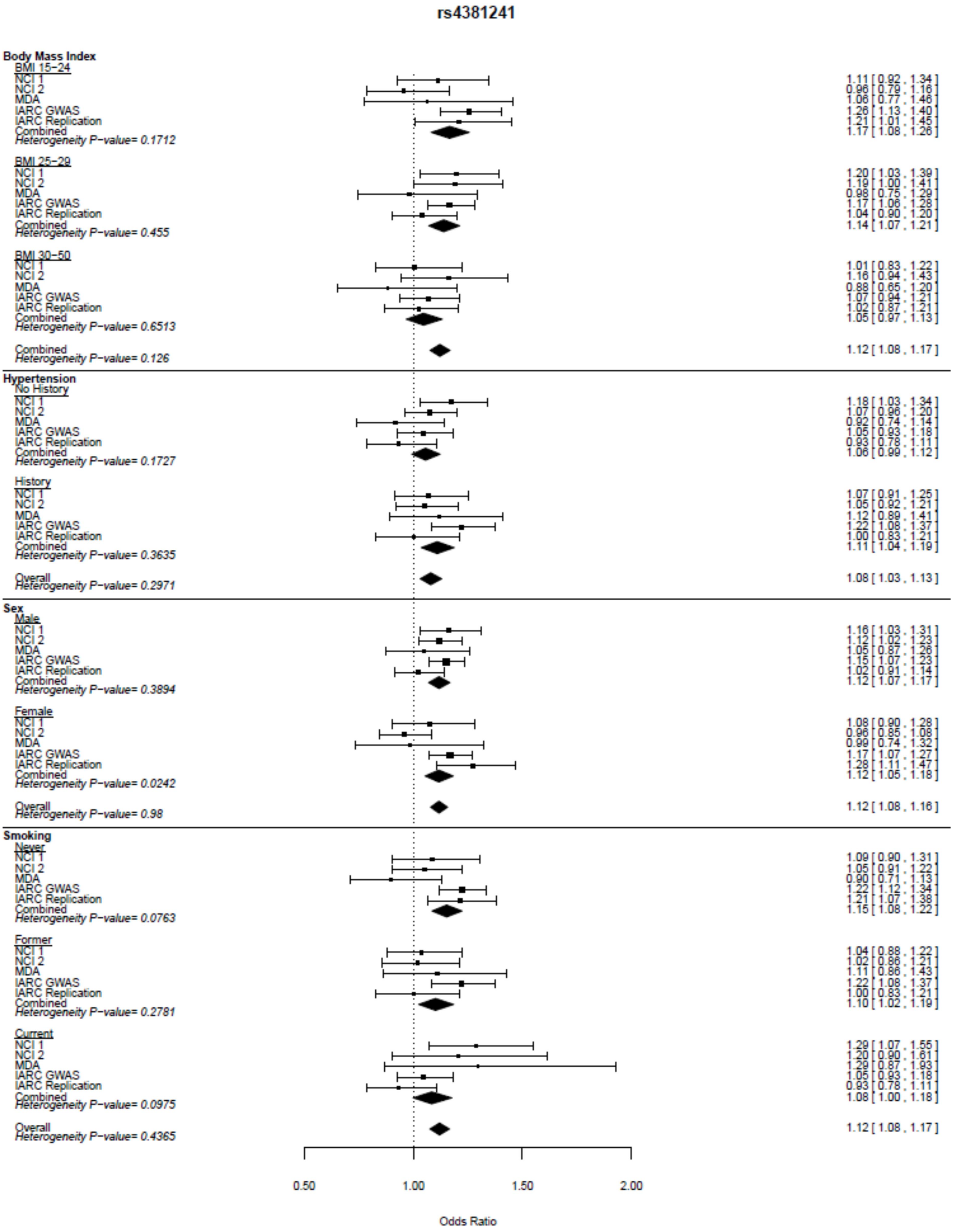
rs4381241
Body Mass Index
BMI 15−24
NCI 1
NCI 2
MDA
IARC GWAS
IARC Replication
Combined
Heterogeneity P−value= 0.1712
1.11 [0.92 , 1.34]
0.96 [0.79 , 1.16]
1.06 [0.77 , 1.46]
1.26 [1.13 , 1.40]
1.21 [1.01 , 1.45]
1.17 [1.08 , 1.26]
BMI 25−29
NCI 1
NCI 2
MDA
IARC GWAS
IARC Replication
Combined
Heterogeneity P−value= 0.455
1.20 [1.03 , 1.39]
1.19 [1.00 , 1.41]
0.98 [0.75 , 1.29]
1.17 [1.06 , 1.28]
1.04 [0.90 , 1.20]
1.14 [1.07 , 1.21]
BMI 30−50
NCI 1
NCI 2
MDA
IARC GWAS
IARC Replication
Combined
Heterogeneity P−value= 0.6513
1.01 [0.83 , 1.22]
1.16 [0.94 , 1.43]
0.88 [0.65 , 1.20]
1.07 [0.94 , 1.21]
1.02 [0.87 , 1.21]
1.05 [0.97 , 1.13]
Combined
Heterogeneity P−value= 0.126
1.12 [1.08 , 1.17]
Hypertension
No History
NCI 1
NCI 2
MDA
IARC GWAS
IARC Replication
Combined
Heterogeneity P−value= 0.1727
1.18 [1.03 , 1.34]
1.07 [0.96 , 1.20]
0.92 [0.74 , 1.14]
1.05 [0.93 , 1.18]
0.93 [0.78 , 1.11]
1.06 [0.99 , 1.12]
History
NCI 1
NCI 2
MDA
IARC GWAS
IARC Replication
Combined
Heterogeneity P−value= 0.3635
1.07 [0.91 , 1.25]
1.05 [0.92 , 1.21]
1.12 [0.89 , 1.41]
1.22 [1.08 , 1.37]
1.00 [0.83 , 1.21]
1.11 [1.04 , 1.19]
Overall
Heterogeneity P−value= 0.2971
1.08 [1.03 , 1.13]
Sex
Male
NCI 1
NCI 2
MDA
IARC GWAS
IARC Replication
Combined
Heterogeneity P−value= 0.3894
1.16 [1.03 , 1.31]
1.12 [1.02 , 1.23]
1.05 [0.87 , 1.26]
1.15 [1.07 , 1.23]
1.02 [0.91 , 1.14]
1.12 [1.07 , 1.17]
Female
NCI 1
NCI 2
MDA
IARC GWAS
IARC Replication
Combined
Heterogeneity P−value= 0.0242
1.08 [0.90 , 1.28]
0.96 [0.85 , 1.08]
0.99 [0.74 , 1.32]
1.17 [1.07 , 1.27]
1.28 [1.11 , 1.47]
1.12 [1.05 , 1.18]
Overall
Heterogeneity P−value= 0.98
1.12 [1.08 , 1.16]
Smoking
Never
NCI 1
NCI 2
MDA
IARC GWAS
IARC Replication
Combined
Heterogeneity P−value= 0.0763
1.09 [0.90 , 1.31]
1.05 [0.91 , 1.22]
0.90 [0.71 , 1.13]
1.22 [1.12 , 1.34]
1.21 [1.07 , 1.38]
1.15 [1.08 , 1.22]
Former
NCI 1
NCI 2
MDA
IARC GWAS
IARC Replication
Combined
Heterogeneity P−value= 0.2781
1.04 [0.88 , 1.22]
1.02 [0.86 , 1.21]
1.11 [0.86 , 1.43]
1.22 [1.08 , 1.37]
1.00 [0.83 , 1.21]
1.10 [1.02 , 1.19]
Current
NCI 1
NCI 2
MDA
IARC GWAS
IARC Replication
Combined
Heterogeneity P−value= 0.0975
1.29 [1.07 , 1.55]
1.20 [0.90 , 1.61]
1.29 [0.87 , 1.93]
1.05 [0.93 , 1.18]
0.93 [0.78 , 1.11]
1.08 [1.00 , 1.18]
Overall
Heterogeneity P−value= 0.4365
1.12 [1.08 , 1.17]
0.50
1.00
1.50
2.00
Odds Ratio

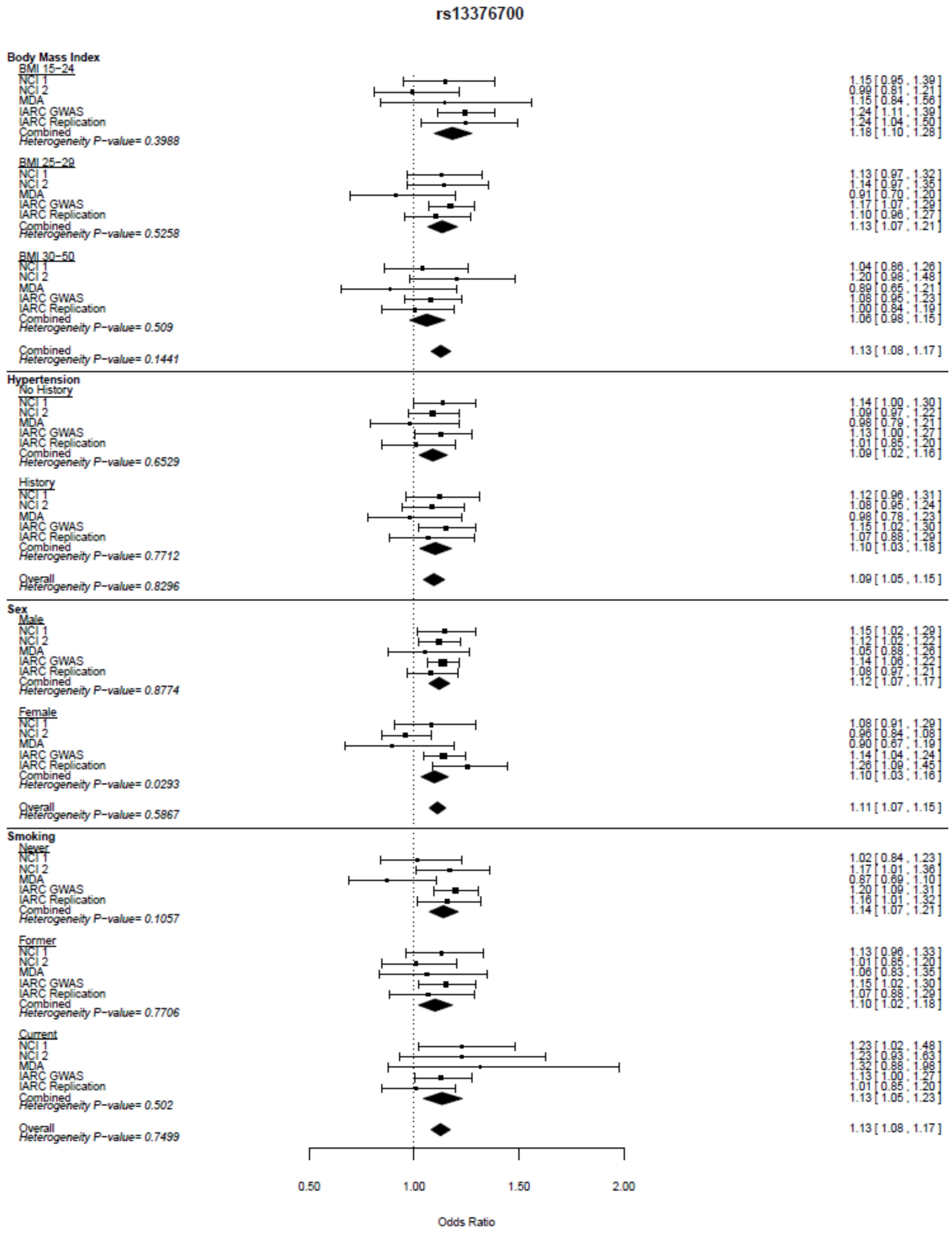
rs13376700
Body Mass Index
BMI 15−24
NCI 1 1.15 [0.95 , 1.39]
NCI 2 0.99 [0.81 , 1.21]
MDA 1.15 [0.84 , 1.56]
IARC GWAS 1.24 [1.11 , 1.39]
IARC Replication 1.24 [1.04 , 1.50]
Combined 1.18 [1.10 , 1.28]
Heterogeneity P−value= 0.3988
BMI 25−29
NCI 1 1.13 [0.97 , 1.32]
NCI 2 1.14 [0.97 , 1.35]
MDA 0.91 [0.70 , 1.20]
IARC GWAS 1.17 [1.07 , 1.29]
IARC Replication 1.10 [0.96 , 1.27]
Combined 1.13 [1.07 , 1.21]
Heterogeneity P−value= 0.5258
BMI 30−50
NCI 1 1.04 [0.86 , 1.26]
NCI 2 1.20 [0.98 , 1.48]
MDA 0.89 [0.65 , 1.21]
IARC GWAS 1.08 [0.95 , 1.23]
IARC Replication 1.00 [0.84 , 1.19]
Combined 1.06 [0.98 , 1.15]
Heterogeneity P−value= 0.509
Combined 1.13 [1.08 , 1.17]
Heterogeneity P−value= 0.1441
Hypertension
No History
NCI 1 1.14 [1.00 , 1.30]
NCI 2 1.09 [0.97 , 1.22]
MDA 0.98 [0.79 , 1.21]
IARC GWAS 1.13 [1.00 , 1.27]
IARC Replication 1.01 [0.85 , 1.20]
Combined 1.09 [1.02 , 1.16]
Heterogeneity P−value= 0.6529
History
NCI 1 1.12 [0.96 , 1.31]
NCI 2 1.08 [0.95 , 1.24]
MDA 0.98 [0.78 , 1.23]
IARC GWAS 1.15 [1.02 , 1.30]
IARC Replication 1.07 [0.88 , 1.29]
Combined 1.10 [1.03 , 1.18]
Heterogeneity P−value= 0.7712
Overall 1.09 [1.05 , 1.15]
Heterogeneity P−value= 0.8296
Sex
Male
NCI 1 1.15 [1.02 , 1.29]
NCI 2 1.12 [1.02 , 1.22]
MDA 1.05 [0.88 , 1.26]
IARC GWAS 1.14 [1.06 , 1.22]
IARC Replication 1.08 [0.97 , 1.21]
Combined 1.12 [1.07 , 1.17]
Heterogeneity P−value= 0.8774
Female
NCI 1 1.08 [0.91 , 1.29]
NCI 2 0.96 [0.84 , 1.08]
MDA 0.90 [0.67 , 1.19]
IARC GWAS 1.14 [1.04 , 1.24]
IARC Replication 1.26 [1.09 , 1.45]
Combined 1.10 [1.03 , 1.16]
Heterogeneity P−value= 0.0293
Overall 1.11 [1.07 , 1.15]
Heterogeneity P−value= 0.5867
Smoking
Never
NCI 1 1.02 [0.84 , 1.23]
NCI 2 1.17 [1.01 , 1.36]
MDA 0.87 [0.69 , 1.10]
IARC GWAS 1.20 [1.09 , 1.31]
IARC Replication 1.16 [1.01 , 1.32]
Combined 1.14 [1.07 , 1.21]
Heterogeneity P−value= 0.1057
Former
NCI 1 1.13 [0.96 , 1.33]
NCI 2 1.01 [0.85 , 1.20]
MDA 1.06 [0.83 , 1.35]
IARC GWAS 1.15 [1.02 , 1.30]
IARC Replication 1.07 [0.88 , 1.29]
Combined 1.10 [1.02 , 1.18]
Heterogeneity P−value= 0.7706
Current
NCI 1 1.23 [1.02 , 1.48]
NCI 2 1.23 [0.93 , 1.63]
MDA 1.32 [0.88 , 1.98]
IARC GWAS 1.13 [1.00 , 1.27]
IARC Replication 1.01 [0.85 , 1.20]
Combined 1.13 [1.05 , 1.23]
Heterogeneity P−value= 0.502
Overall 1.13 [1.08 , 1.17]
Heterogeneity P−value= 0.7499
0.50
1.00
1.50
2.00
Odds Ratio

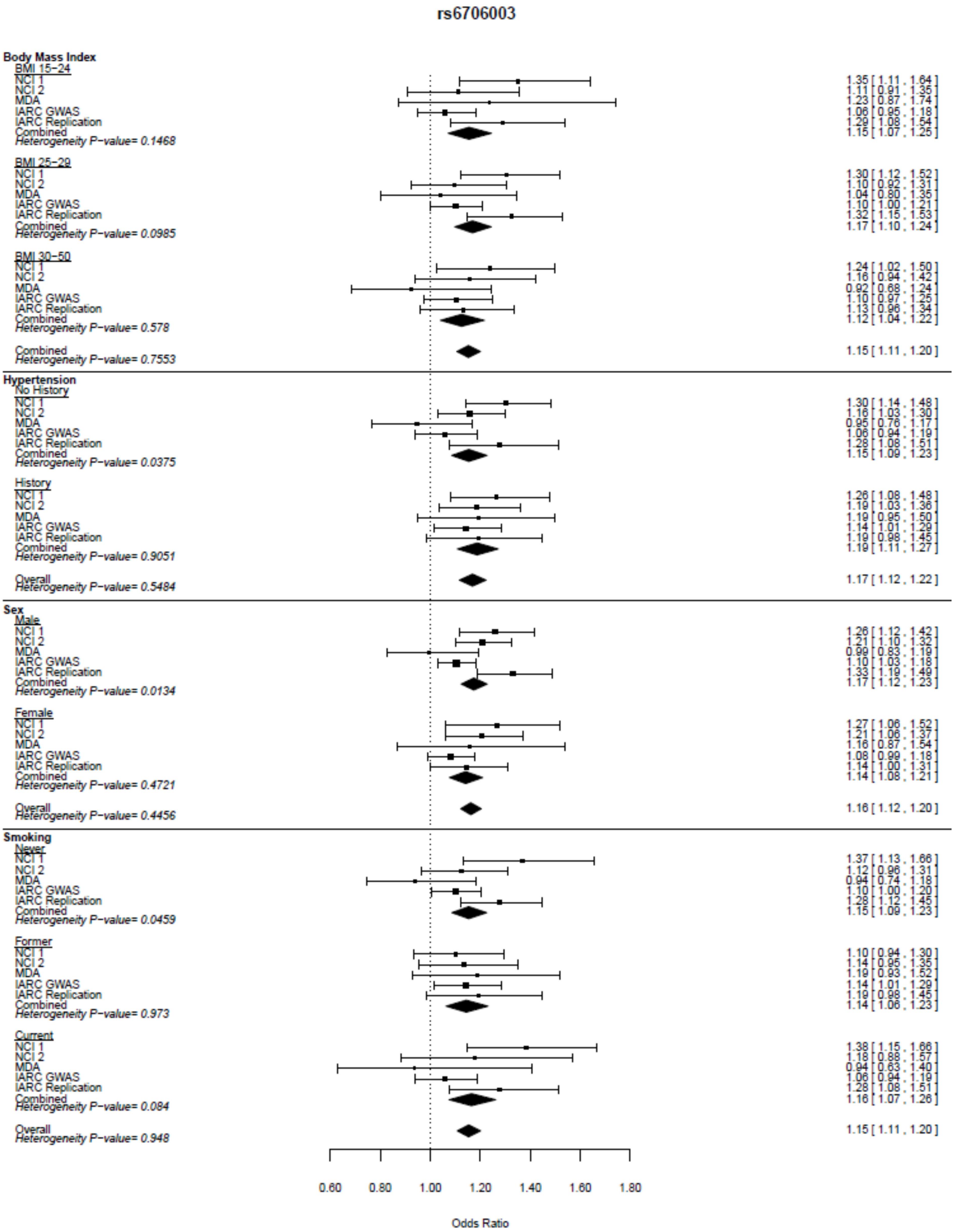

rs6706003
Body Mass Index
BMI 15−24
NCI 1 1.35 [1.11 , 1.64]
NCI 2 1.11 [0.91 , 1.35]
MDA 1.23 [0.87 , 1.74]
IARC GWAS 1.06 [0.95 , 1.18]
IARC Replication 1.29 [1.08 , 1.54]
Combined 1.15 [1.07 , 1.25]
Heterogeneity P−value= 0.1468
BMI 25−29
NCI 1 1.30 [1.12 , 1.52]
NCI 2 1.10 [0.92 , 1.31]
MDA 1.04 [0.80 , 1.35]
IARC GWAS 1.10 [1.00 , 1.21]
IARC Replication 1.32 [1.15 , 1.53]
Combined 1.17 [1.10 , 1.24]
Heterogeneity P−value= 0.0985
BMI 30−50
NCI 1 1.24 [1.02 , 1.50]
NCI 2 1.16 [0.94 , 1.42]
MDA 0.92 [0.68 , 1.24]
IARC GWAS 1.10 [0.97 , 1.25]
IARC Replication 1.13 [0.96 , 1.34]
Combined 1.12 [1.04 , 1.22]
Heterogeneity P−value= 0.578
Combined 1.15 [1.11 , 1.20]
Heterogeneity P−value= 0.7553
Hypertension
No History
NCI 1 1.30 [1.14 , 1.48]
NCI 2 1.16 [1.03 , 1.30]
MDA 0.95 [0.76 , 1.17]
IARC GWAS 1.06 [0.94 , 1.19]
IARC Replication 1.28 [1.08 , 1.51]
Combined 1.15 [1.09 , 1.23]
Heterogeneity P−value= 0.0375
History
NCI 1 1.26 [1.08 , 1.48]
NCI 2 1.19 [1.03 , 1.36]
MDA 1.19 [0.95 , 1.50]
IARC GWAS 1.14 [1.01 , 1.29]
IARC Replication 1.19 [0.98 , 1.45]
Combined 1.19 [1.11 , 1.27]
Heterogeneity P−value= 0.9051
Overall 1.17 [1.12 , 1.22]
Heterogeneity P−value= 0.5484
Sex
Male
NCI 1 1.26 [1.12 , 1.42]
NCI 2 1.21 [1.10 , 1.32]
MDA 0.99 [0.83 , 1.19]
IARC GWAS 1.10 [1.03 , 1.18]
IARC Replication 1.33 [1.19 , 1.49]
Combined 1.17 [1.12 , 1.23]
Heterogeneity P−value= 0.0134
Female
NCI 1 1.27 [1.06 , 1.52]
NCI 2 1.21 [1.06 , 1.37]
MDA 1.16 [0.87 , 1.54]
IARC GWAS 1.08 [0.99 , 1.18]
IARC Replication 1.14 [1.00 , 1.31]
Combined 1.14 [1.08 , 1.21]
Heterogeneity P−value= 0.4721
Overall 1.16 [1.12 , 1.20]
Heterogeneity P−value= 0.4456
Smoking
Never
NCI 1 1.37 [1.13 , 1.66]
NCI 2 1.12 [0.96 , 1.31]
MDA 0.94 [0.74 , 1.18]
IARC GWAS 1.10 [1.00 , 1.20]
IARC Replication 1.28 [1.12 , 1.45]
Combined 1.15 [1.09 , 1.23]
Heterogeneity P−value= 0.0459
Former
NCI 1 1.10 [0.94 , 1.30]
NCI 2 1.14 [0.95 , 1.35]
MDA 1.19 [0.93 , 1.52]
IARC GWAS 1.14 [1.01 , 1.29]
IARC Replication 1.19 [0.98 , 1.45]
Combined 1.14 [1.06 , 1.23]
Heterogeneity P−value= 0.973
Current
NCI 1 1.38 [1.15 , 1.66]
NCI 2 1.18 [0.88 , 1.57]
MDA 0.94 [0.63 , 1.40]
IARC GWAS 1.06 [0.94 , 1.19]
IARC Replication 1.28 [1.08 , 1.51]
Combined 1.16 [1.07 , 1.26]
Heterogeneity P−value= 0.084
Overall 1.15 [1.11 , 1.20]
Heterogeneity P−value= 0.948
0.60 0.80 1.00 1.20 1.40 1.60 1.80
Odds Ratio

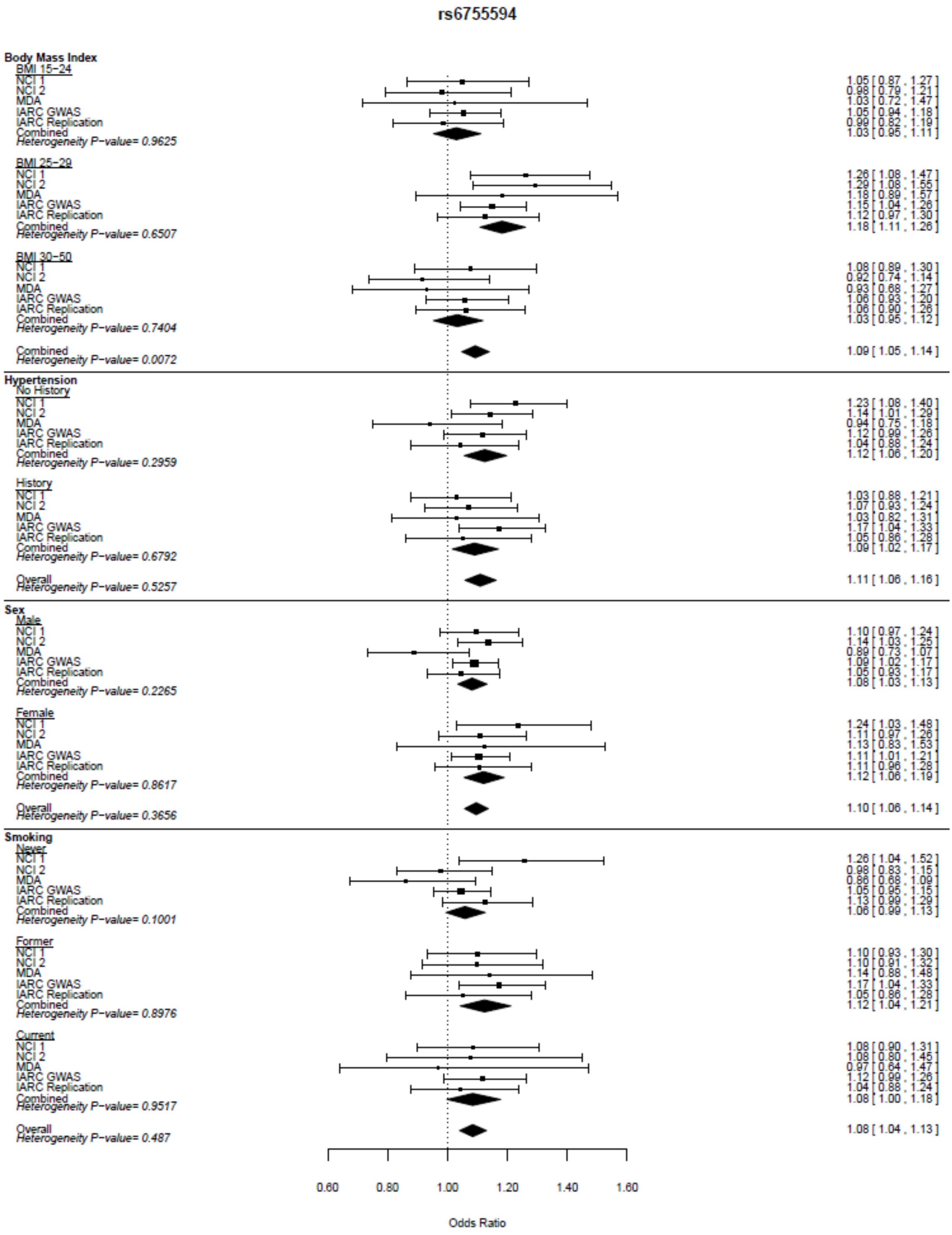
rs6755594
Body Mass Index
BMI 15−24
NCI 1
NCI 2
MDA
IARC GWAS
IARC Replication
Combined
Heterogeneity P−value= 0.9625
1.05 [0.87 , 1.27]
0.98 [0.79 , 1.21]
1.03 [0.72 , 1.47]
1.05 [0.94 , 1.18]
0.99 [0.82 , 1.19]
1.03 [0.95 , 1.11]
BMI 25−29
NCI 1
NCI 2
MDA
IARC GWAS
IARC Replication
Combined
Heterogeneity P−value= 0.6507
1.26 [1.08 , 1.47]
1.29 [1.08 , 1.55]
1.18 [0.89 , 1.57]
1.15 [1.04 , 1.26]
1.12 [0.97 , 1.30]
1.18 [1.11 , 1.26]
BMI 30−50
NCI 1
NCI 2
MDA
IARC GWAS
IARC Replication
Combined
Heterogeneity P−value= 0.7404
1.08 [0.89 , 1.30]
0.92 [0.74 , 1.14]
0.93 [0.68 , 1.27]
1.06 [0.93 , 1.20]
1.06 [0.90 , 1.26]
1.03 [0.95 , 1.12]
Combined
Heterogeneity P−value= 0.0072
1.09 [1.05 , 1.14]
Hypertension
No History
NCI 1
NCI 2
MDA
IARC GWAS
IARC Replication
Combined
Heterogeneity P−value= 0.2959
1.23 [1.08 , 1.40]
1.14 [1.01 , 1.29]
0.94 [0.75 , 1.18]
1.12 [0.99 , 1.26]
1.04 [0.88 , 1.24]
1.12 [1.06 , 1.20]
History
NCI 1
NCI 2
MDA
IARC GWAS
IARC Replication
Combined
Heterogeneity P−value= 0.6792
1.03 [0.88 , 1.21]
1.07 [0.93 , 1.24]
1.03 [0.82 , 1.31]
1.17 [1.04 , 1.33]
1.05 [0.86 , 1.28]
1.09 [1.02 , 1.17]
Overall
Heterogeneity P−value= 0.5257
1.11 [1.06 , 1.16]
Sex
Male
NCI 1
NCI 2
MDA
IARC GWAS
IARC Replication
Combined
Heterogeneity P−value= 0.2265
1.10 [0.97 , 1.24]
1.14 [1.03 , 1.25]
0.89 [0.73 , 1.07]
1.09 [1.02 , 1.17]
1.05 [0.93 , 1.17]
1.08 [1.03 , 1.13]
Female
NCI 1
NCI 2
MDA
IARC GWAS
IARC Replication
Combined
Heterogeneity P−value= 0.8617
1.24 [1.03 , 1.48]
1.11 [0.97 , 1.26]
1.13 [0.83 , 1.53]
1.11 [1.01 , 1.21]
1.11 [0.96 , 1.28]
1.12 [1.06 , 1.19]
Overall
Heterogeneity P−value= 0.3656
1.10 [1.06 , 1.14]
Smoking
Never
NCI 1
NCI 2
MDA
IARC GWAS
IARC Replication
Combined
Heterogeneity P−value= 0.1001
1.26 [1.04 , 1.52]
0.98 [0.83 , 1.15]
0.86 [0.68 , 1.09]
1.05 [0.95 , 1.15]
1.13 [0.99 , 1.29]
1.06 [0.99 , 1.13]
Former
NCI 1
NCI 2
MDA
IARC GWAS
IARC Replication
Combined
Heterogeneity P−value= 0.8976
1.10 [0.93 , 1.30]
1.10 [0.91 , 1.32]
1.14 [0.88 , 1.48]
1.17 [1.04 , 1.33]
1.05 [0.86 , 1.28]
1.12 [1.04 , 1.21]
Current
NCI 1
NCI 2
MDA
IARC GWAS
IARC Replication
Combined
Heterogeneity P−value= 0.9517
1.08 [0.90 , 1.31]
1.08 [0.80 , 1.45]
0.97 [0.64 , 1.47]
1.12 [0.99 , 1.26]
1.04 [0.88 , 1.24]
1.08 [1.00 , 1.18]
Overall
Heterogeneity P−value= 0.487
1.08 [1.04 , 1.13]
0.60
0.80
1.00
1.20
1.40
1.60
Odds Ratio

rs72851889

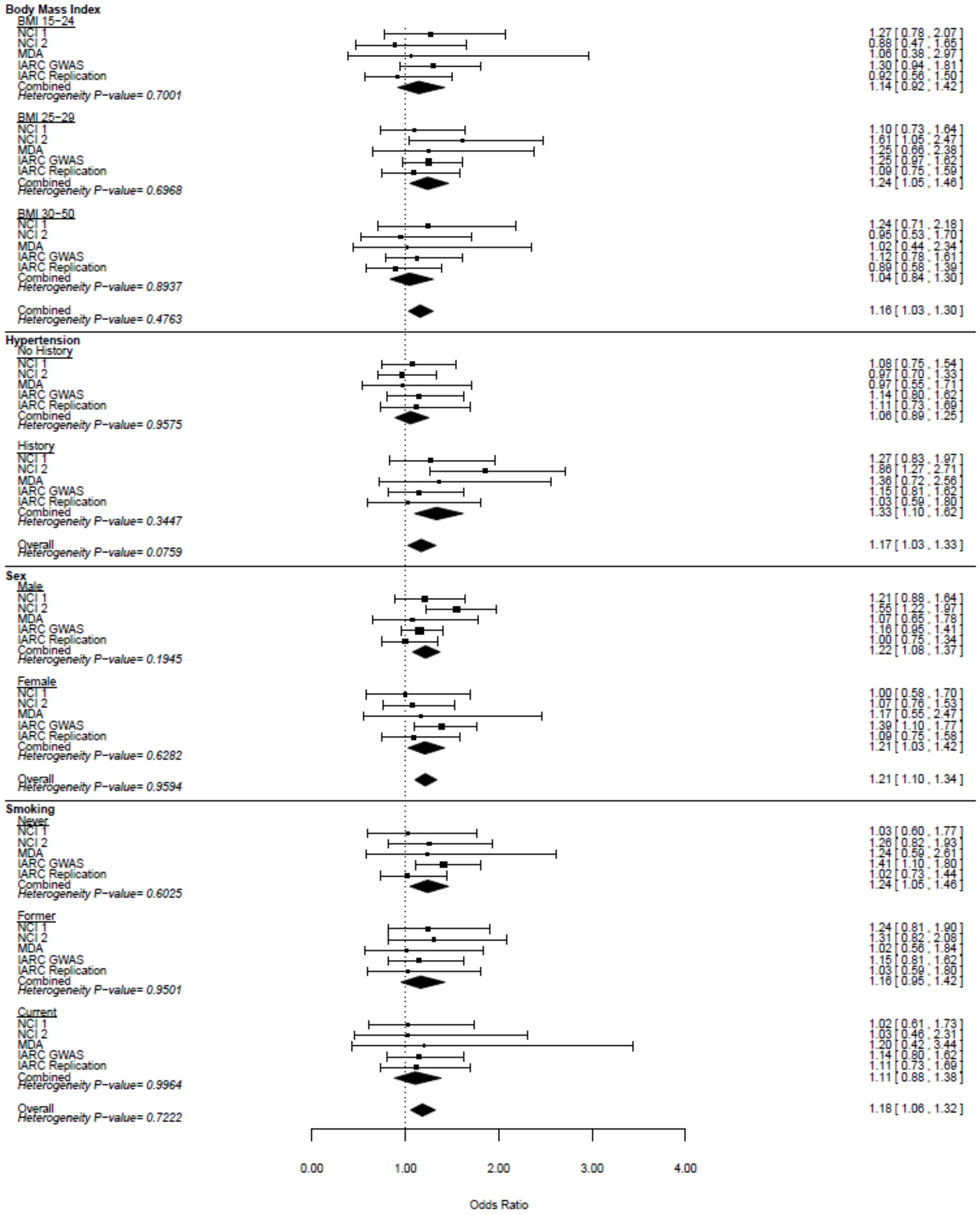

Body Mass Index
BMI 15−24
NCI 1 1.27 [0.78 , 2.07]
NCI 2 0.88 [0.47 , 1.65]
MDA 1.06 [0.38 , 2.97]
IARC GWAS 1.30 [0.94 , 1.81]
IARC Replication 0.92 [0.56 , 1.50]
Combined 1.14 [0.92 , 1.42]
Heterogeneity P−value= 0.7001
BMI 25−29
NCI 1 1.10 [0.73 , 1.64]
NCI 2 1.61 [1.05 , 2.47]
MDA 1.25 [0.66 , 2.38]
IARC GWAS 1.25 [0.97 , 1.62]
IARC Replication 1.09 [0.75 , 1.59]
Combined 1.24 [1.05 , 1.46]
Heterogeneity P−value= 0.6968
BMI 30−50
NCI 1 1.24 [0.71 , 2.18]
NCI 2 0.95 [0.53 , 1.70]
MDA 1.02 [0.44 , 2.34]
IARC GWAS 1.12 [0.78 , 1.61]
IARC Replication 0.89 [0.58 , 1.39]
Combined 1.04 [0.84 , 1.30]
Heterogeneity P−value= 0.8937
Combined 1.16 [1.03 , 1.30]
Heterogeneity P−value= 0.4763
Hypertension
No History
NCI 1 1.08 [0.75 , 1.54]
NCI 2 0.97 [0.70 , 1.33]
MDA 0.97 [0.55 , 1.71]
IARC GWAS 1.14 [0.80 , 1.62]
IARC Replication 1.11 [0.73 , 1.69]
Combined 1.06 [0.89 , 1.25]
Heterogeneity P−value= 0.9575
History
NCI 1 1.27 [0.83 , 1.97]
NCI 2 1.86 [1.27 , 2.71]
MDA 1.36 [0.72 , 2.56]
IARC GWAS 1.15 [0.81 , 1.62]
IARC Replication 1.03 [0.59 , 1.80]
Combined 1.33 [1.10 , 1.62]
Heterogeneity P−value= 0.3447
Overall 1.17 [1.03 , 1.33]
Heterogeneity P−value= 0.0759
Sex
Male
NCI 1 1.21 [0.88 , 1.64]
NCI 2 1.55 [1.22 , 1.97]
MDA 1.07 [0.65 , 1.78]
IARC GWAS 1.16 [0.95 , 1.41]
IARC Replication 1.00 [0.75 , 1.34]
Combined 1.22 [1.08 , 1.37]
Heterogeneity P−value= 0.1945
Female
NCI 1 1.00 [0.58 , 1.70]
NCI 2 1.07 [0.76 , 1.53]
MDA 1.17 [0.55 , 2.47]
IARC GWAS 1.39 [1.10 , 1.77]
IARC Replication 1.09 [0.75 , 1.58]
Combined 1.21 [1.03 , 1.42]
Heterogeneity P−value= 0.6282
Overall 1.21 [1.10 , 1.34]
Heterogeneity P−value= 0.9594
Smoking
Never
NCI 1 1.03 [0.60 , 1.77]
NCI 2 1.26 [0.82 , 1.93]
MDA 1.24 [0.59 , 2.61]
IARC GWAS 1.41 [1.10 , 1.80]
IARC Replication 1.02 [0.73 , 1.44]
Combined 1.24 [1.05 , 1.46]
Heterogeneity P−value= 0.6025
Former
NCI 1 1.24 [0.81 , 1.90]
NCI 2 1.31 [0.82 , 2.08]
MDA 1.02 [0.56 , 1.84]
IARC GWAS 1.15 [0.81 , 1.62]
IARC Replication 1.03 [0.59 , 1.80]
Combined 1.16 [0.95 , 1.42]
Heterogeneity P−value= 0.9501
Current
NCI 1 1.02 [0.61 , 1.73]
NCI 2 1.03 [0.46 , 2.31]
MDA 1.20 [0.42 , 3.44]
IARC GWAS 1.14 [0.80 , 1.62]
IARC Replication 1.11 [0.73 , 1.69]
Combined 1.11 [0.88 , 1.38]
Heterogeneity P−value= 0.9964
Overall 1.18 [1.06 , 1.32]
Heterogeneity P−value= 0.7222
0.00
1.00
2.00
3.00
4.00
Odds Ratio

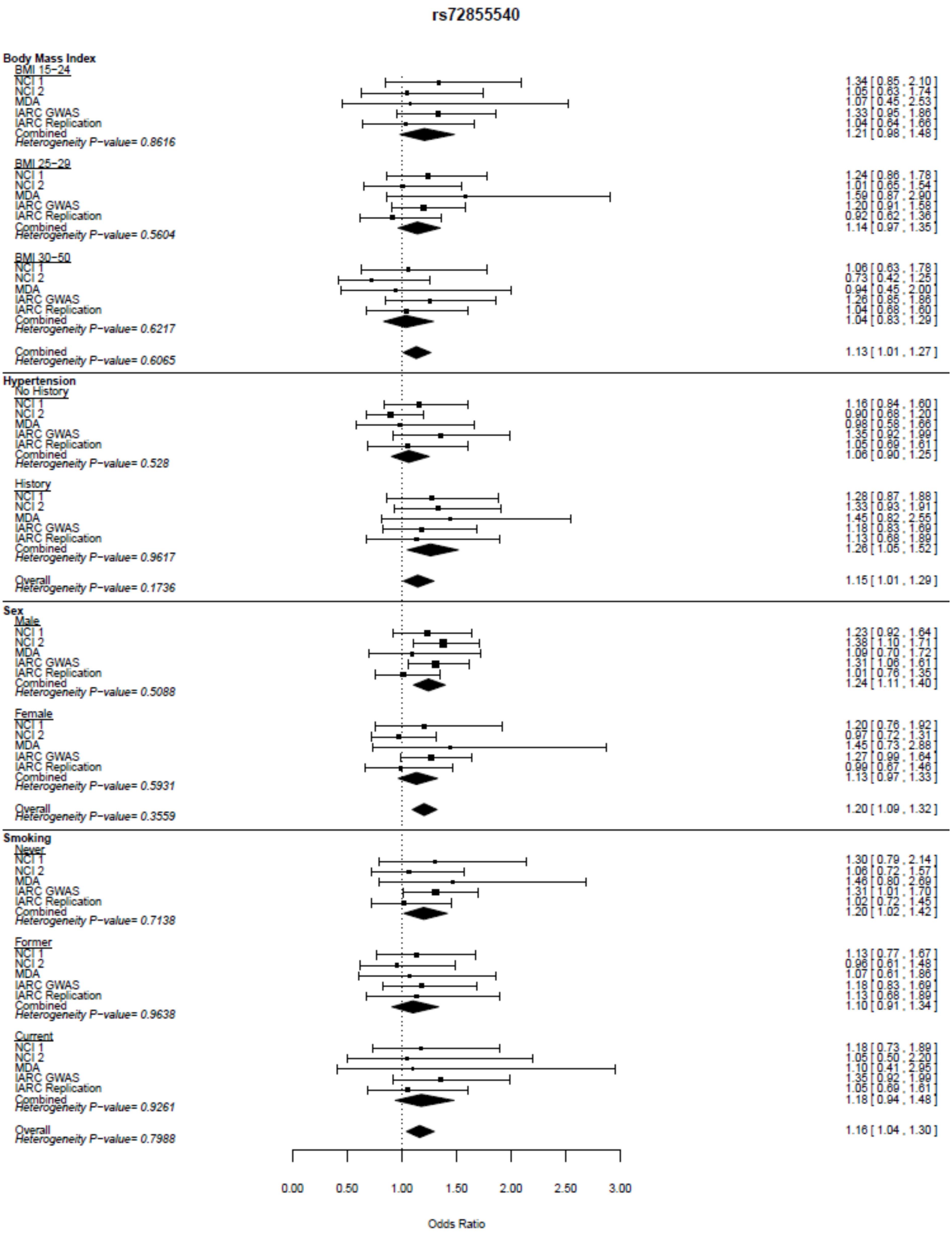

rs72855540
Body Mass Index
BMI 15−24
NCI 1
NCI 2
MDA
IARC GWAS
IARC Replication
Combined
Heterogeneity P−value= 0.8616
BMI 25−29
Heterogeneity P−value= 0.5604
BMI 30−50
Heterogeneity P−value= 0.6217
Combined
Heterogeneity P−value= 0.6065
Hypertension
No History
Heterogeneity P−value= 0.528
History
Heterogeneity P−value= 0.9617
Overall
Heterogeneity P−value= 0.1736
Sex
Male
Heterogeneity P−value= 0.5088
Female
Heterogeneity P−value= 0.5931
Overall
Heterogeneity P−value= 0.3559
Smoking
Never
Heterogeneity P−value= 0.7138
Former
Heterogeneity P−value= 0.9638
Current
Heterogeneity P−value= 0.9261
Overall
Heterogeneity P−value= 0.7988
1.34 [0.85 , 2.10]
1.05 [0.63 , 1.74]
1.07 [0.45 , 2.53]
1.33 [0.95 , 1.86]
1.04 [0.64 , 1.66]
1.21 [0.98 , 1.48]
1.24 [0.86 , 1.78]
1.01 [0.66 , 1.54]
1.59 [0.87 , 2.90]
1.20 [0.91 , 1.58]
0.92 [0.62 , 1.36]
1.14 [0.97 , 1.35]
1.06 [0.63 , 1.78]
0.73 [0.42 , 1.25]
0.94 [0.45 , 2.00]
1.26 [0.85 , 1.86]
1.04 [0.68 , 1.60]
1.04 [0.83 , 1.29]
1.13 [1.01 , 1.27]
1.16 [0.84 , 1.60]
0.90 [0.68 , 1.20]
0.98 [0.58 , 1.66]
1.35 [0.92 , 1.99]
1.05 [0.69 , 1.61]
1.06 [0.90 , 1.25]
1.28 [0.87 , 1.88]
1.33 [0.93 , 1.91]
1.45 [0.82 , 2.55]
1.18 [0.83 , 1.69]
1.13 [0.68 , 1.89]
1.26 [1.05 , 1.52]
1.15 [1.01 , 1.29]
1.23 [0.92 , 1.64]
1.38 [1.10 , 1.71]
1.09 [0.70 , 1.72]
1.31 [1.06 , 1.61]
1.01 [0.76 , 1.35]
1.24 [1.11 , 1.40]
1.20 [0.76 , 1.92]
0.97 [0.72 , 1.31]
1.45 [0.73 , 2.88]
1.27 [0.99 , 1.64]
0.99 [0.67 , 1.46]
1.13 [0.97 , 1.33]
1.20 [1.09 , 1.32]
1.30 [0.79 , 2.14]
1.06 [0.72 , 1.57]
1.46 [0.80 , 2.69]
1.31 [1.01 , 1.70]
1.02 [0.72 , 1.45]
1.20 [1.02 , 1.42]
1.13 [0.77 , 1.67]
0.96 [0.61 , 1.48]
1.07 [0.61 , 1.86]
1.18 [0.83 , 1.69]
1.13 [0.68 , 1.89]
1.10 [0.91 , 1.34]
1.18 [0.73 , 1.89]
1.05 [0.50 , 2.20]
1.10 [0.41 , 2.95]
1.35 [0.92 , 1.99]
1.05 [0.69 , 1.61]
1.18 [0.94 , 1.48]
1.16 [1.04 , 1.30]
0.00
0.50
1.00
1.50
2.00
2.50
3.00
Odds Ratio

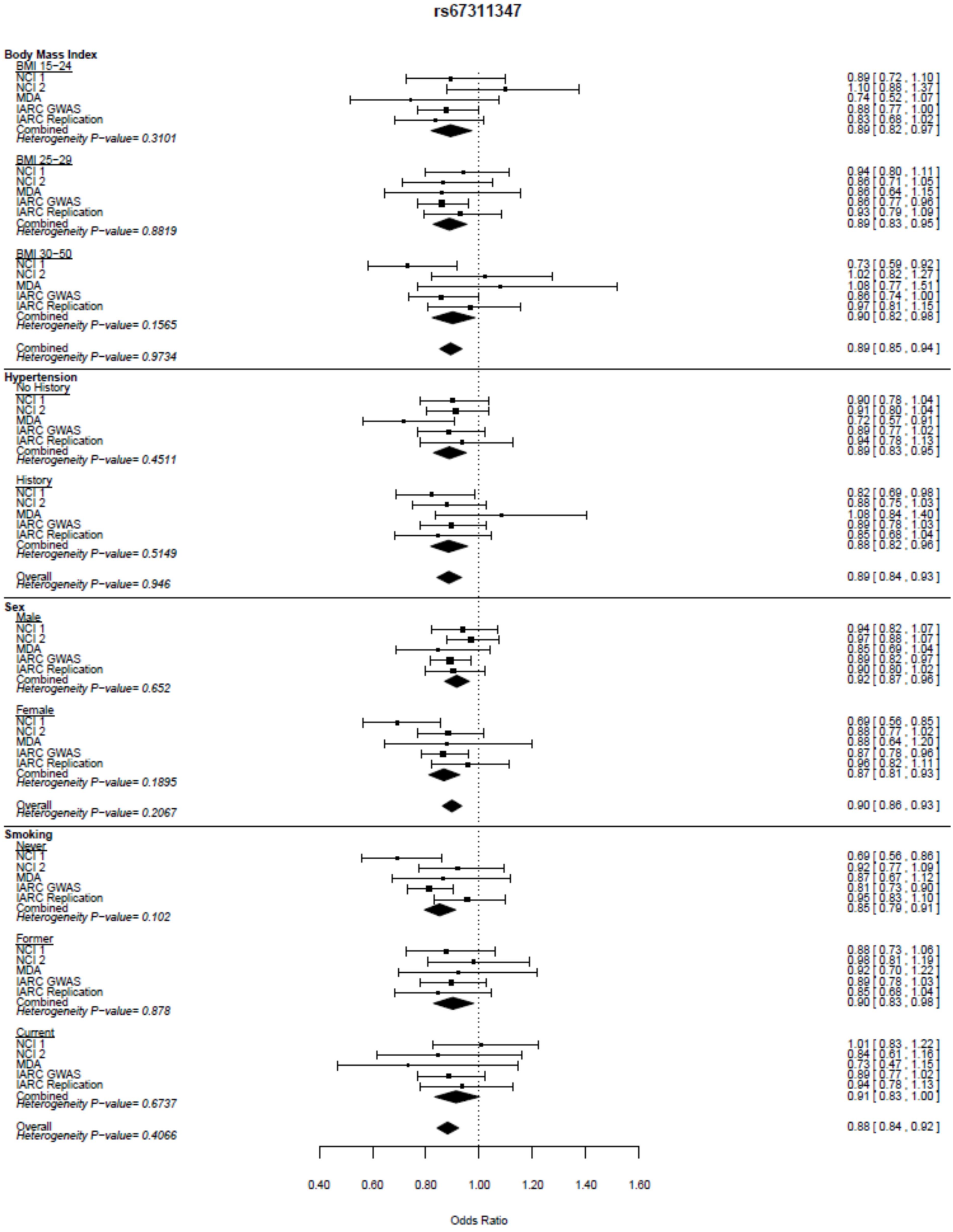
rs67311347
Body Mass Index
BMI 15−24
NCI 1
NCI 2
MDA
IARC GWAS
IARC Replication
Combined
Heterogeneity P−value= 0.3101
0.89 [0.72 , 1.10]
1.10 [0.88 , 1.37]
0.74 [0.52 , 1.07]
0.88 [0.77 , 1.00]
0.83 [0.68 , 1.02]
0.89 [0.82 , 0.97]
BMI 25−29
NCI 1
NCI 2
MDA
IARC GWAS
IARC Replication
Combined
Heterogeneity P−value= 0.8819
0.94 [0.80 , 1.11]
0.86 [0.71 , 1.05]
0.86 [0.64 , 1.15]
0.86 [0.77 , 0.96]
0.93 [0.79 , 1.09]
0.89 [0.83 , 0.95]
BMI 30−50
NCI 1
NCI 2
MDA
IARC GWAS
IARC Replication
Combined
Heterogeneity P−value= 0.1565
0.73 [0.59 , 0.92]
1.02 [0.82 , 1.27]
1.08 [0.77 , 1.51]
0.86 [0.74 , 1.00]
0.97 [0.81 , 1.15]
0.90 [0.82 , 0.98]
Combined
Heterogeneity P−value= 0.9734
0.89 [0.85 , 0.94]
Hypertension
No History
NCI 1
NCI 2
MDA
IARC GWAS
IARC Replication
Combined
Heterogeneity P−value= 0.4511
0.90 [0.78 , 1.04]
0.91 [0.80 , 1.04]
0.72 [0.57 , 0.91]
0.89 [0.77 , 1.02]
0.94 [0.78 , 1.13]
0.89 [0.83 , 0.95]
History
NCI 1
NCI 2
MDA
IARC GWAS
IARC Replication
Combined
Heterogeneity P−value= 0.5149
0.82 [0.69 , 0.98]
0.88 [0.75 , 1.03]
1.08 [0.84 , 1.40]
0.89 [0.78 , 1.03]
0.85 [0.68 , 1.04]
0.88 [0.82 , 0.96]
Overall
Heterogeneity P−value= 0.946
0.89 [0.84 , 0.93]
Sex
Male
NCI 1
NCI 2
MDA
IARC GWAS
IARC Replication
Combined
Heterogeneity P−value= 0.652
0.94 [0.82 , 1.07]
0.97 [0.88 , 1.07]
0.85 [0.69 , 1.04]
0.89 [0.82 , 0.97]
0.90 [0.80 , 1.02]
0.92 [0.87 , 0.96]
Female
NCI 1
NCI 2
MDA
IARC GWAS
IARC Replication
Combined
Heterogeneity P−value= 0.1895
0.69 [0.56 , 0.85]
0.88 [0.77 , 1.02]
0.88 [0.64 , 1.20]
0.87 [0.78 , 0.96]
0.96 [0.82 , 1.11]
0.87 [0.81 , 0.93]
Overall
Heterogeneity P−value= 0.2067
0.90 [0.86 , 0.93]
Smoking
Never
NCI 1
NCI 2
MDA
IARC GWAS
IARC Replication
Combined
Heterogeneity P−value= 0.102
0.69 [0.56 , 0.86]
0.92 [0.77 , 1.09]
0.87 [0.67 , 1.12]
0.81 [0.73 , 0.90]
0.95 [0.83 , 1.10]
0.85 [0.79 , 0.91]
Former
NCI 1
NCI 2
MDA
IARC GWAS
IARC Replication
Combined
Heterogeneity P−value= 0.878
0.88 [0.73 , 1.06]
0.98 [0.81 , 1.19]
0.92 [0.70 , 1.22]
0.89 [0.78 , 1.03]
0.85 [0.68 , 1.04]
0.90 [0.83 , 0.98]
Current
NCI 1
NCI 2
MDA
IARC GWAS
IARC Replication
Combined
Heterogeneity P−value= 0.6737
1.01 [0.83 , 1.22]
0.84 [0.61 , 1.16]
0.73 [0.47 , 1.15]
0.89 [0.77 , 1.02]
0.94 [0.78 , 1.13]
0.91 [0.83 , 1.00]
Overall
Heterogeneity P−value= 0.4066
0.88 [0.84 , 0.92]
0.40
0.60
0.80
1.00
1.20
1.40
1.60
Odds Ratio

rs2203002

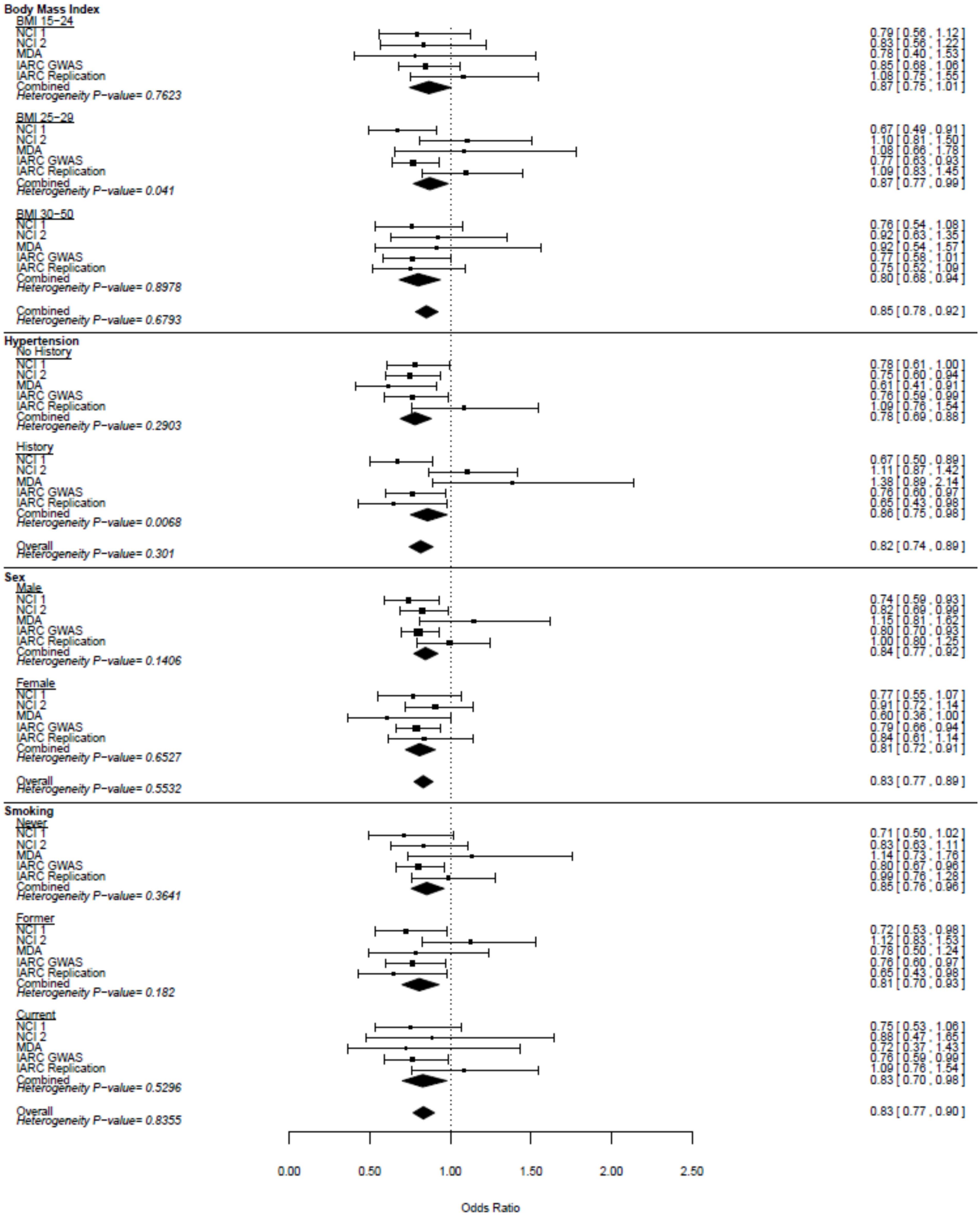
Body Mass Index
BMI 15−24
NCI 1 0.79 [0.56 , 1.12]
NCI 2 0.83 [0.56 , 1.22]
MDA 0.78 [0.40 , 1.53]
IARC GWAS 0.85 [0.68 , 1.06]
IARC Replication 1.08 [0.75 , 1.55]
Combined 0.87 [0.75 , 1.01]
Heterogeneity P−value= 0.7623
BMI 25−29
NCI 1 0.67 [0.49 , 0.91]
NCI 2 1.10 [0.81 , 1.50]
MDA 1.08 [0.66 , 1.78]
IARC GWAS 0.77 [0.63 , 0.93]
IARC Replication 1.09 [0.83 , 1.45]
Combined 0.87 [0.77 , 0.99]
Heterogeneity P−value= 0.041
BMI 30−50
NCI 1 0.76 [0.54 , 1.08]
NCI 2 0.92 [0.63 , 1.35]
MDA 0.92 [0.54 , 1.57]
IARC GWAS 0.77 [0.58 , 1.01]
IARC Replication 0.75 [0.52 , 1.09]
Combined 0.80 [0.68 , 0.94]
Heterogeneity P−value= 0.8978
Combined 0.85 [0.78 , 0.92]
Heterogeneity P−value= 0.6793
Hypertension
No History
NCI 1 0.78 [0.61 , 1.00]
NCI 2 0.75 [0.60 , 0.94]
MDA 0.61 [0.41 , 0.91]
IARC GWAS 0.76 [0.59 , 0.99]
IARC Replication 1.09 [0.76 , 1.54]
Combined 0.78 [0.69 , 0.88]
Heterogeneity P−value= 0.2903
History
NCI 1 0.67 [0.50 , 0.89]
NCI 2 1.11 [0.87 , 1.42]
MDA 1.38 [0.89 , 2.14]
IARC GWAS 0.76 [0.60 , 0.97]
IARC Replication 0.65 [0.43 , 0.98]
Combined 0.86 [0.75 , 0.98]
Heterogeneity P−value= 0.0068
Overall 0.82 [0.74 , 0.89]
Heterogeneity P−value= 0.301
Sex
Male
NCI 1 0.74 [0.59 , 0.93]
NCI 2 0.82 [0.69 , 0.99]
MDA 1.15 [0.81 , 1.62]
IARC GWAS 0.80 [0.70 , 0.93]
IARC Replication 1.00 [0.80 , 1.25]
Combined 0.84 [0.77 , 0.92]
Heterogeneity P−value= 0.1406
Female
NCI 1 0.77 [0.55 , 1.07]
NCI 2 0.91 [0.72 , 1.14]
MDA 0.60 [0.36 , 1.00]
IARC GWAS 0.79 [0.66 , 0.94]
IARC Replication 0.84 [0.61 , 1.14]
Combined 0.81 [0.72 , 0.91]
Heterogeneity P−value= 0.6527
Overall 0.83 [0.77 , 0.89]
Heterogeneity P−value= 0.5532
Smoking
Never
NCI 1 0.71 [0.50 , 1.02]
NCI 2 0.83 [0.63 , 1.11]
MDA 1.14 [0.73 , 1.76]
IARC GWAS 0.80 [0.67 , 0.96]
IARC Replication 0.99 [0.76 , 1.28]
Combined 0.85 [0.76 , 0.96]
Heterogeneity P−value= 0.3641
Former
NCI 1 0.72 [0.53 , 0.98]
NCI 2 1.12 [0.83 , 1.53]
MDA 0.78 [0.50 , 1.24]
IARC GWAS 0.76 [0.60 , 0.97]
IARC Replication 0.65 [0.43 , 0.98]
Combined 0.81 [0.70 , 0.93]
Heterogeneity P−value= 0.182
Current
NCI 1 0.75 [0.53 , 1.06]
NCI 2 0.88 [0.47 , 1.65]
MDA 0.72 [0.37 , 1.43]
IARC GWAS 0.76 [0.59 , 0.99]
IARC Replication 1.09 [0.76 , 1.54]
Combined 0.83 [0.70 , 0.98]
Heterogeneity P−value= 0.5296
Overall 0.83 [0.77 , 0.90]
Heterogeneity P−value= 0.8355
0.00
0.50
1.00
1.50
2.00
2.50
Odds Ratio

rs10936602

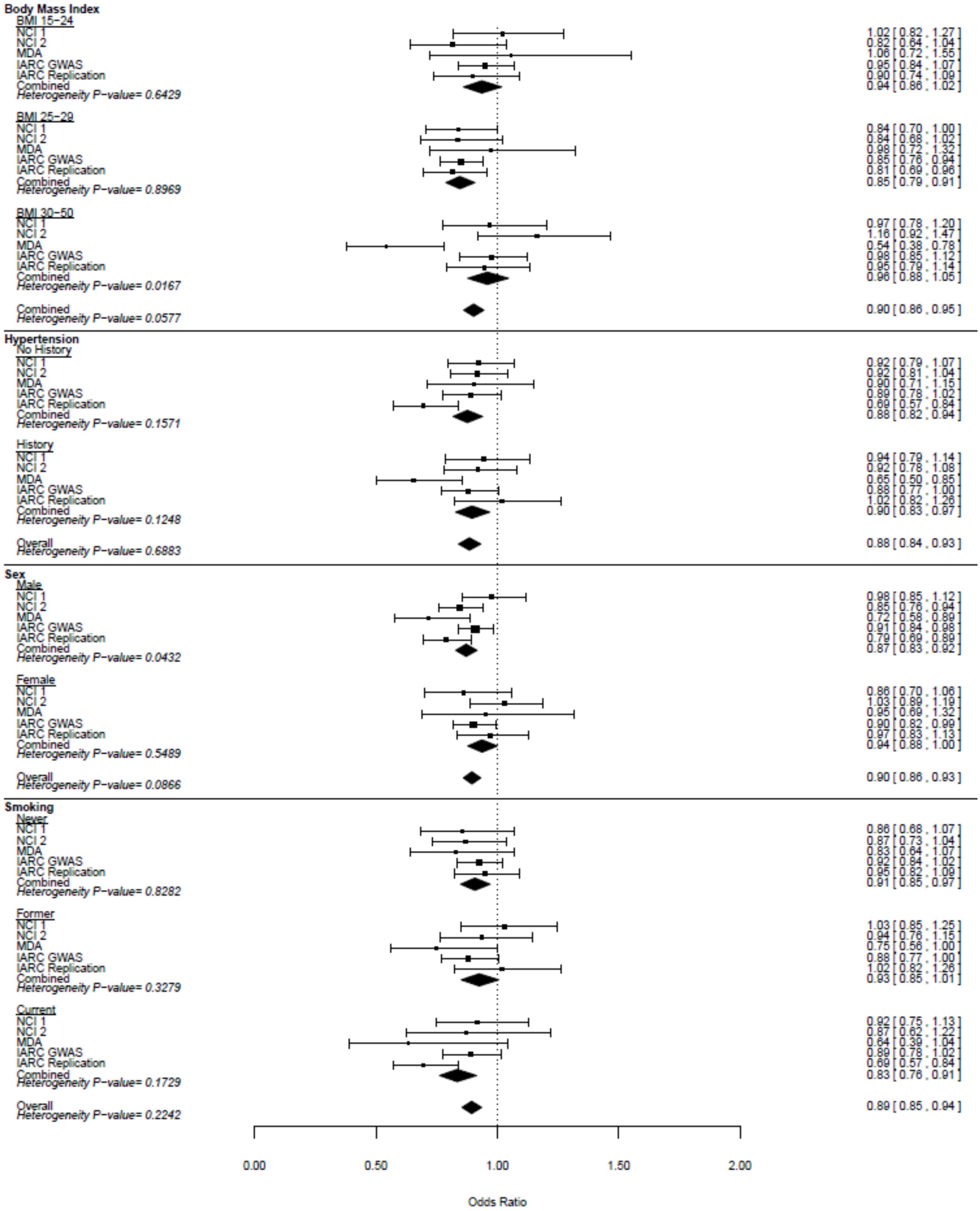
Body Mass Index
BMI 15−24
NCI 1 1.02 [0.82 , 1.27]
NCI 2 0.82 [0.64 , 1.04]
MDA 1.06 [0.72 , 1.55]
IARC GWAS 0.95 [0.84 , 1.07]
IARC Replication 0.90 [0.74 , 1.09]
Combined 0.94 [0.86 , 1.02]
Heterogeneity P−value= 0.6429
BMI 25−29
NCI 1 0.84 [0.70 , 1.00]
NCI 2 0.84 [0.68 , 1.02]
MDA 0.98 [0.72 , 1.32]
IARC GWAS 0.85 [0.76 , 0.94]
IARC Replication 0.81 [0.69 , 0.96]
Combined 0.85 [0.79 , 0.91]
Heterogeneity P−value= 0.8969
BMI 30−50
NCI 1 0.97 [0.78 , 1.20]
NCI 2 1.16 [0.92 , 1.47]
MDA 0.54 [0.38 , 0.78]
IARC GWAS 0.98 [0.85 , 1.12]
IARC Replication 0.95 [0.79 , 1.14]
Combined 0.96 [0.88 , 1.05]
Heterogeneity P−value= 0.0167
Combined 0.90 [0.86 , 0.95]
Heterogeneity P−value= 0.0577
Hypertension
No History
NCI 1 0.92 [0.79 , 1.07]
NCI 2 0.92 [0.81 , 1.04]
MDA 0.90 [0.71 , 1.15]
IARC GWAS 0.89 [0.78 , 1.02]
IARC Replication 0.69 [0.57 , 0.84]
Combined 0.88 [0.82 , 0.94]
Heterogeneity P−value= 0.1571
History
NCI 1 0.94 [0.79 , 1.14]
NCI 2 0.92 [0.78 , 1.08]
MDA 0.65 [0.50 , 0.85]
IARC GWAS 0.88 [0.77 , 1.00]
IARC Replication 1.02 [0.82 , 1.26]
Combined 0.90 [0.83 , 0.97]
Heterogeneity P−value= 0.1248
Overall 0.88 [0.84 , 0.93]
Heterogeneity P−value= 0.6883
Sex
Male
NCI 1 0.98 [0.85 , 1.12]
NCI 2 0.85 [0.76 , 0.94]
MDA 0.72 [0.58 , 0.89]
IARC GWAS 0.91 [0.84 , 0.98]
IARC Replication 0.79 [0.69 , 0.89]
Combined 0.87 [0.83 , 0.92]
Heterogeneity P−value= 0.0432
Female
NCI 1 0.86 [0.70 , 1.06]
NCI 2 1.03 [0.89 , 1.19]
MDA 0.95 [0.69 , 1.32]
IARC GWAS 0.90 [0.82 , 0.99]
IARC Replication 0.97 [0.83 , 1.13]
Combined 0.94 [0.88 , 1.00]
Heterogeneity P−value= 0.5489
Overall 0.90 [0.86 , 0.93]
Heterogeneity P−value= 0.0866
Smoking
Never
NCI 1 0.86 [0.68 , 1.07]
NCI 2 0.87 [0.73 , 1.04]
MDA 0.83 [0.64 , 1.07]
IARC GWAS 0.92 [0.84 , 1.02]
IARC Replication 0.95 [0.82 , 1.09]
Combined 0.91 [0.85 , 0.97]
Heterogeneity P−value= 0.8282
Former
NCI 1 1.03 [0.85 , 1.25]
NCI 2 0.94 [0.76 , 1.15]
MDA 0.75 [0.56 , 1.00]
IARC GWAS 0.88 [0.77 , 1.00]
IARC Replication 1.02 [0.82 , 1.26]
Combined 0.93 [0.85 , 1.01]
Heterogeneity P−value= 0.3279
Current
NCI 1 0.92 [0.75 , 1.13]
NCI 2 0.87 [0.62 , 1.22]
MDA 0.64 [0.39 , 1.04]
IARC GWAS 0.89 [0.78 , 1.02]
IARC Replication 0.69 [0.57 , 0.84]
Combined 0.83 [0.76 , 0.91]
Heterogeneity P−value= 0.1729
Overall 0.89 [0.85 , 0.94]
Heterogeneity P−value= 0.2242
0.00
0.50
1.00
1.50
2.00
Odds Ratio

rs234043

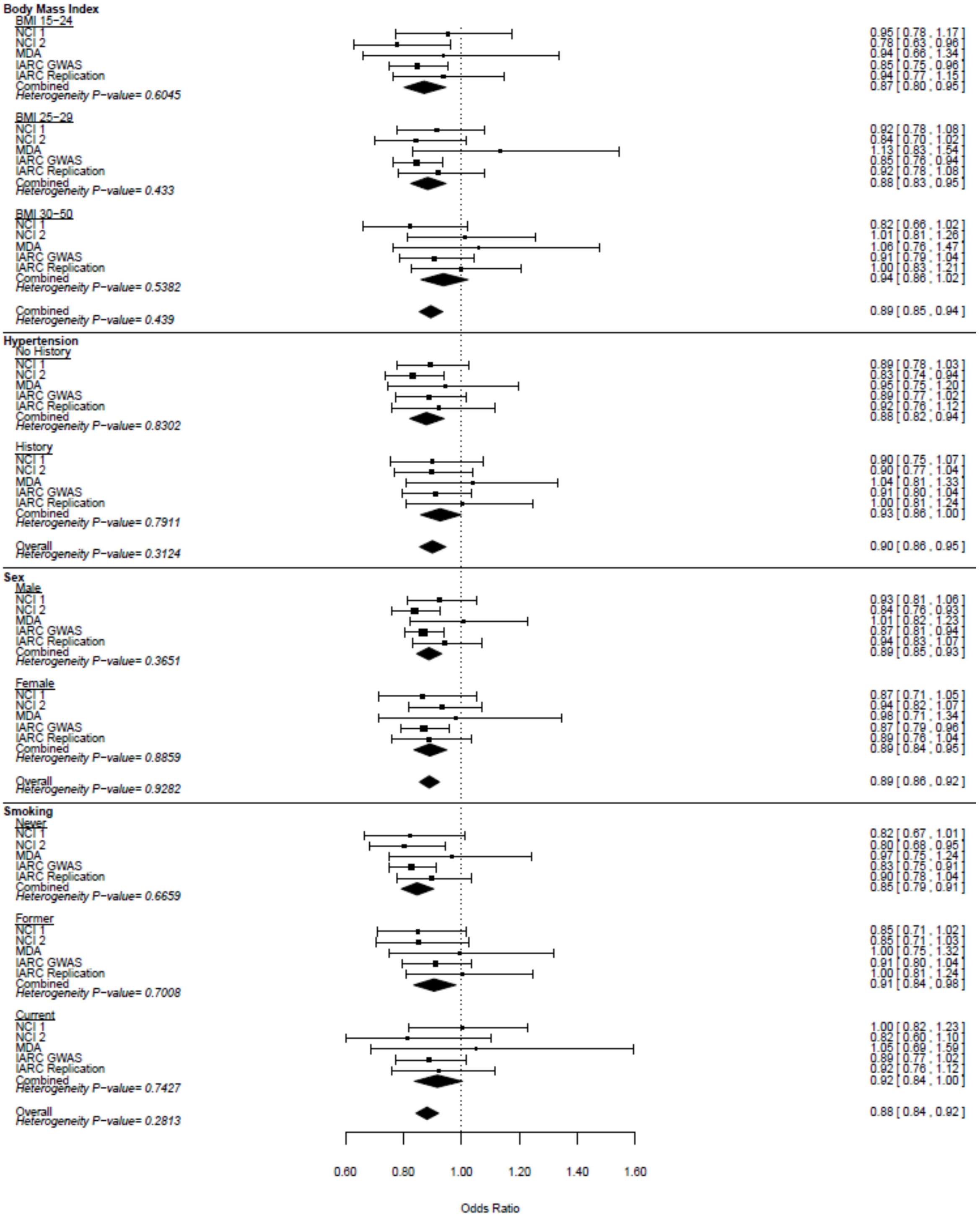
Body Mass Index
BMI 15−24
NCI 1 0.95 [0.78 , 1.17]
NCI 2 0.78 [0.63 , 0.96]
MDA 0.94 [0.66 , 1.34]
IARC GWAS 0.85 [0.75 , 0.96]
IARC Replication 0.94 [0.77 , 1.15]
Combined 0.87 [0.80 , 0.95]
Heterogeneity P−value= 0.6045
BMI 25−29
NCI 1 0.92 [0.78 , 1.08]
NCI 2 0.84 [0.70 , 1.02]
MDA 1.13 [0.83 , 1.54]
IARC GWAS 0.85 [0.76 , 0.94]
IARC Replication 0.92 [0.78 , 1.08]
Combined 0.88 [0.83 , 0.95]
Heterogeneity P−value= 0.433
BMI 30−50
NCI 1 0.82 [0.66 , 1.02]
NCI 2 1.01 [0.81 , 1.26]
MDA 1.06 [0.76 , 1.47]
IARC GWAS 0.91 [0.79 , 1.04]
IARC Replication 1.00 [0.83 , 1.21]
Combined 0.94 [0.86 , 1.02]
Heterogeneity P−value= 0.5382
Combined 0.89 [0.85 , 0.94]
Heterogeneity P−value= 0.439
Hypertension
No History
NCI 1 0.89 [0.78 , 1.03]
NCI 2 0.83 [0.74 , 0.94]
MDA 0.95 [0.75 , 1.20]
IARC GWAS 0.89 [0.77 , 1.02]
IARC Replication 0.92 [0.76 , 1.12]
Combined 0.88 [0.82 , 0.94]
Heterogeneity P−value= 0.8302
History
NCI 1 0.90 [0.75 , 1.07]
NCI 2 0.90 [0.77 , 1.04]
MDA 1.04 [0.81 , 1.33]
IARC GWAS 0.91 [0.80 , 1.04]
IARC Replication 1.00 [0.81 , 1.24]
Combined 0.93 [0.86 , 1.00]
Heterogeneity P−value= 0.7911
Overall 0.90 [0.86 , 0.95]
Heterogeneity P−value= 0.3124
Sex
Male
NCI 1 0.93 [0.81 , 1.06]
NCI 2 0.84 [0.76 , 0.93]
MDA 1.01 [0.82 , 1.23]
IARC GWAS 0.87 [0.81 , 0.94]
IARC Replication 0.94 [0.83 , 1.07]
Combined 0.89 [0.85 , 0.93]
Heterogeneity P−value= 0.3651
Female
NCI 1 0.87 [0.71 , 1.05]
NCI 2 0.94 [0.82 , 1.07]
MDA 0.98 [0.71 , 1.34]
IARC GWAS 0.87 [0.79 , 0.96]
IARC Replication 0.89 [0.76 , 1.04]
Combined 0.89 [0.84 , 0.95]
Heterogeneity P−value= 0.8859
Overall 0.89 [0.86 , 0.92]
Heterogeneity P−value= 0.9282
Smoking
Never
NCI 1 0.82 [0.67 , 1.01]
NCI 2 0.80 [0.68 , 0.95]
MDA 0.97 [0.75 , 1.24]
IARC GWAS 0.83 [0.75 , 0.91]
IARC Replication 0.90 [0.78 , 1.04]
Combined 0.85 [0.79 , 0.91]
Heterogeneity P−value= 0.6659
Former
NCI 1 0.85 [0.71 , 1.02]
NCI 2 0.85 [0.71 , 1.03]
MDA 1.00 [0.75 , 1.32]
IARC GWAS 0.91 [0.80 , 1.04]
IARC Replication 1.00 [0.81 , 1.24]
Combined 0.91 [0.84 , 0.98]
Heterogeneity P−value= 0.7008
Current
NCI 1 1.00 [0.82 , 1.23]
NCI 2 0.82 [0.60 , 1.10]
MDA 1.05 [0.69 , 1.59]
IARC GWAS 0.89 [0.77 , 1.02]
IARC Replication 0.92 [0.76 , 1.12]
Combined 0.92 [0.84 , 1.00]
Heterogeneity P−value= 0.7427
Overall 0.88 [0.84 , 0.92]
Heterogeneity P−value= 0.2813
0.60
0.80
1.00
1.20
1.40
1.60
Odds Ratio

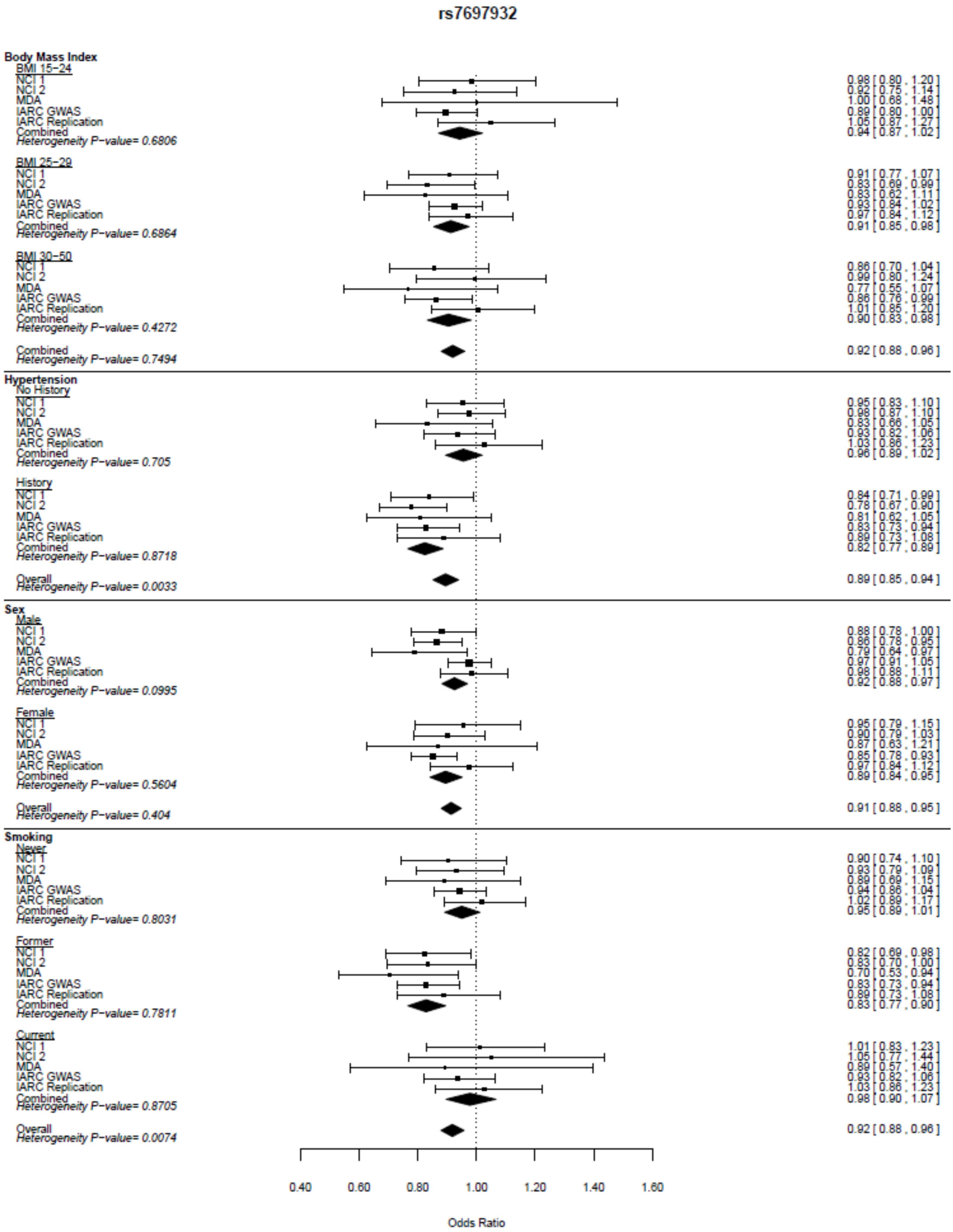
rs7697932
Body Mass Index
BMI 15−24
NCI 1 0.98 [0.80 , 1.20]
NCI 2 0.92 [0.75 , 1.14]
MDA 1.00 [0.68 , 1.48]
IARC GWAS 0.89 [0.80 , 1.00]
IARC Replication 1.05 [0.87 , 1.27]
Combined 0.94 [0.87 , 1.02]
Heterogeneity P−value= 0.6806
BMI 25−29
NCI 1 0.91 [0.77 , 1.07]
NCI 2 0.83 [0.69 , 0.99]
MDA 0.83 [0.62 , 1.11]
IARC GWAS 0.93 [0.84 , 1.02]
IARC Replication 0.97 [0.84 , 1.12]
Combined 0.91 [0.85 , 0.98]
Heterogeneity P−value= 0.6864
BMI 30−50
NCI 1 0.86 [0.70 , 1.04]
NCI 2 0.99 [0.80 , 1.24]
MDA 0.77 [0.55 , 1.07]
IARC GWAS 0.86 [0.76 , 0.99]
IARC Replication 1.01 [0.85 , 1.20]
Combined 0.90 [0.83 , 0.98]
Heterogeneity P−value= 0.4272
Combined 0.92 [0.88 , 0.96]
Heterogeneity P−value= 0.7494
Hypertension
No History
NCI 1 0.95 [0.83 , 1.10]
NCI 2 0.98 [0.87 , 1.10]
MDA 0.83 [0.66 , 1.05]
IARC GWAS 0.93 [0.82 , 1.06]
IARC Replication 1.03 [0.86 , 1.23]
Combined 0.96 [0.89 , 1.02]
Heterogeneity P−value= 0.705
History
NCI 1 0.84 [0.71 , 0.99]
NCI 2 0.78 [0.67 , 0.90]
MDA 0.81 [0.62 , 1.05]
IARC GWAS 0.83 [0.73 , 0.94]
IARC Replication 0.89 [0.73 , 1.08]
Combined 0.82 [0.77 , 0.89]
Heterogeneity P−value= 0.8718
Overall 0.89 [0.85 , 0.94]
Heterogeneity P−value= 0.0033
Sex
Male
NCI 1 0.88 [0.78 , 1.00]
NCI 2 0.86 [0.78 , 0.95]
MDA 0.79 [0.64 , 0.97]
IARC GWAS 0.97 [0.91 , 1.05]
IARC Replication 0.98 [0.88 , 1.11]
Combined 0.92 [0.88 , 0.97]
Heterogeneity P−value= 0.0995
Female
NCI 1 0.95 [0.79 , 1.15]
NCI 2 0.90 [0.79 , 1.03]
MDA 0.87 [0.63 , 1.21]
IARC GWAS 0.85 [0.78 , 0.93]
IARC Replication 0.97 [0.84 , 1.12]
Combined 0.89 [0.84 , 0.95]
Heterogeneity P−value= 0.5604
Overall 0.91 [0.88 , 0.95]
Heterogeneity P−value= 0.404
Smoking
Never
NCI 1 0.90 [0.74 , 1.10]
NCI 2 0.93 [0.79 , 1.09]
MDA 0.89 [0.69 , 1.15]
IARC GWAS 0.94 [0.86 , 1.04]
IARC Replication 1.02 [0.89 , 1.17]
Combined 0.95 [0.89 , 1.01]
Heterogeneity P−value= 0.8031
Former
NCI 1 0.82 [0.69 , 0.98]
NCI 2 0.83 [0.70 , 1.00]
MDA 0.70 [0.53 , 0.94]
IARC GWAS 0.83 [0.73 , 0.94]
IARC Replication 0.89 [0.73 , 1.08]
Combined 0.83 [0.77 , 0.90]
Heterogeneity P−value= 0.7811
Current
NCI 1 1.01 [0.83 , 1.23]
NCI 2 1.05 [0.77 , 1.44]
MDA 0.89 [0.57 , 1.40]
IARC GWAS 0.93 [0.82 , 1.06]
IARC Replication 1.03 [0.86 , 1.23]
Combined 0.98 [0.90 , 1.07]
Heterogeneity P−value= 0.8705
Overall 0.92 [0.88 , 0.96]
Heterogeneity P−value= 0.0074
0.40 0.60 0.80 1.00 1.20 1.40 1.60
Odds Ratio

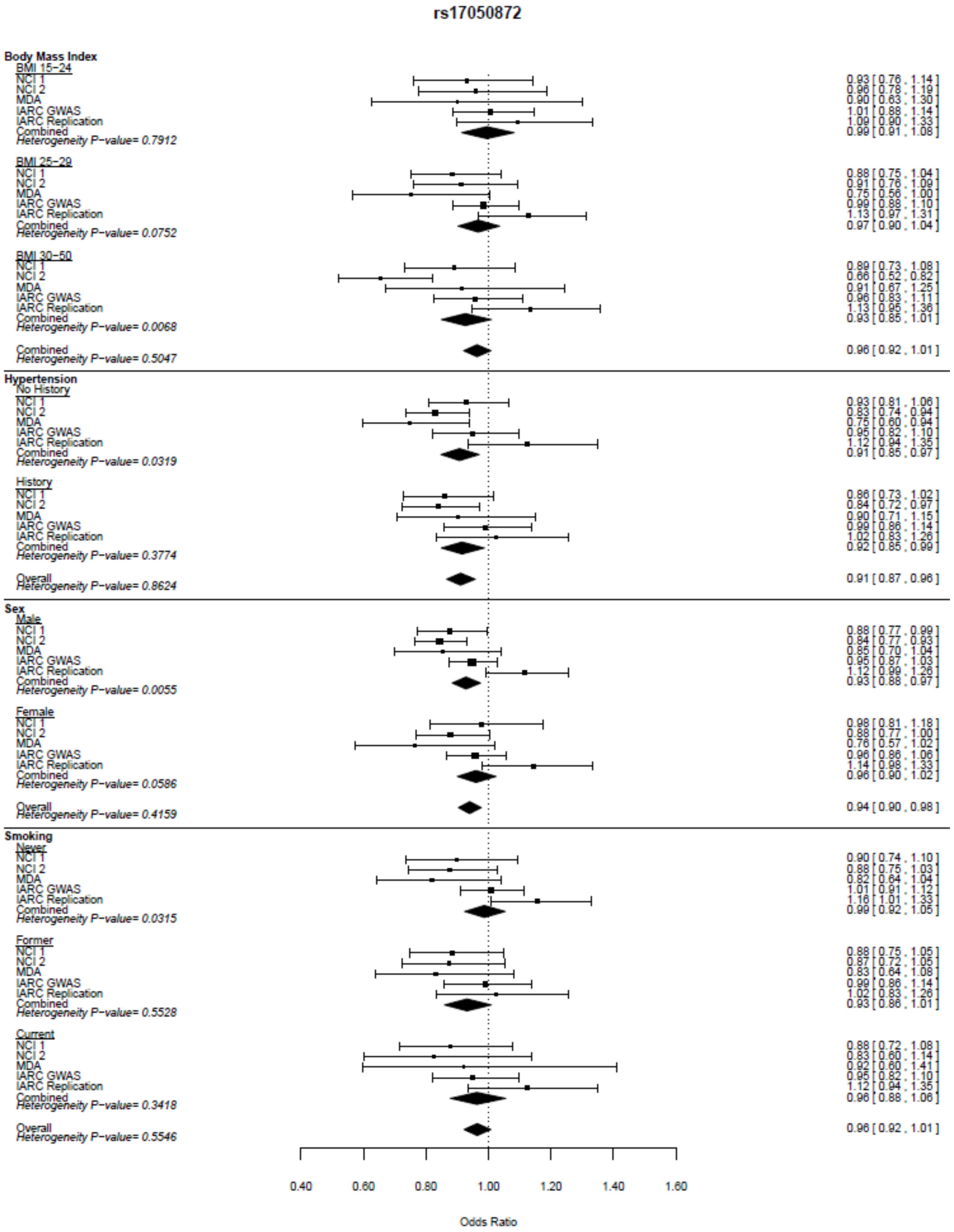
rs17050872
Body Mass Index
BMI 15−24
NCI 1 0.93 [0.76 , 1.14]
NCI 2 0.96 [0.78 , 1.19]
MDA 0.90 [0.63 , 1.30]
IARC GWAS 1.01 [0.88 , 1.14]
IARC Replication 1.09 [0.90 , 1.33]
Combined 0.99 [0.91 , 1.08]
Heterogeneity P−value= 0.7912
BMI 25−29
NCI 1 0.88 [0.75 , 1.04]
NCI 2 0.91 [0.76 , 1.09]
MDA 0.75 [0.56 , 1.00]
IARC GWAS 0.99 [0.88 , 1.10]
IARC Replication 1.13 [0.97 , 1.31]
Combined 0.97 [0.90 , 1.04]
Heterogeneity P−value= 0.0752
BMI 30−50
NCI 1 0.89 [0.73 , 1.08]
NCI 2 0.66 [0.52 , 0.82]
MDA 0.91 [0.67 , 1.25]
IARC GWAS 0.96 [0.83 , 1.11]
IARC Replication 1.13 [0.95 , 1.36]
Combined 0.93 [0.85 , 1.01]
Heterogeneity P−value= 0.0068
Combined 0.96 [0.92 , 1.01]
Heterogeneity P−value= 0.5047
Hypertension
No History
NCI 1 0.93 [0.81 , 1.06]
NCI 2 0.83 [0.74 , 0.94]
MDA 0.75 [0.60 , 0.94]
IARC GWAS 0.95 [0.82 , 1.10]
IARC Replication 1.12 [0.94 , 1.35]
Combined 0.91 [0.85 , 0.97]
Heterogeneity P−value= 0.0319
History
NCI 1 0.86 [0.73 , 1.02]
NCI 2 0.84 [0.72 , 0.97]
MDA 0.90 [0.71 , 1.15]
IARC GWAS 0.99 [0.86 , 1.14]
IARC Replication 1.02 [0.83 , 1.26]
Combined 0.92 [0.85 , 0.99]
Heterogeneity P−value= 0.3774
Overall 0.91 [0.87 , 0.96]
Heterogeneity P−value= 0.8624
Sex
Male
NCI 1 0.88 [0.77 , 0.99]
NCI 2 0.84 [0.77 , 0.93]
MDA 0.85 [0.70 , 1.04]
IARC GWAS 0.95 [0.87 , 1.03]
IARC Replication 1.12 [0.99 , 1.26]
Combined 0.93 [0.88 , 0.97]
Heterogeneity P−value= 0.0055
Female
NCI 1 0.98 [0.81 , 1.18]
NCI 2 0.88 [0.77 , 1.00]
MDA 0.76 [0.57 , 1.02]
IARC GWAS 0.96 [0.86 , 1.06]
IARC Replication 1.14 [0.98 , 1.33]
Combined 0.96 [0.90 , 1.02]
Heterogeneity P−value= 0.0586
Overall 0.94 [0.90 , 0.98]
Heterogeneity P−value= 0.4159
Smoking
Never
NCI 1 0.90 [0.74 , 1.10]
NCI 2 0.88 [0.75 , 1.03]
MDA 0.82 [0.64 , 1.04]
IARC GWAS 1.01 [0.91 , 1.12]
IARC Replication 1.16 [1.01 , 1.33]
Combined 0.99 [0.92 , 1.05]
Heterogeneity P−value= 0.0315
Former
NCI 1 0.88 [0.75 , 1.05]
NCI 2 0.87 [0.72 , 1.05]
MDA 0.83 [0.64 , 1.08]
IARC GWAS 0.99 [0.86 , 1.14]
IARC Replication 1.02 [0.83 , 1.26]
Combined 0.93 [0.86 , 1.01]
Heterogeneity P−value= 0.5528
Current
NCI 1 0.88 [0.72 , 1.08]
NCI 2 0.83 [0.60 , 1.14]
MDA 0.92 [0.60 , 1.41]
IARC GWAS 0.95 [0.82 , 1.10]
IARC Replication 1.12 [0.94 , 1.35]
Combined 0.96 [0.88 , 1.06]
Heterogeneity P−value= 0.3418
Overall 0.96 [0.92 , 1.01]
Heterogeneity P−value= 0.5546
0.40 0.60 0.80 1.00 1.20 1.40 1.60
Odds Ratio

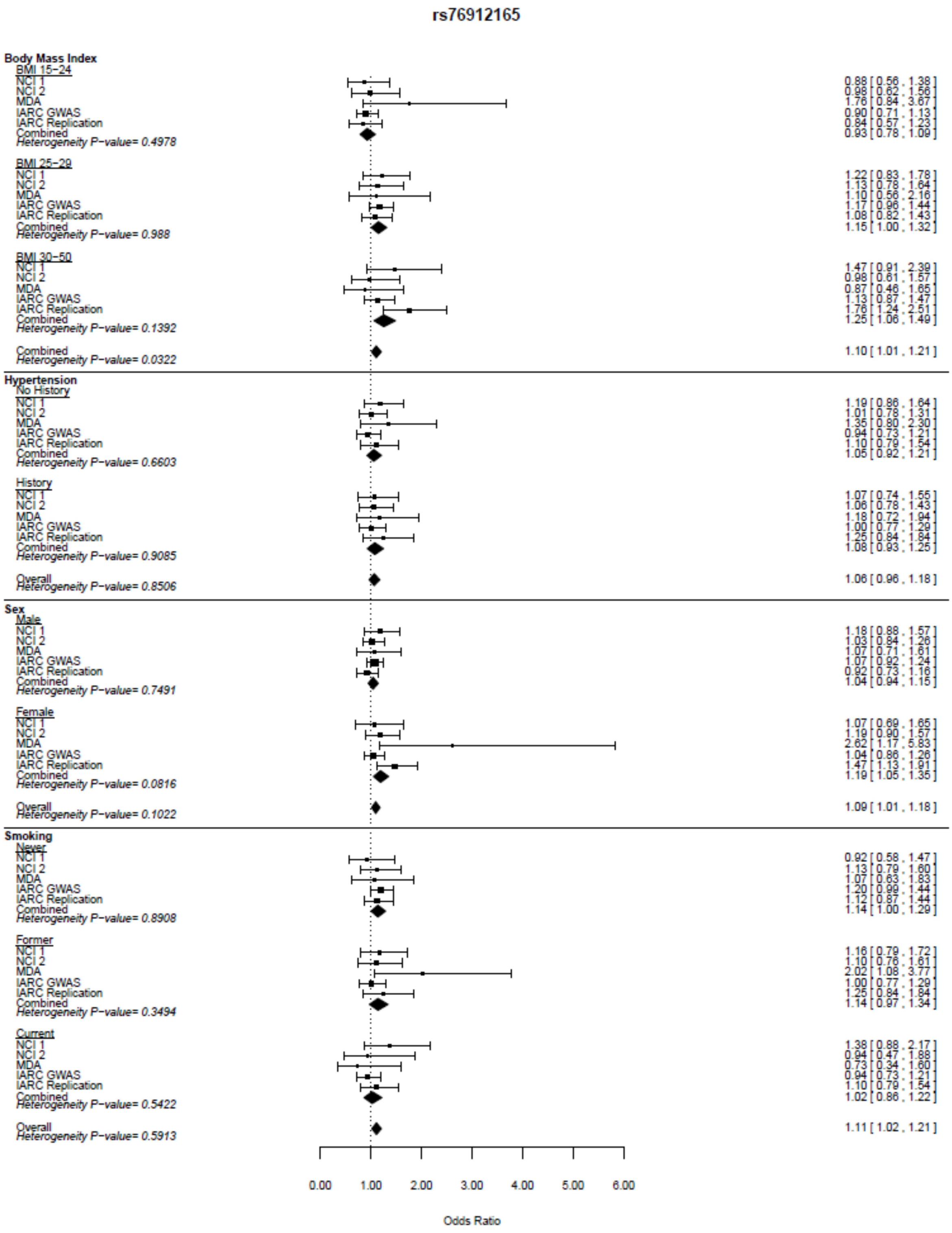
rs76912165
Body Mass Index
BMI 15-24
NCI 1
NCI 2
MDA
IARC GWAS
IARC Replication
Combined
Heterogeneity P-value= 0.4978
BMI 25-29
NCI 1
NCI 2
MDA
IARC GWAS
IARC Replication
Combined
Heterogeneity P-value= 0.988
BMI 30-50
NCI 1
NCI 2
MDA
IARC GWAS
IARC Replication
Combined
Heterogeneity P-value= 0.1392
Combined
Heterogeneity P-value= 0.0322
Hypertension
No History
NCI 1
NCI 2
MDA
IARC GWAS
IARC Replication
Combined
Heterogeneity P-value= 0.6603
History
NCI 1
NCI 2
MDA
IARC GWAS
IARC Replication
Combined
Heterogeneity P-value= 0.9085
Overall
Heterogeneity P-value= 0.8506
Sex
Male
NCI 1
NCI 2
MDA
IARC GWAS
IARC Replication
Combined
Heterogeneity P-value= 0.7491
Female
NCI 1
NCI 2
MDA
IARC GWAS
IARC Replication
Combined
Heterogeneity P-value= 0.0816
Overall
Heterogeneity P-value= 0.1022
Smoking
Never
NCI 1
NCI 2
MDA
IARC GWAS
IARC Replication
Combined
Heterogeneity P-value= 0.8908
Former
NCI 1
NCI 2
MDA
IARC GWAS
IARC Replication
Combined
Heterogeneity P-value= 0.3494
Current
NCI 1
NCI 2
MDA
IARC GWAS
IARC Replication
Combined
Heterogeneity P-value= 0.5422
Overall
Heterogeneity P-value= 0.5913
0.88 [0.56 , 1.38]
0.98 [0.62 , 1.56]
1.76 [0.84 , 3.67]
0.90 [0.71 , 1.13]
0.84 [0.57 , 1.23]
0.93 [0.78 , 1.09]
1.22 [0.83 , 1.78]
1.13 [0.78 , 1.64]
1.10 [0.56 , 2.16]
1.17 [0.96 , 1.44]
1.08 [0.82 , 1.43]
1.15 [1.00 , 1.32]
1.47 [0.91 , 2.39]
0.98 [0.61 , 1.57]
0.87 [0.46 , 1.65]
1.13 [0.87 , 1.47]
1.76 [1.24 , 2.51]
1.25 [1.06 , 1.49]
1.10 [1.01 , 1.21]
1.19 [0.86 , 1.64]
1.01 [0.78 , 1.31]
1.35 [0.80 , 2.30]
0.94 [0.73 , 1.21]
1.10 [0.79 , 1.54]
1.05 [0.92 , 1.21]
1.07 [0.74 , 1.55]
1.06 [0.78 , 1.43]
1.18 [0.72 , 1.94]
1.00 [0.77 , 1.29]
1.25 [0.84 , 1.84]
1.08 [0.93 , 1.25]
1.06 [0.96 , 1.18]
1.18 [0.88 , 1.57]
1.03 [0.84 , 1.26]
1.07 [0.71 , 1.61]
1.07 [0.92 , 1.24]
0.92 [0.73 , 1.16]
1.04 [0.94 , 1.15]
1.07 [0.69 , 1.65]
1.19 [0.90 , 1.57]
2.62 [1.17 , 5.83]
1.04 [0.86 , 1.26]
1.47 [1.13 , 1.91]
1.19 [1.05 , 1.35]
1.09 [1.01 , 1.18]
0.92 [0.58 , 1.47]
1.13 [0.79 , 1.60]
1.07 [0.63 , 1.83]
1.20 [0.99 , 1.44]
1.12 [0.87 , 1.44]
1.14 [1.00 , 1.29]
1.16 [0.79 , 1.72]
1.10 [0.76 , 1.61]
2.02 [1.08 , 3.77]
1.00 [0.77 , 1.29]
1.25 [0.84 , 1.84]
1.14 [0.97 , 1.34]
1.38 [0.88 , 2.17]
0.94 [0.47 , 1.88]
0.73 [0.34 , 1.60]
0.94 [0.73 , 1.21]
1.10 [0.79 , 1.54]
1.02 [0.86 , 1.22]
1.11 [1.02 , 1.21]
0.00
1.00
2.00
3.00
4.00
5.00
6.00
Odds Ratio

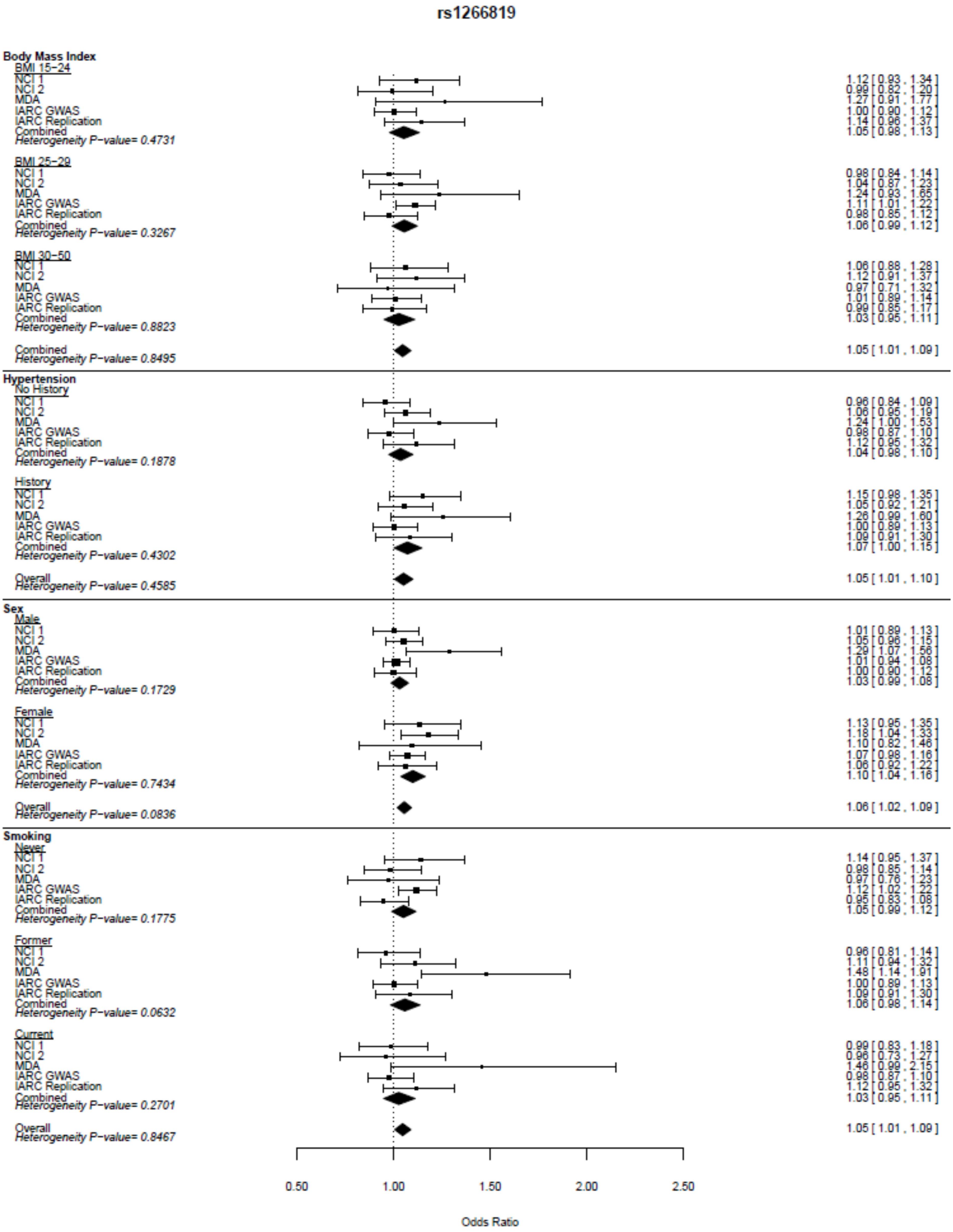
rs1266819
Body Mass Index
BMI 15−24
NCI 1 1.12 [0.93 , 1.34]
NCI 2 0.99 [0.82 , 1.20]
MDA 1.27 [0.91 , 1.77]
IARC GWAS 1.00 [0.90 , 1.12]
IARC Replication 1.14 [0.96 , 1.37]
Combined 1.05 [0.98 , 1.13]
Heterogeneity P−value= 0.4731
BMI 25−29
NCI 1 0.98 [0.84 , 1.14]
NCI 2 1.04 [0.87 , 1.23]
MDA 1.24 [0.93 , 1.65]
IARC GWAS 1.11 [1.01 , 1.22]
IARC Replication 0.98 [0.85 , 1.12]
Combined 1.06 [0.99 , 1.12]
Heterogeneity P−value= 0.3267
BMI 30−50
NCI 1 1.06 [0.88 , 1.28]
NCI 2 1.12 [0.91 , 1.37]
MDA 0.97 [0.71 , 1.32]
IARC GWAS 1.01 [0.89 , 1.14]
IARC Replication 0.99 [0.85 , 1.17]
Combined 1.03 [0.95 , 1.11]
Heterogeneity P−value= 0.8823
Combined 1.05 [1.01 , 1.09]
Heterogeneity P−value= 0.8495
Hypertension
No History
NCI 1 0.96 [0.84 , 1.09]
NCI 2 1.06 [0.95 , 1.19]
MDA 1.24 [1.00 , 1.53]
IARC GWAS 0.98 [0.87 , 1.10]
IARC Replication 1.12 [0.95 , 1.32]
Combined 1.04 [0.98 , 1.10]
Heterogeneity P−value= 0.1878
History
NCI 1 1.15 [0.98 , 1.35]
NCI 2 1.05 [0.92 , 1.21]
MDA 1.26 [0.99 , 1.60]
IARC GWAS 1.00 [0.89 , 1.13]
IARC Replication 1.09 [0.91 , 1.30]
Combined 1.07 [1.00 , 1.15]
Heterogeneity P−value= 0.4302
Overall 1.05 [1.01 , 1.10]
Heterogeneity P−value= 0.4585
Sex
Male
NCI 1 1.01 [0.89 , 1.13]
NCI 2 1.05 [0.96 , 1.15]
MDA 1.29 [1.07 , 1.56]
IARC GWAS 1.01 [0.94 , 1.08]
IARC Replication 1.00 [0.90 , 1.12]
Combined 1.03 [0.99 , 1.08]
Heterogeneity P−value= 0.1729
Female
NCI 1 1.13 [0.95 , 1.35]
NCI 2 1.18 [1.04 , 1.33]
MDA 1.10 [0.82 , 1.46]
IARC GWAS 1.07 [0.98 , 1.16]
IARC Replication 1.06 [0.92 , 1.22]
Combined 1.10 [1.04 , 1.16]
Heterogeneity P−value= 0.7434
Overall 1.06 [1.02 , 1.09]
Heterogeneity P−value= 0.0836
Smoking
Never
NCI 1 1.14 [0.95 , 1.37]
NCI 2 0.98 [0.85 , 1.14]
MDA 0.97 [0.76 , 1.23]
IARC GWAS 1.12 [1.02 , 1.22]
IARC Replication 0.95 [0.83 , 1.08]
Combined 1.05 [0.99 , 1.12]
Heterogeneity P−value= 0.1775
Former
NCI 1 0.96 [0.81 , 1.14]
NCI 2 1.11 [0.94 , 1.32]
MDA 1.48 [1.14 , 1.91]
IARC GWAS 1.00 [0.89 , 1.13]
IARC Replication 1.09 [0.91 , 1.30]
Combined 1.06 [0.98 , 1.14]
Heterogeneity P−value= 0.0632
Current
NCI 1 0.99 [0.83 , 1.18]
NCI 2 0.96 [0.73 , 1.27]
MDA 1.46 [0.99 , 2.15]
IARC GWAS 0.98 [0.87 , 1.10]
IARC Replication 1.12 [0.95 , 1.32]
Combined 1.03 [0.95 , 1.11]
Heterogeneity P−value= 0.2701
Overall 1.05 [1.01 , 1.09]
Heterogeneity P−value= 0.8467
0.50
1.00
1.50
2.00
2.50
Odds Ratio

rs59294613

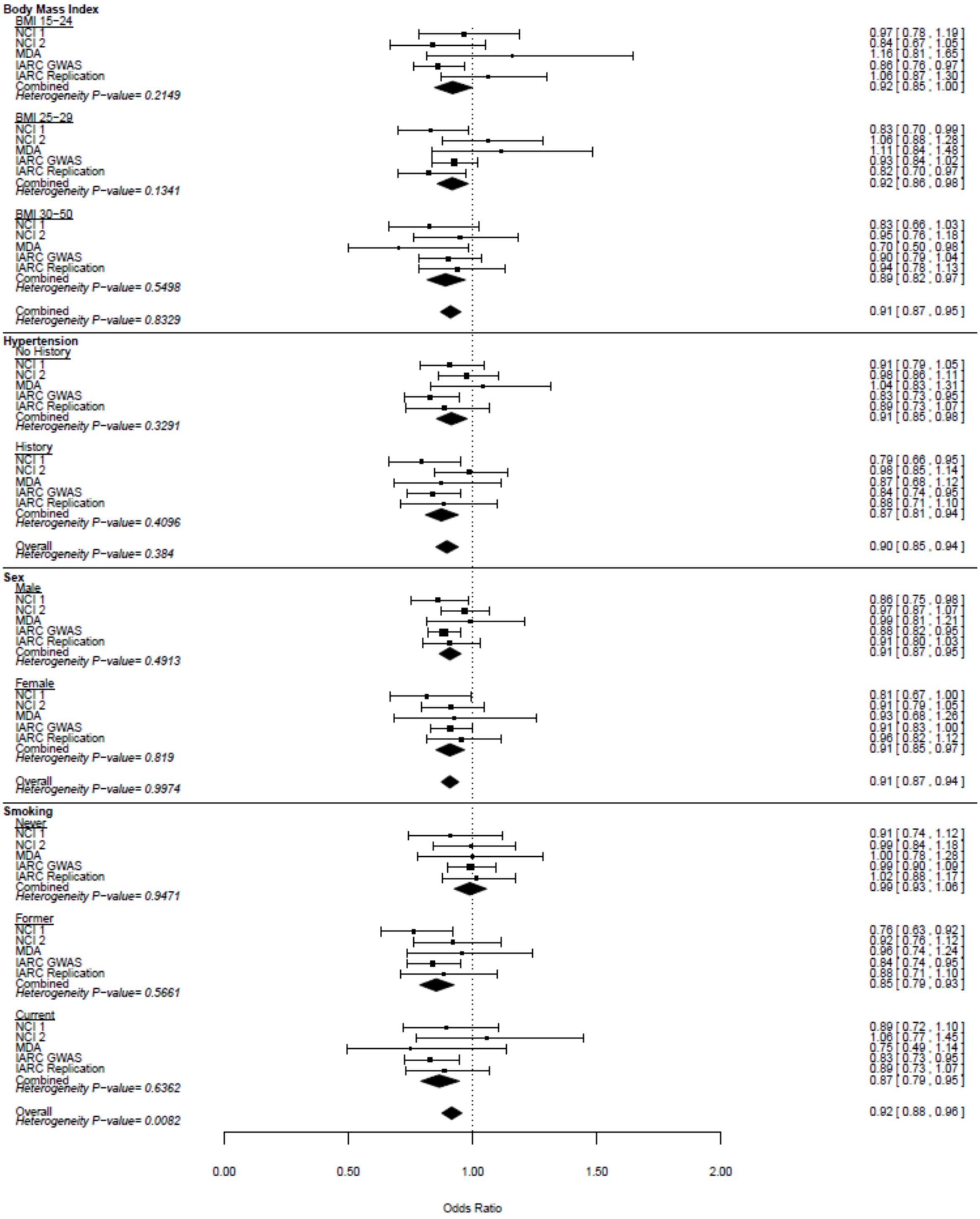
Body Mass Index
BMI 15−24
NCI 1 0.97 [0.78 , 1.19]
NCI 2 0.84 [0.67 , 1.05]
MDA 1.16 [0.81 , 1.65]
IARC GWAS 0.86 [0.76 , 0.97]
IARC Replication 1.06 [0.87 , 1.30]
Combined 0.92 [0.85 , 1.00]
Heterogeneity P−value= 0.2149
BMI 25−29
NCI 1 0.83 [0.70 , 0.99]
NCI 2 1.06 [0.88 , 1.28]
MDA 1.11 [0.84 , 1.48]
IARC GWAS 0.93 [0.84 , 1.02]
IARC Replication 0.82 [0.70 , 0.97]
Combined 0.92 [0.86 , 0.98]
Heterogeneity P−value= 0.1341
BMI 30−50
NCI 1 0.83 [0.66 , 1.03]
NCI 2 0.95 [0.76 , 1.18]
MDA 0.70 [0.50 , 0.98]
IARC GWAS 0.90 [0.79 , 1.04]
IARC Replication 0.94 [0.78 , 1.13]
Combined 0.89 [0.82 , 0.97]
Heterogeneity P−value= 0.5498
Combined 0.91 [0.87 , 0.95]
Heterogeneity P−value= 0.8329
Hypertension
No History
NCI 1 0.91 [0.79 , 1.05]
NCI 2 0.98 [0.86 , 1.11]
MDA 1.04 [0.83 , 1.31]
IARC GWAS 0.83 [0.73 , 0.95]
IARC Replication 0.89 [0.73 , 1.07]
Combined 0.91 [0.85 , 0.98]
Heterogeneity P−value= 0.3291
History
NCI 1 0.79 [0.66 , 0.95]
NCI 2 0.98 [0.85 , 1.14]
MDA 0.87 [0.68 , 1.12]
IARC GWAS 0.84 [0.74 , 0.95]
IARC Replication 0.88 [0.71 , 1.10]
Combined 0.87 [0.81 , 0.94]
Heterogeneity P−value= 0.4096
Overall 0.90 [0.85 , 0.94]
Heterogeneity P−value= 0.384
Sex
Male
NCI 1 0.86 [0.75 , 0.98]
NCI 2 0.97 [0.87 , 1.07]
MDA 0.99 [0.81 , 1.21]
IARC GWAS 0.88 [0.82 , 0.95]
IARC Replication 0.91 [0.80 , 1.03]
Combined 0.91 [0.87 , 0.95]
Heterogeneity P−value= 0.4913
Female
NCI 1 0.81 [0.67 , 1.00]
NCI 2 0.91 [0.79 , 1.05]
MDA 0.93 [0.68 , 1.26]
IARC GWAS 0.91 [0.83 , 1.00]
IARC Replication 0.96 [0.82 , 1.12]
Combined 0.91 [0.85 , 0.97]
Heterogeneity P−value= 0.819
Overall 0.91 [0.87 , 0.94]
Heterogeneity P−value= 0.9974
Smoking
Never
NCI 1 0.91 [0.74 , 1.12]
NCI 2 0.99 [0.84 , 1.18]
MDA 1.00 [0.78 , 1.28]
IARC GWAS 0.99 [0.90 , 1.09]
IARC Replication 1.02 [0.88 , 1.17]
Combined 0.99 [0.93 , 1.06]
Heterogeneity P−value= 0.9471
Former
NCI 1 0.76 [0.63 , 0.92]
NCI 2 0.92 [0.76 , 1.12]
MDA 0.96 [0.74 , 1.24]
IARC GWAS 0.84 [0.74 , 0.95]
IARC Replication 0.88 [0.71 , 1.10]
Combined 0.85 [0.79 , 0.93]
Heterogeneity P−value= 0.5661
Current
NCI 1 0.89 [0.72 , 1.10]
NCI 2 1.06 [0.77 , 1.45]
MDA 0.75 [0.49 , 1.14]
IARC GWAS 0.83 [0.73 , 0.95]
IARC Replication 0.89 [0.73 , 1.07]
Combined 0.87 [0.79 , 0.95]
Heterogeneity P−value= 0.6362
Overall 0.92 [0.88 , 0.96]
Heterogeneity P−value= 0.0082
0.00
0.50
1.00
1.50
2.00
Odds Ratio

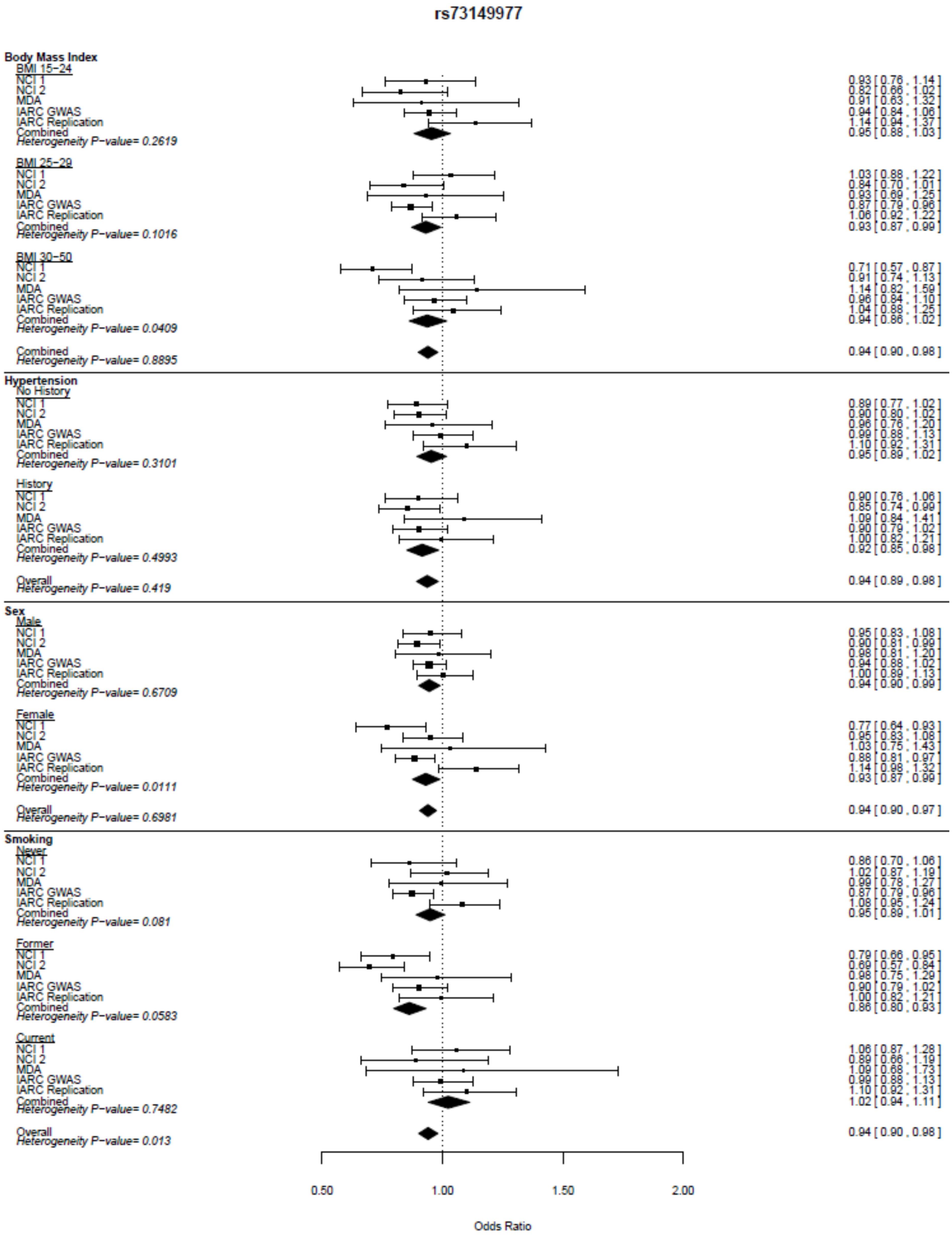
rs73149977
Body Mass Index
BMI 15−24
NCI 1
NCI 2
MDA
IARC GWAS
IARC Replication
Combined
Heterogeneity P−value= 0.2619
0.93 [0.76 , 1.14]
0.82 [0.66 , 1.02]
0.91 [0.63 , 1.32]
0.94 [0.84 , 1.06]
1.14 [0.94 , 1.37]
0.95 [0.88 , 1.03]
BMI 25−29
NCI 1
NCI 2
MDA
IARC GWAS
IARC Replication
Combined
Heterogeneity P−value= 0.1016
1.03 [0.88 , 1.22]
0.84 [0.70 , 1.01]
0.93 [0.69 , 1.25]
0.87 [0.79 , 0.96]
1.06 [0.92 , 1.22]
0.93 [0.87 , 0.99]
BMI 30−50
NCI 1
NCI 2
MDA
IARC GWAS
IARC Replication
Combined
Heterogeneity P−value= 0.0409
0.71 [0.57 , 0.87]
0.91 [0.74 , 1.13]
1.14 [0.82 , 1.59]
0.96 [0.84 , 1.10]
1.04 [0.88 , 1.25]
0.94 [0.86 , 1.02]
Combined
Heterogeneity P−value= 0.8895
0.94 [0.90 , 0.98]
Hypertension
No History
NCI 1
NCI 2
MDA
IARC GWAS
IARC Replication
Combined
Heterogeneity P−value= 0.3101
0.89 [0.77 , 1.02]
0.90 [0.80 , 1.02]
0.96 [0.76 , 1.20]
0.99 [0.88 , 1.13]
1.10 [0.92 , 1.31]
0.95 [0.89 , 1.02]
History
NCI 1
NCI 2
MDA
IARC GWAS
IARC Replication
Combined
Heterogeneity P−value= 0.4993
0.90 [0.76 , 1.06]
0.85 [0.74 , 0.99]
1.09 [0.84 , 1.41]
0.90 [0.79 , 1.02]
1.00 [0.82 , 1.21]
0.92 [0.85 , 0.98]
Overall
Heterogeneity P−value= 0.419
0.94 [0.89 , 0.98]
Sex
Male
NCI 1
NCI 2
MDA
IARC GWAS
IARC Replication
Combined
Heterogeneity P−value= 0.6709
0.95 [0.83 , 1.08]
0.90 [0.81 , 0.99]
0.98 [0.81 , 1.20]
0.94 [0.88 , 1.02]
1.00 [0.89 , 1.13]
0.94 [0.90 , 0.99]
Female
NCI 1
NCI 2
MDA
IARC GWAS
IARC Replication
Combined
Heterogeneity P−value= 0.0111
0.77 [0.64 , 0.93]
0.95 [0.83 , 1.08]
1.03 [0.75 , 1.43]
0.88 [0.81 , 0.97]
1.14 [0.98 , 1.32]
0.93 [0.87 , 0.99]
Overall
Heterogeneity P−value= 0.6981
0.94 [0.90 , 0.97]
Smoking
Never
NCI 1
NCI 2
MDA
IARC GWAS
IARC Replication
Combined
Heterogeneity P−value= 0.081
0.86 [0.70 , 1.06]
1.02 [0.87 , 1.19]
0.99 [0.78 , 1.27]
0.87 [0.79 , 0.96]
1.08 [0.95 , 1.24]
0.95 [0.89 , 1.01]
Former
NCI 1
NCI 2
MDA
IARC GWAS
IARC Replication
Combined
Heterogeneity P−value= 0.0583
0.79 [0.66 , 0.95]
0.69 [0.57 , 0.84]
0.98 [0.75 , 1.29]
0.90 [0.79 , 1.02]
1.00 [0.82 , 1.21]
0.86 [0.80 , 0.93]
Current
NCI 1
NCI 2
MDA
IARC GWAS
IARC Replication
Combined
Heterogeneity P−value= 0.7482
1.06 [0.87 , 1.28]
0.89 [0.66 , 1.19]
1.09 [0.68 , 1.73]
0.99 [0.88 , 1.13]
1.10 [0.92 , 1.31]
1.02 [0.94 , 1.11]
Overall
Heterogeneity P−value= 0.013
0.94 [0.90 , 0.98]
0.50
1.00
1.50
2.00
Odds Ratio

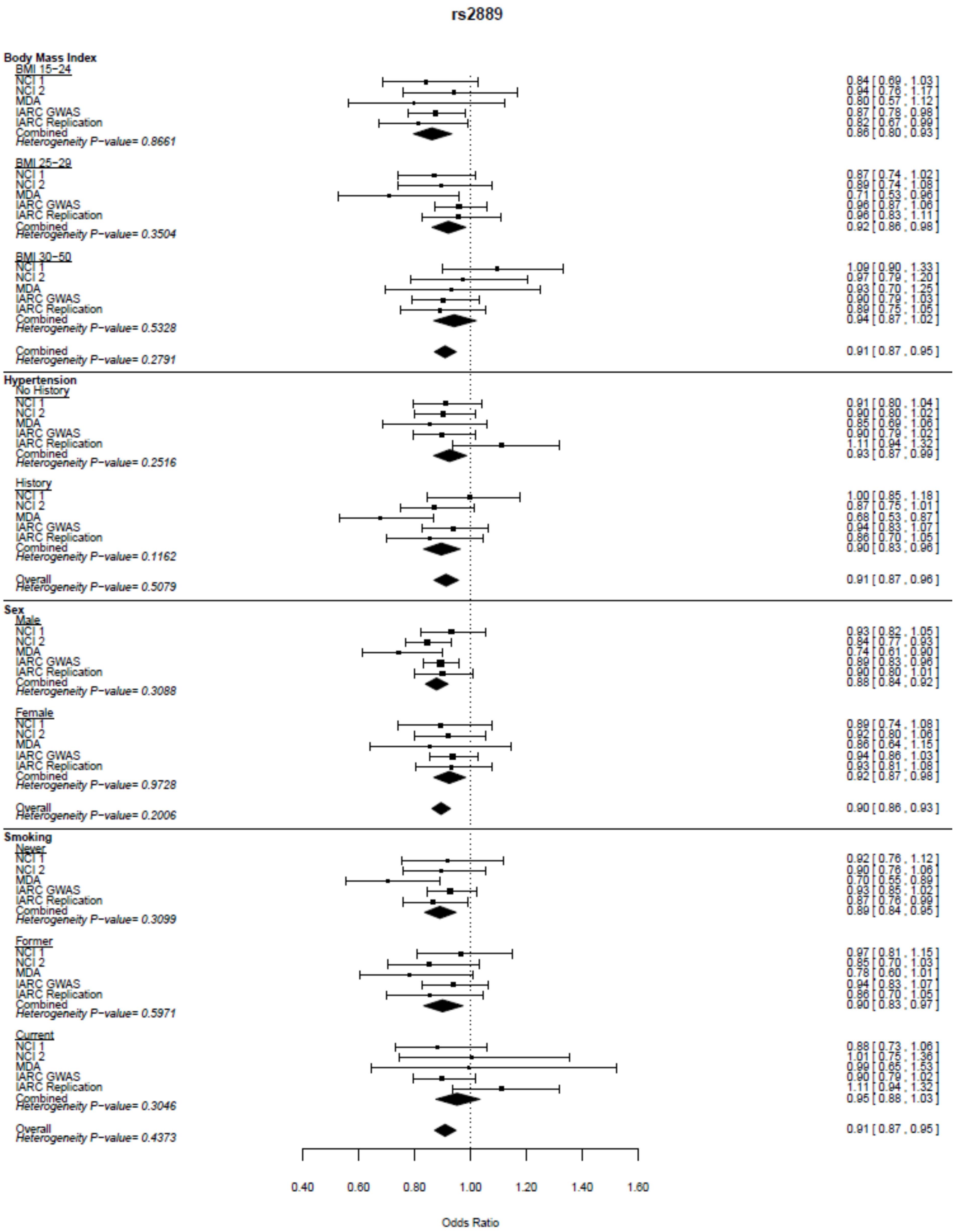
rs2889
Body Mass Index
BMI 15-24
NCI 1
NCI 2
MDA
IARC GWAS
IARC Replication
Combined
Heterogeneity P-value= 0.8661
0.84 [0.69 , 1.03]
0.94 [0.76 , 1.17]
0.80 [0.57 , 1.12]
0.87 [0.78 , 0.98]
0.82 [0.67 , 0.99]
0.86 [0.80 , 0.93]
BMI 25-29
NCI 1
NCI 2
MDA
IARC GWAS
IARC Replication
Combined
Heterogeneity P-value= 0.3504
0.87 [0.74 , 1.02]
0.89 [0.74 , 1.08]
0.71 [0.53 , 0.96]
0.96 [0.87 , 1.06]
0.96 [0.83 , 1.11]
0.92 [0.86 , 0.98]
BMI 30-50
NCI 1
NCI 2
MDA
IARC GWAS
IARC Replication
Combined
Heterogeneity P-value= 0.5328
1.09 [0.90 , 1.33]
0.97 [0.79 , 1.20]
0.93 [0.70 , 1.25]
0.90 [0.79 , 1.03]
0.89 [0.75 , 1.05]
0.94 [0.87 , 1.02]
Combined
Heterogeneity P-value= 0.2791
0.91 [0.87 , 0.95]
Hypertension
No History
NCI 1
NCI 2
MDA
IARC GWAS
IARC Replication
Combined
Heterogeneity P-value= 0.2516
0.91 [0.80 , 1.04]
0.90 [0.80 , 1.02]
0.85 [0.69 , 1.06]
0.90 [0.79 , 1.02]
1.11 [0.94 , 1.32]
0.93 [0.87 , 0.99]
History
NCI 1
NCI 2
MDA
IARC GWAS
IARC Replication
Combined
Heterogeneity P-value= 0.1162
1.00 [0.85 , 1.18]
0.87 [0.75 , 1.01]
0.68 [0.53 , 0.87]
0.94 [0.83 , 1.07]
0.86 [0.70 , 1.05]
0.90 [0.83 , 0.96]
Overall
Heterogeneity P-value= 0.5079
0.91 [0.87 , 0.96]
Sex
Male
NCI 1
NCI 2
MDA
IARC GWAS
IARC Replication
Combined
Heterogeneity P-value= 0.3088
0.93 [0.82 , 1.05]
0.84 [0.77 , 0.93]
0.74 [0.61 , 0.90]
0.89 [0.83 , 0.96]
0.90 [0.80 , 1.01]
0.88 [0.84 , 0.92]
Female
NCI 1
NCI 2
MDA
IARC GWAS
IARC Replication
Combined
Heterogeneity P-value= 0.9728
0.89 [0.74 , 1.08]
0.92 [0.80 , 1.06]
0.86 [0.64 , 1.15]
0.94 [0.86 , 1.03]
0.93 [0.81 , 1.08]
0.92 [0.87 , 0.98]
Overall
Heterogeneity P-value= 0.2006
0.90 [0.86 , 0.93]
Smoking
Never
NCI 1
NCI 2
MDA
IARC GWAS
IARC Replication
Combined
Heterogeneity P-value= 0.3099
0.92 [0.76 , 1.12]
0.90 [0.76 , 1.06]
0.70 [0.55 , 0.89]
0.93 [0.85 , 1.02]
0.87 [0.76 , 0.99]
0.89 [0.84 , 0.95]
Former
NCI 1
NCI 2
MDA
IARC GWAS
IARC Replication
Combined
Heterogeneity P-value= 0.5971
0.97 [0.81 , 1.15]
0.85 [0.70 , 1.03]
0.78 [0.60 , 1.01]
0.94 [0.83 , 1.07]
0.86 [0.70 , 1.05]
0.90 [0.83 , 0.97]
Current
NCI 1
NCI 2
MDA
IARC GWAS
IARC Replication
Combined
Heterogeneity P-value= 0.3046
0.88 [0.73 , 1.06]
1.01 [0.75 , 1.36]
0.99 [0.65 , 1.53]
0.90 [0.79 , 1.02]
1.11 [0.94 , 1.32]
0.95 [0.88 , 1.03]
Overall
Heterogeneity P-value= 0.4373
0.91 [0.87 , 0.95]
0.40
0.60
0.80
1.00
1.20
1.40
1.60
Odds Ratio

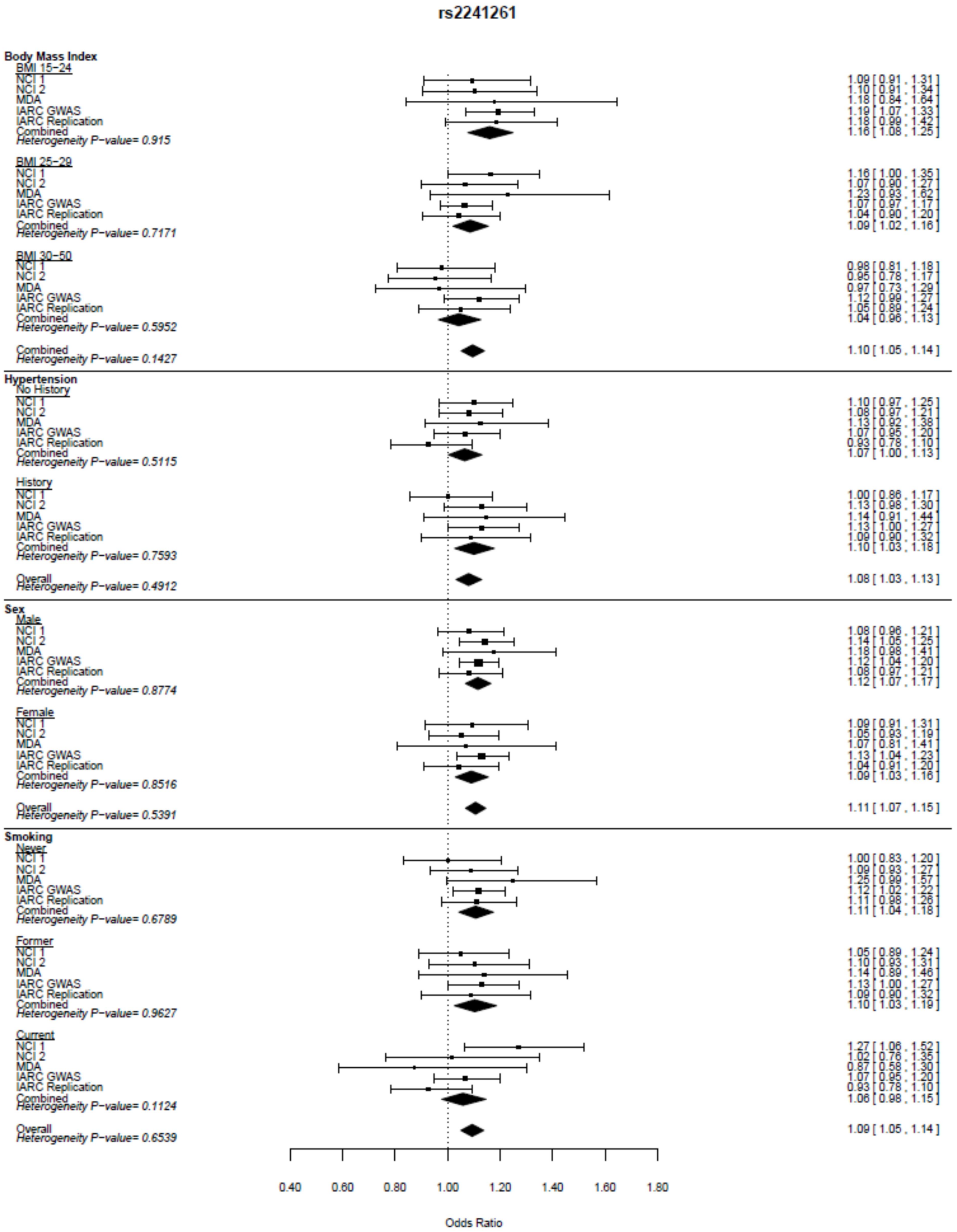
rs2241261
Body Mass Index
BMI 15−24
NCI 1
NCI 2
MDA
IARC GWAS
IARC Replication
Combined
Heterogeneity P−value= 0.915
BMI 25−29
Heterogeneity P−value= 0.7171
BMI 30−50
Heterogeneity P−value= 0.5952
Heterogeneity P−value= 0.1427
Hypertension
No History
Heterogeneity P−value= 0.5115
History
Heterogeneity P−value= 0.7593
Overall
Heterogeneity P−value= 0.4912
Sex
Male
Heterogeneity P−value= 0.8774
Female
Heterogeneity P−value= 0.8516
Heterogeneity P−value= 0.5391
Smoking
Never
Heterogeneity P−value= 0.6789
Former
Heterogeneity P−value= 0.9627
Current
Heterogeneity P−value= 0.1124
Heterogeneity P−value= 0.6539
1.10 [1.05 , 1.14]
1.08 [1.03 , 1.13]
1.11 [1.07 , 1.15]
1.09 [1.05 , 1.14]
0.40
0.60
0.80
1.00
1.20
1.40
1.60
1.80
Odds Ratio

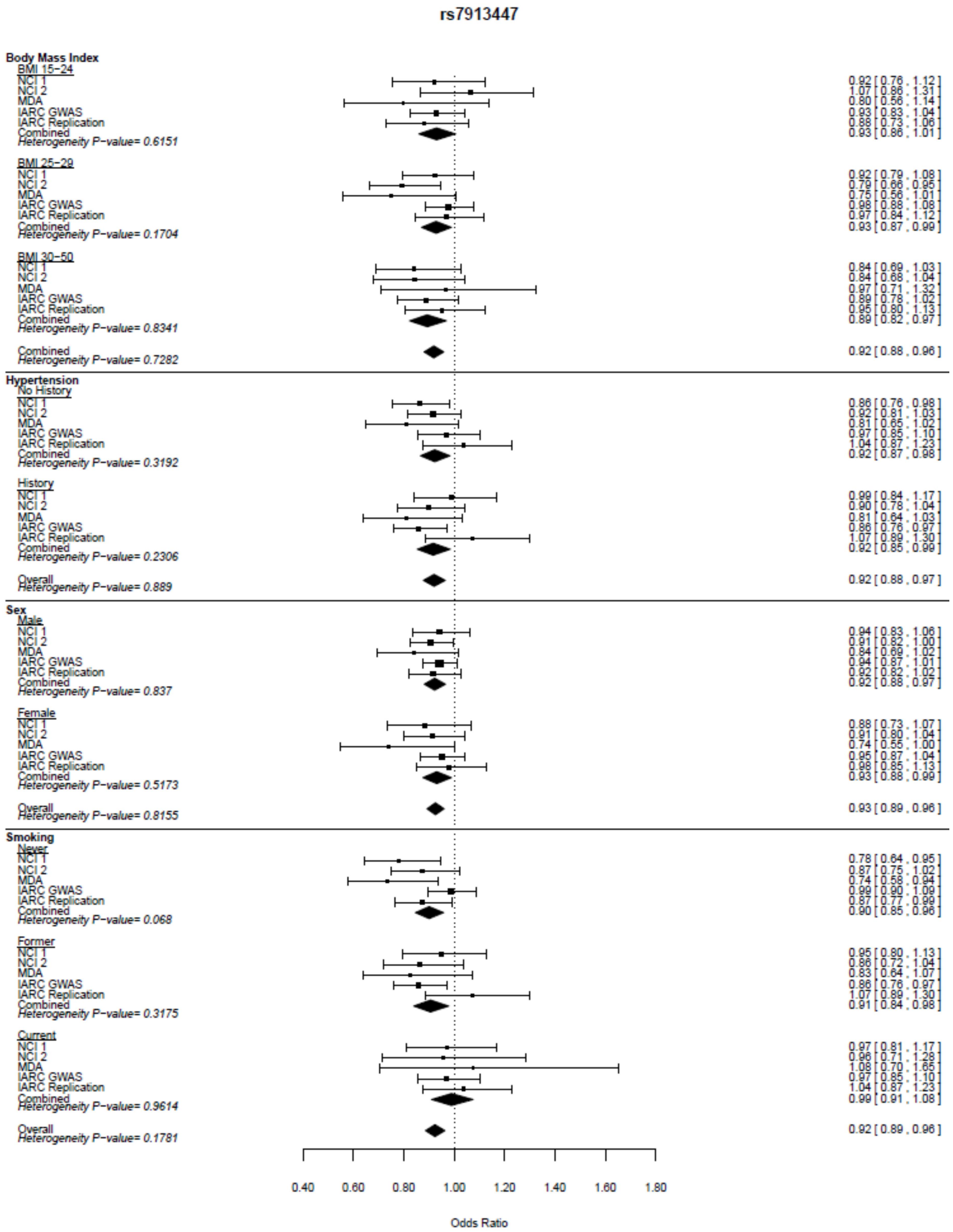

rs7913447
Body Mass Index
BMI 15−24
NCI 1 0.92 [0.76 , 1.12]
NCI 2 1.07 [0.86 , 1.31]
MDA 0.80 [0.56 , 1.14]
IARC GWAS 0.93 [0.83 , 1.04]
IARC Replication 0.88 [0.73 , 1.06]
Combined 0.93 [0.86 , 1.01]
Heterogeneity P−value= 0.6151
BMI 25−29
NCI 1 0.92 [0.79 , 1.08]
NCI 2 0.79 [0.66 , 0.95]
MDA 0.75 [0.56 , 1.01]
IARC GWAS 0.98 [0.88 , 1.08]
IARC Replication 0.97 [0.84 , 1.12]
Combined 0.93 [0.87 , 0.99]
Heterogeneity P−value= 0.1704
BMI 30−50
NCI 1 0.84 [0.69 , 1.03]
NCI 2 0.84 [0.68 , 1.04]
MDA 0.97 [0.71 , 1.32]
IARC GWAS 0.89 [0.78 , 1.02]
IARC Replication 0.95 [0.80 , 1.13]
Combined 0.89 [0.82 , 0.97]
Heterogeneity P−value= 0.8341
Combined 0.92 [0.88 , 0.96]
Heterogeneity P−value= 0.7282
Hypertension
No History
NCI 1 0.86 [0.76 , 0.98]
NCI 2 0.92 [0.81 , 1.03]
MDA 0.81 [0.65 , 1.02]
IARC GWAS 0.97 [0.85 , 1.10]
IARC Replication 1.04 [0.87 , 1.23]
Combined 0.92 [0.87 , 0.98]
Heterogeneity P−value= 0.3192
History
NCI 1 0.99 [0.84 , 1.17]
NCI 2 0.90 [0.78 , 1.04]
MDA 0.81 [0.64 , 1.03]
IARC GWAS 0.86 [0.76 , 0.97]
IARC Replication 1.07 [0.89 , 1.30]
Combined 0.92 [0.85 , 0.99]
Heterogeneity P−value= 0.2306
Overall 0.92 [0.88 , 0.97]
Heterogeneity P−value= 0.889
Sex
Male
NCI 1 0.94 [0.83 , 1.06]
NCI 2 0.91 [0.82 , 1.00]
MDA 0.84 [0.69 , 1.02]
IARC GWAS 0.94 [0.87 , 1.01]
IARC Replication 0.92 [0.82 , 1.02]
Combined 0.92 [0.88 , 0.97]
Heterogeneity P−value= 0.837
Female
NCI 1 0.88 [0.73 , 1.07]
NCI 2 0.91 [0.80 , 1.04]
MDA 0.74 [0.55 , 1.00]
IARC GWAS 0.95 [0.87 , 1.04]
IARC Replication 0.98 [0.85 , 1.13]
Combined 0.93 [0.88 , 0.99]
Heterogeneity P−value= 0.5173
Overall 0.93 [0.89 , 0.96]
Heterogeneity P−value= 0.8155
Smoking
Never
NCI 1 0.78 [0.64 , 0.95]
NCI 2 0.87 [0.75 , 1.02]
MDA 0.74 [0.58 , 0.94]
IARC GWAS 0.99 [0.90 , 1.09]
IARC Replication 0.87 [0.77 , 0.99]
Combined 0.90 [0.85 , 0.96]
Heterogeneity P−value= 0.068
Former
NCI 1 0.95 [0.80 , 1.13]
NCI 2 0.86 [0.72 , 1.04]
MDA 0.83 [0.64 , 1.07]
IARC GWAS 0.86 [0.76 , 0.97]
IARC Replication 1.07 [0.89 , 1.30]
Combined 0.91 [0.84 , 0.98]
Heterogeneity P−value= 0.3175
Current
NCI 1 0.97 [0.81 , 1.17]
NCI 2 0.96 [0.71 , 1.28]
MDA 1.08 [0.70 , 1.65]
IARC GWAS 0.97 [0.85 , 1.10]
IARC Replication 1.04 [0.87 , 1.23]
Combined 0.99 [0.91 , 1.08]
Heterogeneity P−value= 0.9614
Overall 0.92 [0.89 , 0.96]
Heterogeneity P−value= 0.1781
0.40 0.60 0.80 1.00 1.20 1.40 1.60 1.80
Odds Ratio

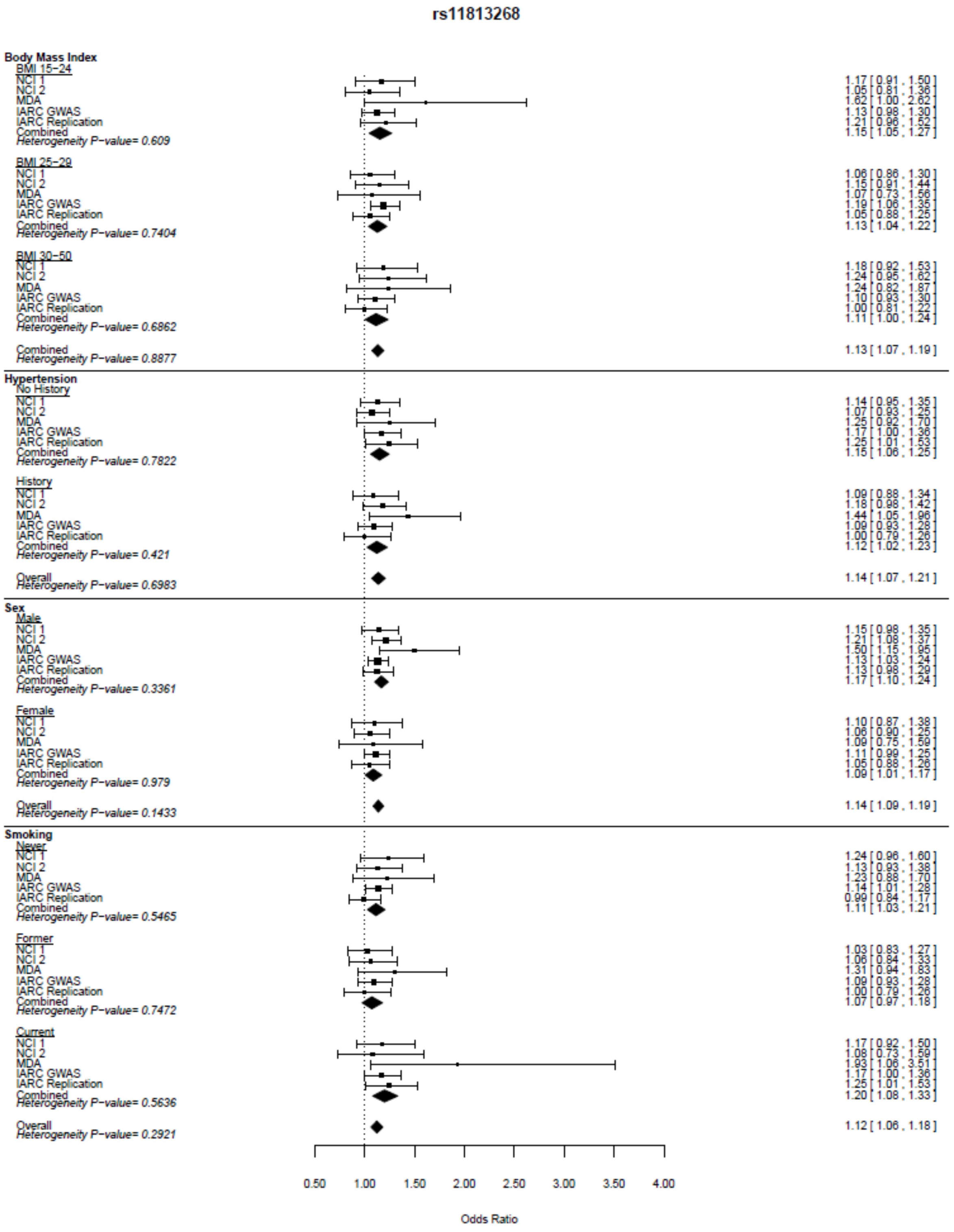
rs11813268
Body Mass Index
BMI 15−24
NCI 1 1.17 [0.91 , 1.50]
NCI 2 1.05 [0.81 , 1.36]
MDA 1.62 [1.00 , 2.62]
IARC GWAS 1.13 [0.98 , 1.30]
IARC Replication 1.21 [0.96 , 1.52]
Combined 1.15 [1.05 , 1.27]
Heterogeneity P−value= 0.609
BMI 25−29
NCI 1 1.06 [0.86 , 1.30]
NCI 2 1.15 [0.91 , 1.44]
MDA 1.07 [0.73 , 1.56]
IARC GWAS 1.19 [1.06 , 1.35]
IARC Replication 1.05 [0.88 , 1.25]
Combined 1.13 [1.04 , 1.22]
Heterogeneity P−value= 0.7404
BMI 30−50
NCI 1 1.18 [0.92 , 1.53]
NCI 2 1.24 [0.95 , 1.62]
MDA 1.24 [0.82 , 1.87]
IARC GWAS 1.10 [0.93 , 1.30]
IARC Replication 1.00 [0.81 , 1.22]
Combined 1.11 [1.00 , 1.24]
Heterogeneity P−value= 0.6862
Combined 1.13 [1.07 , 1.19]
Heterogeneity P−value= 0.8877
Hypertension
No History
NCI 1 1.14 [0.95 , 1.35]
NCI 2 1.07 [0.93 , 1.25]
MDA 1.25 [0.92 , 1.70]
IARC GWAS 1.17 [1.00 , 1.36]
IARC Replication 1.25 [1.01 , 1.53]
Combined 1.15 [1.06 , 1.25]
Heterogeneity P−value= 0.7822
History
NCI 1 1.09 [0.88 , 1.34]
NCI 2 1.18 [0.98 , 1.42]
MDA 1.44 [1.05 , 1.96]
IARC GWAS 1.09 [0.93 , 1.28]
IARC Replication 1.00 [0.79 , 1.26]
Combined 1.12 [1.02 , 1.23]
Heterogeneity P−value= 0.421
Overall 1.14 [1.07 , 1.21]
Heterogeneity P−value= 0.6983
Sex
Male
NCI 1 1.15 [0.98 , 1.35]
NCI 2 1.21 [1.08 , 1.37]
MDA 1.50 [1.15 , 1.95]
IARC GWAS 1.13 [1.03 , 1.24]
IARC Replication 1.13 [0.98 , 1.29]
Combined 1.17 [1.10 , 1.24]
Heterogeneity P−value= 0.3361
Female
NCI 1 1.10 [0.87 , 1.38]
NCI 2 1.06 [0.90 , 1.25]
MDA 1.09 [0.75 , 1.59]
IARC GWAS 1.11 [0.99 , 1.25]
IARC Replication 1.05 [0.88 , 1.26]
Combined 1.09 [1.01 , 1.17]
Heterogeneity P−value= 0.979
Overall 1.14 [1.09 , 1.19]
Heterogeneity P−value= 0.1433
Smoking
Never
NCI 1 1.24 [0.96 , 1.60]
NCI 2 1.13 [0.93 , 1.38]
MDA 1.23 [0.88 , 1.70]
IARC GWAS 1.14 [1.01 , 1.28]
IARC Replication 0.99 [0.84 , 1.17]
Combined 1.11 [1.03 , 1.21]
Heterogeneity P−value= 0.5465
Former
NCI 1 1.03 [0.83 , 1.27]
NCI 2 1.06 [0.84 , 1.33]
MDA 1.31 [0.94 , 1.83]
IARC GWAS 1.09 [0.93 , 1.28]
IARC Replication 1.00 [0.79 , 1.26]
Combined 1.07 [0.97 , 1.18]
Heterogeneity P−value= 0.7472
Current
NCI 1 1.17 [0.92 , 1.50]
NCI 2 1.08 [0.73 , 1.59]
MDA 1.93 [1.06 , 3.51]
IARC GWAS 1.17 [1.00 , 1.36]
IARC Replication 1.25 [1.01 , 1.53]
Combined 1.20 [1.08 , 1.33]
Heterogeneity P−value= 0.5636
Overall 1.12 [1.06 , 1.18]
Heterogeneity P−value= 0.2921
0.50 1.00 1.50 2.00 2.50 3.00 3.50 4.00
Odds Ratio

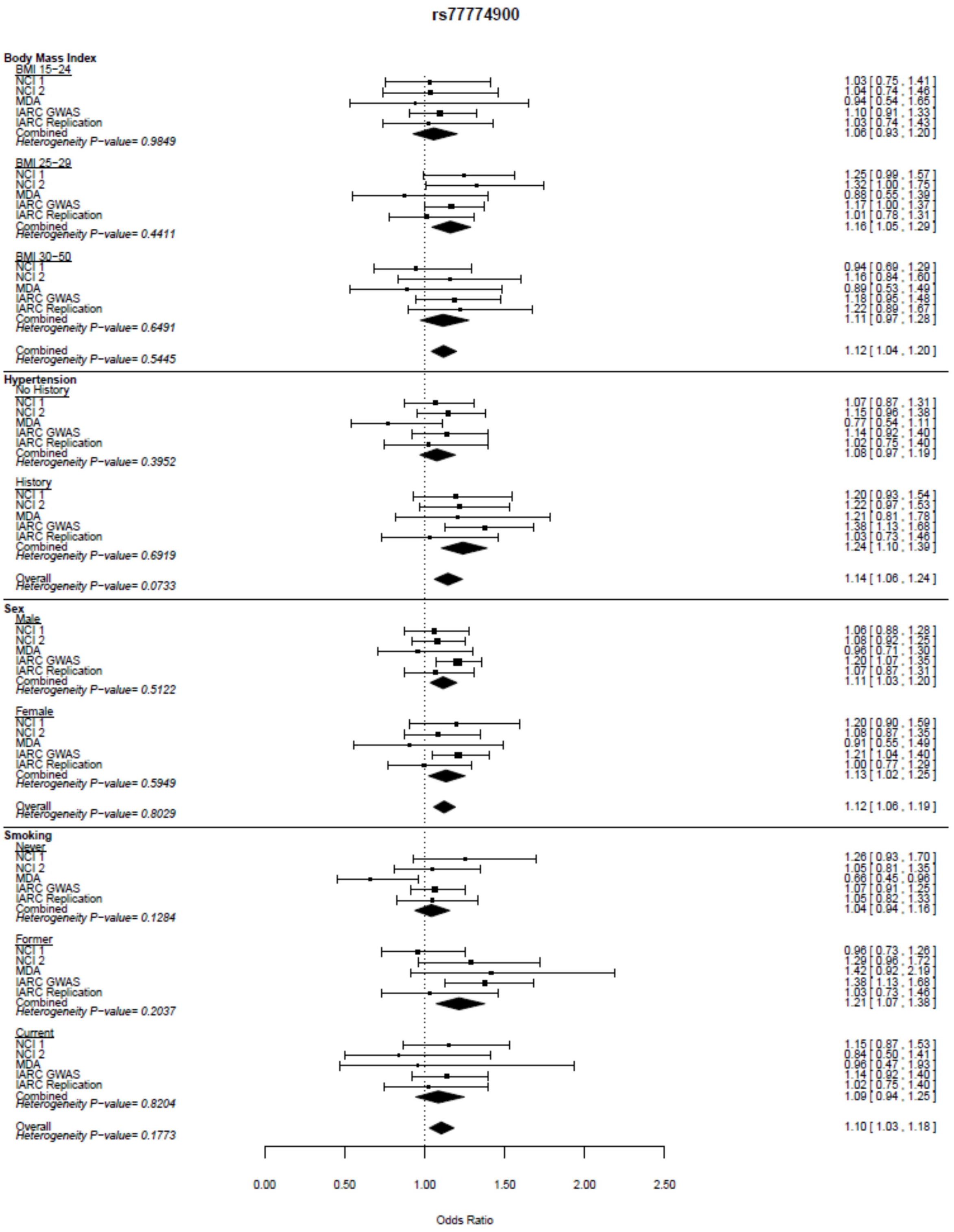
rs77774900
Body Mass Index
BMI 15−24
NCI 1 1.03 [0.75 , 1.41]
NCI 2 1.04 [0.74 , 1.46]
MDA 0.94 [0.54 , 1.65]
IARC GWAS 1.10 [0.91 , 1.33]
IARC Replication 1.03 [0.74 , 1.43]
Combined 1.06 [0.93 , 1.20]
Heterogeneity P−value= 0.9849
BMI 25−29
NCI 1 1.25 [0.99 , 1.57]
NCI 2 1.32 [1.00 , 1.75]
MDA 0.88 [0.55 , 1.39]
IARC GWAS 1.17 [1.00 , 1.37]
IARC Replication 1.01 [0.78 , 1.31]
Combined 1.16 [1.05 , 1.29]
Heterogeneity P−value= 0.4411
BMI 30−50
NCI 1 0.94 [0.69 , 1.29]
NCI 2 1.16 [0.84 , 1.60]
MDA 0.89 [0.53 , 1.49]
IARC GWAS 1.18 [0.95 , 1.48]
IARC Replication 1.22 [0.89 , 1.67]
Combined 1.11 [0.97 , 1.28]
Heterogeneity P−value= 0.6491
Combined 1.12 [1.04 , 1.20]
Heterogeneity P−value= 0.5445
Hypertension
No History
NCI 1 1.07 [0.87 , 1.31]
NCI 2 1.15 [0.96 , 1.38]
MDA 0.77 [0.54 , 1.11]
IARC GWAS 1.14 [0.92 , 1.40]
IARC Replication 1.02 [0.75 , 1.40]
Combined 1.08 [0.97 , 1.19]
Heterogeneity P−value= 0.3952
History
NCI 1 1.20 [0.93 , 1.54]
NCI 2 1.22 [0.97 , 1.53]
MDA 1.21 [0.81 , 1.78]
IARC GWAS 1.38 [1.13 , 1.68]
IARC Replication 1.03 [0.73 , 1.46]
Combined 1.24 [1.10 , 1.39]
Heterogeneity P−value= 0.6919
Overall 1.14 [1.06 , 1.24]
Heterogeneity P−value= 0.0733
Sex
Male
NCI 1 1.06 [0.88 , 1.28]
NCI 2 1.08 [0.92 , 1.25]
MDA 0.96 [0.71 , 1.30]
IARC GWAS 1.20 [1.07 , 1.35]
IARC Replication 1.07 [0.87 , 1.31]
Combined 1.11 [1.03 , 1.20]
Heterogeneity P−value= 0.5122
Female
NCI 1 1.20 [0.90 , 1.59]
NCI 2 1.08 [0.87 , 1.35]
MDA 0.91 [0.55 , 1.49]
IARC GWAS 1.21 [1.04 , 1.40]
IARC Replication 1.00 [0.77 , 1.29]
Combined 1.13 [1.02 , 1.25]
Heterogeneity P−value= 0.5949
Overall 1.12 [1.06 , 1.19]
Heterogeneity P−value= 0.8029
Smoking
Never
NCI 1 1.26 [0.93 , 1.70]
NCI 2 1.05 [0.81 , 1.35]
MDA 0.66 [0.45 , 0.96]
IARC GWAS 1.07 [0.91 , 1.25]
IARC Replication 1.05 [0.82 , 1.33]
Combined 1.04 [0.94 , 1.16]
Heterogeneity P−value= 0.1284
Former
NCI 1 0.96 [0.73 , 1.26]
NCI 2 1.29 [0.96 , 1.72]
MDA 1.42 [0.92 , 2.19]
IARC GWAS 1.38 [1.13 , 1.68]
IARC Replication 1.03 [0.73 , 1.46]
Combined 1.21 [1.07 , 1.38]
Heterogeneity P−value= 0.2037
Current
NCI 1 1.15 [0.87 , 1.53]
NCI 2 0.84 [0.50 , 1.41]
MDA 0.96 [0.47 , 1.93]
IARC GWAS 1.14 [0.92 , 1.40]
IARC Replication 1.02 [0.75 , 1.40]
Combined 1.09 [0.94 , 1.25]
Heterogeneity P−value= 0.8204
Overall 1.10 [1.03 , 1.18]
Heterogeneity P−value= 0.1773
0.00
0.50
1.00
1.50
2.00
2.50
Odds Ratio

rs1800057

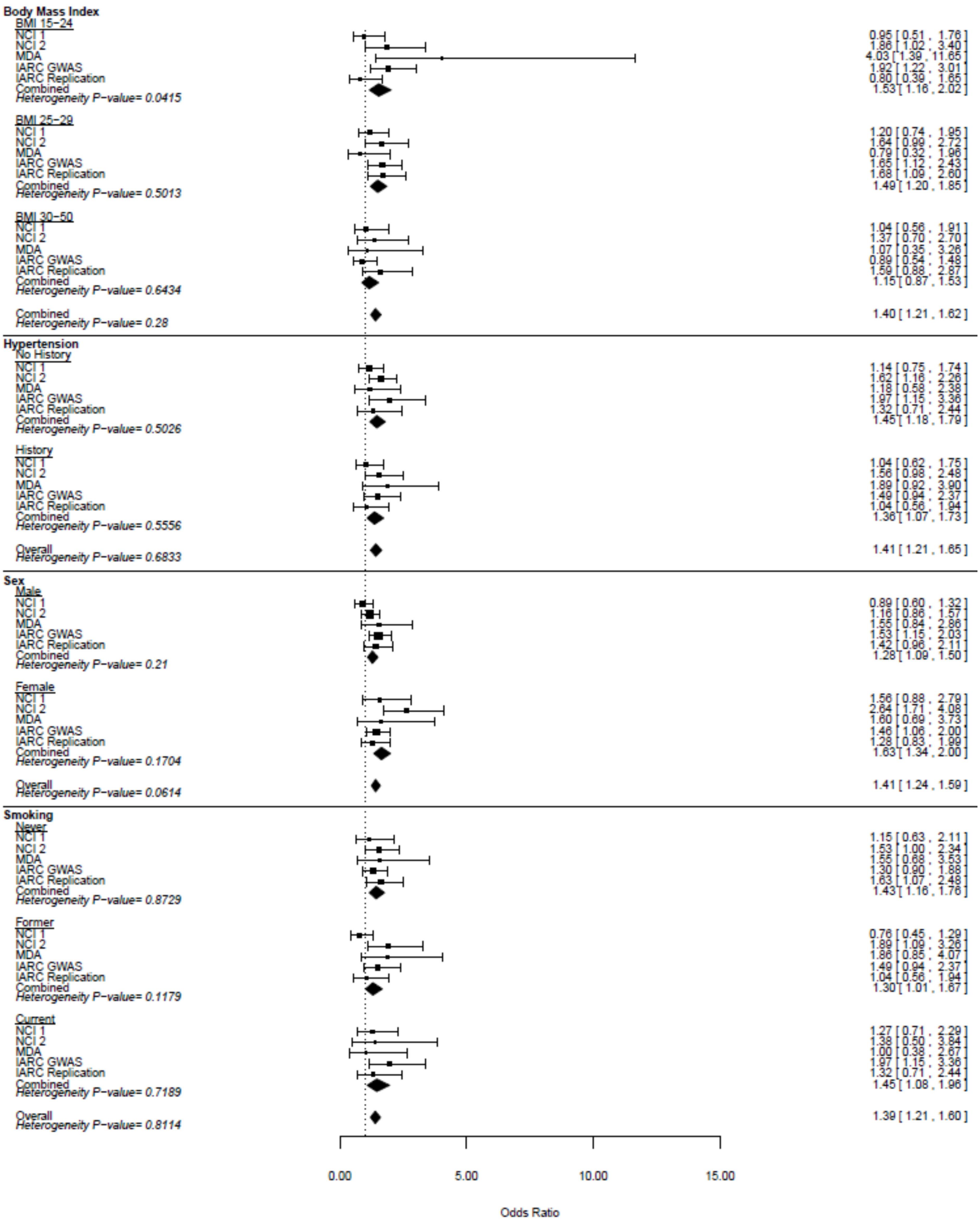
Body Mass Index
BMI 15−24
NCI 1 0.95 [0.51 , 1.76]
NCI 2 1.86 [1.02 , 3.40]
MDA 4.03 [1.39 , 11.65]
IARC GWAS 1.92 [1.22 , 3.01]
IARC Replication 0.80 [0.39 , 1.65]
Combined 1.53 [1.16 , 2.02]
Heterogeneity P−value= 0.0415
BMI 25−29
NCI 1 1.20 [0.74 , 1.95]
NCI 2 1.64 [0.99 , 2.72]
MDA 0.79 [0.32 , 1.96]
IARC GWAS 1.65 [1.12 , 2.43]
IARC Replication 1.68 [1.09 , 2.60]
Combined 1.49 [1.20 , 1.85]
Heterogeneity P−value= 0.5013
BMI 30−50
NCI 1 1.04 [0.56 , 1.91]
NCI 2 1.37 [0.70 , 2.70]
MDA 1.07 [0.35 , 3.26]
IARC GWAS 0.89 [0.54 , 1.48]
IARC Replication 1.59 [0.88 , 2.87]
Combined 1.15 [0.87 , 1.53]
Heterogeneity P−value= 0.6434
Combined 1.40 [1.21 , 1.62]
Heterogeneity P−value= 0.28
Hypertension
No History
NCI 1 1.14 [0.75 , 1.74]
NCI 2 1.62 [1.16 , 2.26]
MDA 1.18 [0.58 , 2.38]
IARC GWAS 1.97 [1.15 , 3.36]
IARC Replication 1.32 [0.71 , 2.44]
Combined 1.45 [1.18 , 1.79]
Heterogeneity P−value= 0.5026
History
NCI 1 1.04 [0.62 , 1.75]
NCI 2 1.56 [0.98 , 2.48]
MDA 1.89 [0.92 , 3.90]
IARC GWAS 1.49 [0.94 , 2.37]
IARC Replication 1.04 [0.56 , 1.94]
Combined 1.36 [1.07 , 1.73]
Heterogeneity P−value= 0.5556
Overall 1.41 [1.21 , 1.65]
Heterogeneity P−value= 0.6833
Sex
Male
NCI 1 0.89 [0.60 , 1.32]
NCI 2 1.16 [0.86 , 1.57]
MDA 1.55 [0.84 , 2.86]
IARC GWAS 1.53 [1.15 , 2.03]
IARC Replication 1.42 [0.96 , 2.11]
Combined 1.28 [1.09 , 1.50]
Heterogeneity P−value= 0.21
Female
NCI 1 1.56 [0.88 , 2.79]
NCI 2 2.64 [1.71 , 4.08]
MDA 1.60 [0.69 , 3.73]
IARC GWAS 1.46 [1.06 , 2.00]
IARC Replication 1.28 [0.83 , 1.99]
Combined 1.63 [1.34 , 2.00]
Heterogeneity P−value= 0.1704
Overall 1.41 [1.24 , 1.59]
Heterogeneity P−value= 0.0614
Smoking
Never
NCI 1 1.15 [0.63 , 2.11]
NCI 2 1.53 [1.00 , 2.34]
MDA 1.55 [0.68 , 3.53]
IARC GWAS 1.30 [0.90 , 1.88]
IARC Replication 1.63 [1.07 , 2.48]
Combined 1.43 [1.16 , 1.76]
Heterogeneity P−value= 0.8729
Former
NCI 1 0.76 [0.45 , 1.29]
NCI 2 1.89 [1.09 , 3.26]
MDA 1.86 [0.85 , 4.07]
IARC GWAS 1.49 [0.94 , 2.37]
IARC Replication 1.04 [0.56 , 1.94]
Combined 1.30 [1.01 , 1.67]
Heterogeneity P−value= 0.1179
Current
NCI 1 1.27 [0.71 , 2.29]
NCI 2 1.38 [0.50 , 3.84]
MDA 1.00 [0.38 , 2.67]
IARC GWAS 1.97 [1.15 , 3.36]
IARC Replication 1.32 [0.71 , 2.44]
Combined 1.45 [1.08 , 1.96]
Heterogeneity P−value= 0.7189
Overall 1.39 [1.21 , 1.60]
Heterogeneity P−value= 0.8114
0.00
5.00
10.00
15.00
Odds Ratio

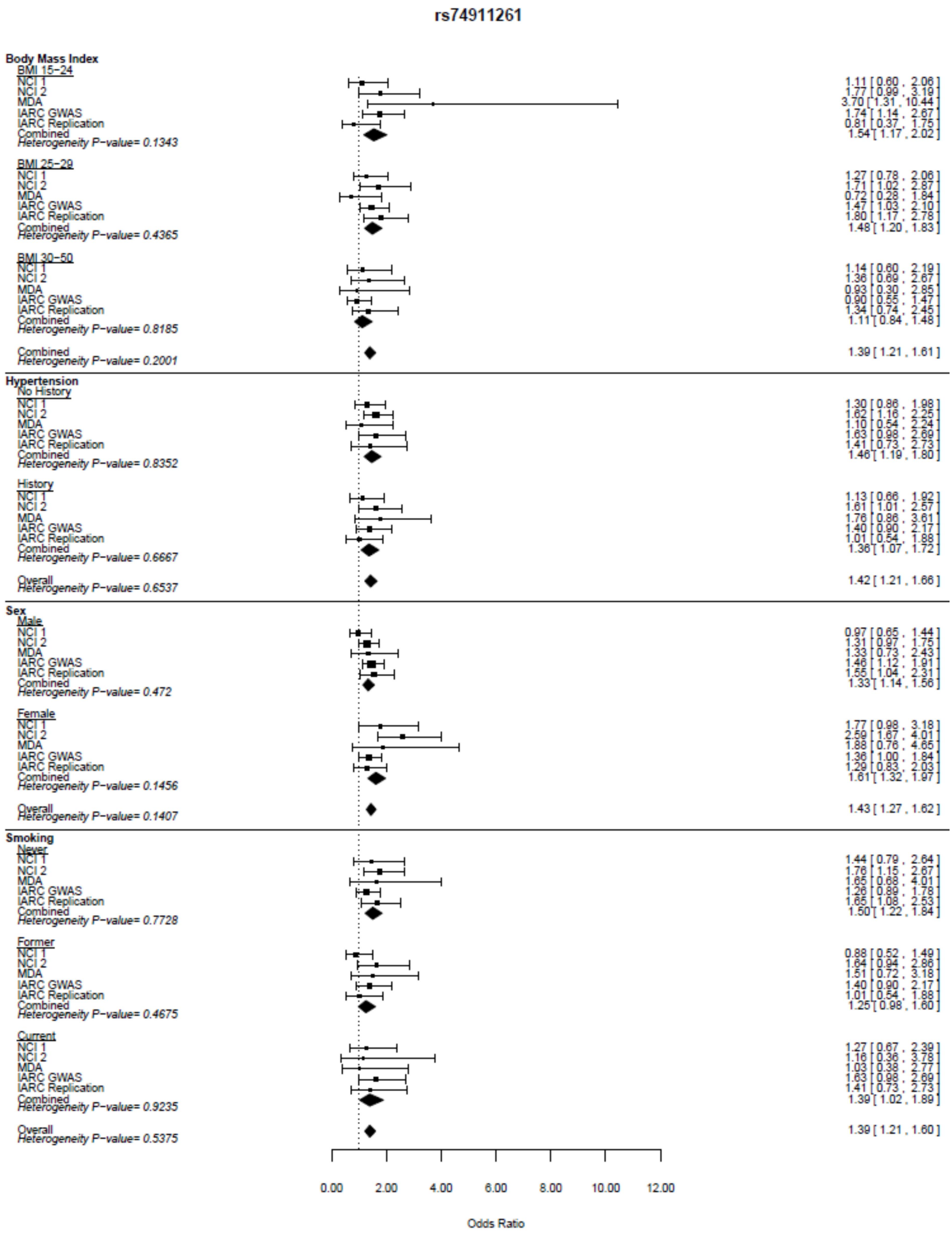
rs74911261
Body Mass Index
BMI 15−24
NCI 1
NCI 2
MDA
IARC GWAS
IARC Replication
Combined
Heterogeneity P−value= 0.1343
1.11 [0.60 , 2.06]
1.77 [0.99 , 3.19]
3.70 [1.31 , 10.44]
1.74 [1.14 , 2.67]
0.81 [0.37 , 1.75]
1.54 [1.17 , 2.02]
BMI 25−29
NCI 1
NCI 2
MDA
IARC GWAS
IARC Replication
Combined
Heterogeneity P−value= 0.4365
1.27 [0.78 , 2.06]
1.71 [1.02 , 2.87]
0.72 [0.28 , 1.84]
1.47 [1.03 , 2.10]
1.80 [1.17 , 2.78]
1.48 [1.20 , 1.83]
BMI 30−50
NCI 1
NCI 2
MDA
IARC GWAS
IARC Replication
Combined
Heterogeneity P−value= 0.8185
1.14 [0.60 , 2.19]
1.36 [0.69 , 2.67]
0.93 [0.30 , 2.85]
0.90 [0.55 , 1.47]
1.34 [0.74 , 2.45]
1.11 [0.84 , 1.48]
Combined
Heterogeneity P−value= 0.2001
1.39 [1.21 , 1.61]
Hypertension
No History
NCI 1
NCI 2
MDA
IARC GWAS
IARC Replication
Combined
Heterogeneity P−value= 0.8352
1.30 [0.86 , 1.98]
1.62 [1.16 , 2.25]
1.10 [0.54 , 2.24]
1.63 [0.98 , 2.69]
1.41 [0.73 , 2.73]
1.46 [1.19 , 1.80]
History
NCI 1
NCI 2
MDA
IARC GWAS
IARC Replication
Combined
Heterogeneity P−value= 0.6667
1.13 [0.66 , 1.92]
1.61 [1.01 , 2.57]
1.76 [0.86 , 3.61]
1.40 [0.90 , 2.17]
1.01 [0.54 , 1.88]
1.36 [1.07 , 1.72]
Overall
Heterogeneity P−value= 0.6537
1.42 [1.21 , 1.66]
Sex
Male
NCI 1
NCI 2
MDA
IARC GWAS
IARC Replication
Combined
Heterogeneity P−value= 0.472
0.97 [0.65 , 1.44]
1.31 [0.97 , 1.75]
1.33 [0.73 , 2.43]
1.46 [1.12 , 1.91]
1.55 [1.04 , 2.31]
1.33 [1.14 , 1.56]
Female
NCI 1
NCI 2
MDA
IARC GWAS
IARC Replication
Combined
Heterogeneity P−value= 0.1456
1.77 [0.98 , 3.18]
2.59 [1.67 , 4.01]
1.88 [0.76 , 4.65]
1.36 [1.00 , 1.84]
1.29 [0.83 , 2.03]
1.61 [1.32 , 1.97]
Overall
Heterogeneity P−value= 0.1407
1.43 [1.27 , 1.62]
Smoking
Never
NCI 1
NCI 2
MDA
IARC GWAS
IARC Replication
Combined
Heterogeneity P−value= 0.7728
1.44 [0.79 , 2.64]
1.76 [1.15 , 2.67]
1.65 [0.68 , 4.01]
1.26 [0.89 , 1.78]
1.65 [1.08 , 2.53]
1.50 [1.22 , 1.84]
Former
NCI 1
NCI 2
MDA
IARC GWAS
IARC Replication
Combined
Heterogeneity P−value= 0.4675
0.88 [0.52 , 1.49]
1.64 [0.94 , 2.86]
1.51 [0.72 , 3.18]
1.40 [0.90 , 2.17]
1.01 [0.54 , 1.88]
1.25 [0.98 , 1.60]
Current
NCI 1
NCI 2
MDA
IARC GWAS
IARC Replication
Combined
Heterogeneity P−value= 0.9235
1.27 [0.67 , 2.39]
1.16 [0.36 , 3.78]
1.03 [0.38 , 2.77]
1.63 [0.98 , 2.69]
1.41 [0.73 , 2.73]
1.39 [1.02 , 1.89]
Overall
Heterogeneity P−value= 0.5375
1.39 [1.21 , 1.60]
0.00
2.00
4.00
6.00
8.00
10.00
12.00
Odds Ratio

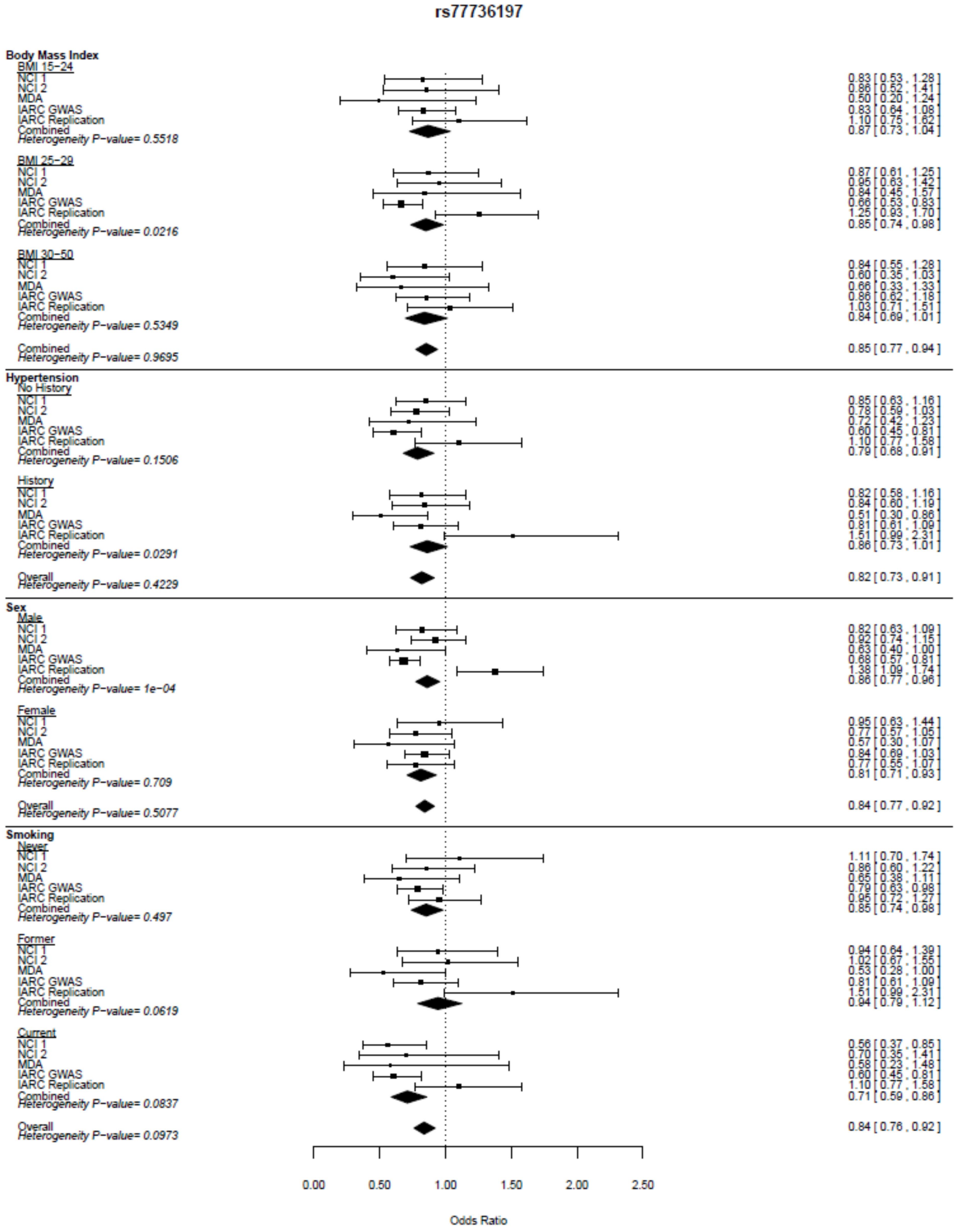
rs77736197
Body Mass Index
BMI 15−24
NCI 1 0.83 [0.53 , 1.28]
NCI 2 0.86 [0.52 , 1.41]
MDA 0.50 [0.20 , 1.24]
IARC GWAS 0.83 [0.64 , 1.08]
IARC Replication 1.10 [0.75 , 1.62]
Combined 0.87 [0.73 , 1.04]
Heterogeneity P−value= 0.5518
BMI 25−29
NCI 1 0.87 [0.61 , 1.25]
NCI 2 0.95 [0.63 , 1.42]
MDA 0.84 [0.45 , 1.57]
IARC GWAS 0.66 [0.53 , 0.83]
IARC Replication 1.25 [0.93 , 1.70]
Combined 0.85 [0.74 , 0.98]
Heterogeneity P−value= 0.0216
BMI 30−50
NCI 1 0.84 [0.55 , 1.28]
NCI 2 0.60 [0.35 , 1.03]
MDA 0.66 [0.33 , 1.33]
IARC GWAS 0.86 [0.62 , 1.18]
IARC Replication 1.03 [0.71 , 1.51]
Combined 0.84 [0.69 , 1.01]
Heterogeneity P−value= 0.5349
Combined 0.85 [0.77 , 0.94]
Heterogeneity P−value= 0.9695
Hypertension
No History
NCI 1 0.85 [0.63 , 1.16]
NCI 2 0.78 [0.59 , 1.03]
MDA 0.72 [0.42 , 1.23]
IARC GWAS 0.60 [0.45 , 0.81]
IARC Replication 1.10 [0.77 , 1.58]
Combined 0.79 [0.68 , 0.91]
Heterogeneity P−value= 0.1506
History
NCI 1 0.82 [0.58 , 1.16]
NCI 2 0.84 [0.60 , 1.19]
MDA 0.51 [0.30 , 0.86]
IARC GWAS 0.81 [0.61 , 1.09]
IARC Replication 1.51 [0.99 , 2.31]
Combined 0.86 [0.73 , 1.01]
Heterogeneity P−value= 0.0291
Overall 0.82 [0.73 , 0.91]
Heterogeneity P−value= 0.4229
Sex
Male
NCI 1 0.82 [0.63 , 1.09]
NCI 2 0.92 [0.74 , 1.15]
MDA 0.63 [0.40 , 1.00]
IARC GWAS 0.68 [0.57 , 0.81]
IARC Replication 1.38 [1.09 , 1.74]
Combined 0.86 [0.77 , 0.96]
Heterogeneity P−value= 1e−04
Female
NCI 1 0.95 [0.63 , 1.44]
NCI 2 0.77 [0.57 , 1.05]
MDA 0.57 [0.30 , 1.07]
IARC GWAS 0.84 [0.69 , 1.03]
IARC Replication 0.77 [0.55 , 1.07]
Combined 0.81 [0.71 , 0.93]
Heterogeneity P−value= 0.709
Overall 0.84 [0.77 , 0.92]
Heterogeneity P−value= 0.5077
Smoking
Never
NCI 1 1.11 [0.70 , 1.74]
NCI 2 0.86 [0.60 , 1.22]
MDA 0.65 [0.38 , 1.11]
IARC GWAS 0.79 [0.63 , 0.98]
IARC Replication 0.95 [0.72 , 1.27]
Combined 0.85 [0.74 , 0.98]
Heterogeneity P−value= 0.497
Former
NCI 1 0.94 [0.64 , 1.39]
NCI 2 1.02 [0.67 , 1.55]
MDA 0.53 [0.28 , 1.00]
IARC GWAS 0.81 [0.61 , 1.09]
IARC Replication 1.51 [0.99 , 2.31]
Combined 0.94 [0.79 , 1.12]
Heterogeneity P−value= 0.0619
Current
NCI 1 0.56 [0.37 , 0.85]
NCI 2 0.70 [0.35 , 1.41]
MDA 0.58 [0.23 , 1.48]
IARC GWAS 0.60 [0.45 , 0.81]
IARC Replication 1.10 [0.77 , 1.58]
Combined 0.71 [0.59 , 0.86]
Heterogeneity P−value= 0.0837
Overall 0.84 [0.76 , 0.92]
Heterogeneity P−value= 0.0973
0.00
0.50
1.00
1.50
2.00
2.50
Odds Ratio

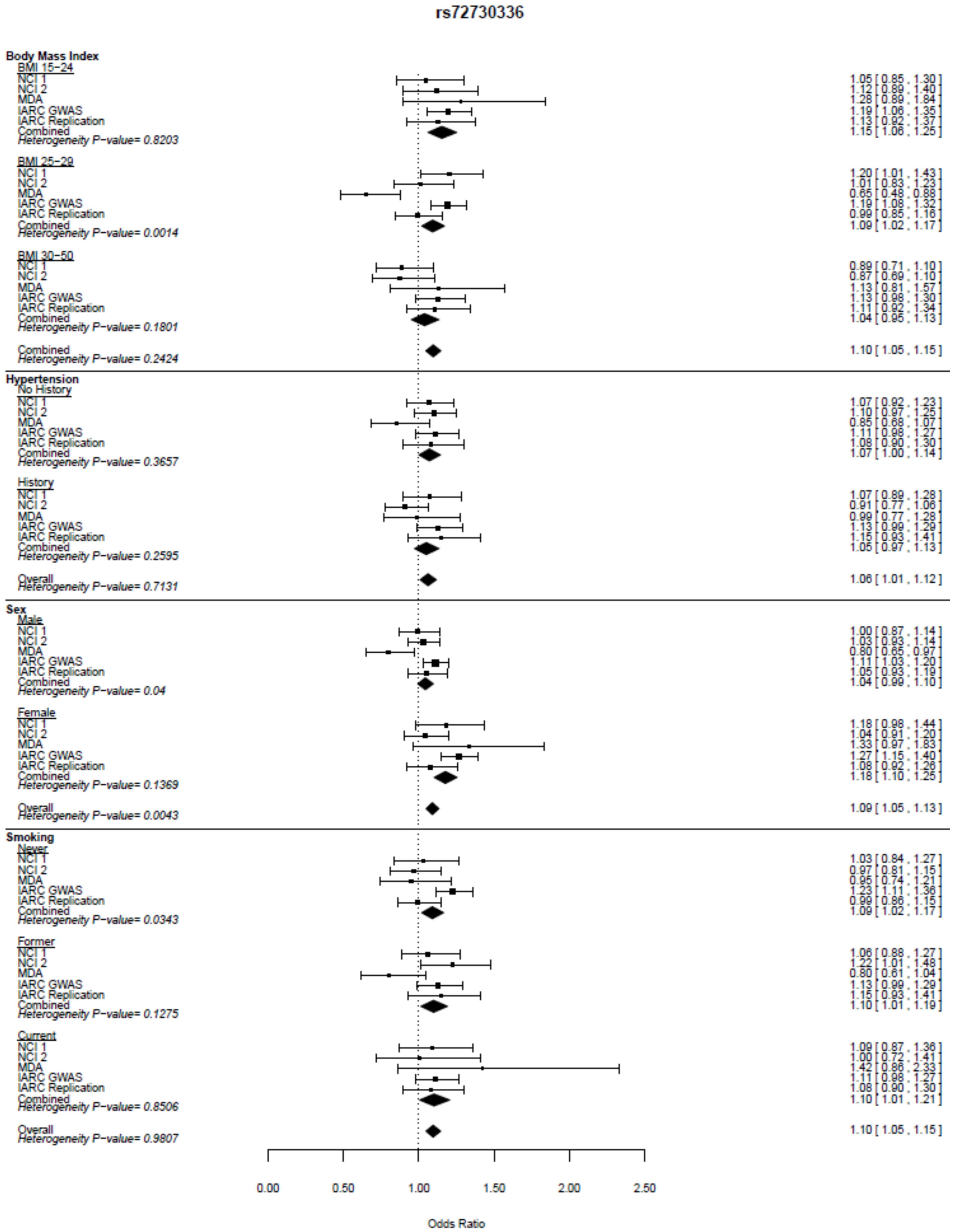
rs72730336
Body Mass Index
BMI 15−24
NCI 1 1.05 [0.85 , 1.30]
NCI 2 1.12 [0.89 , 1.40]
MDA 1.28 [0.89 , 1.84]
IARC GWAS 1.19 [1.06 , 1.35]
IARC Replication 1.13 [0.92 , 1.37]
Combined 1.15 [1.06 , 1.25]
Heterogeneity P−value= 0.8203
BMI 25−29
NCI 1 1.20 [1.01 , 1.43]
NCI 2 1.01 [0.83 , 1.23]
MDA 0.65 [0.48 , 0.88]
IARC GWAS 1.19 [1.08 , 1.32]
IARC Replication 0.99 [0.85 , 1.16]
Combined 1.09 [1.02 , 1.17]
Heterogeneity P−value= 0.0014
BMI 30−50
NCI 1 0.89 [0.71 , 1.10]
NCI 2 0.87 [0.69 , 1.10]
MDA 1.13 [0.81 , 1.57]
IARC GWAS 1.13 [0.98 , 1.30]
IARC Replication 1.11 [0.92 , 1.34]
Combined 1.04 [0.95 , 1.13]
Heterogeneity P−value= 0.1801
Combined 1.10 [1.05 , 1.15]
Heterogeneity P−value= 0.2424
Hypertension
No History
NCI 1 1.07 [0.92 , 1.23]
NCI 2 1.10 [0.97 , 1.25]
MDA 0.85 [0.68 , 1.07]
IARC GWAS 1.11 [0.98 , 1.27]
IARC Replication 1.08 [0.90 , 1.30]
Combined 1.07 [1.00 , 1.14]
Heterogeneity P−value= 0.3657
History
NCI 1 1.07 [0.89 , 1.28]
NCI 2 0.91 [0.77 , 1.06]
MDA 0.99 [0.77 , 1.28]
IARC GWAS 1.13 [0.99 , 1.29]
IARC Replication 1.15 [0.93 , 1.41]
Combined 1.05 [0.97 , 1.13]
Heterogeneity P−value= 0.2595
Overall 1.06 [1.01 , 1.12]
Heterogeneity P−value= 0.7131
Sex
Male
NCI 1 1.00 [0.87 , 1.14]
NCI 2 1.03 [0.93 , 1.14]
MDA 0.80 [0.65 , 0.97]
IARC GWAS 1.11 [1.03 , 1.20]
IARC Replication 1.05 [0.93 , 1.19]
Combined 1.04 [0.99 , 1.10]
Heterogeneity P−value= 0.04
Female
NCI 1 1.18 [0.98 , 1.44]
NCI 2 1.04 [0.91 , 1.20]
MDA 1.33 [0.97 , 1.83]
IARC GWAS 1.27 [1.15 , 1.40]
IARC Replication 1.08 [0.92 , 1.26]
Combined 1.18 [1.10 , 1.25]
Heterogeneity P−value= 0.1369
Overall 1.09 [1.05 , 1.13]
Heterogeneity P−value= 0.0043
Smoking
Never
NCI 1 1.03 [0.84 , 1.27]
NCI 2 0.97 [0.81 , 1.15]
MDA 0.95 [0.74 , 1.21]
IARC GWAS 1.23 [1.11 , 1.36]
IARC Replication 0.99 [0.86 , 1.15]
Combined 1.09 [1.02 , 1.17]
Heterogeneity P−value= 0.0343
Former
NCI 1 1.06 [0.88 , 1.27]
NCI 2 1.22 [1.01 , 1.48]
MDA 0.80 [0.61 , 1.04]
IARC GWAS 1.13 [0.99 , 1.29]
IARC Replication 1.15 [0.93 , 1.41]
Combined 1.10 [1.01 , 1.19]
Heterogeneity P−value= 0.1275
Current
NCI 1 1.09 [0.87 , 1.36]
NCI 2 1.00 [0.72 , 1.41]
MDA 1.42 [0.86 , 2.33]
IARC GWAS 1.11 [0.98 , 1.27]
IARC Replication 1.08 [0.90 , 1.30]
Combined 1.10 [1.01 , 1.21]
Heterogeneity P−value= 0.8506
Overall 1.10 [1.05 , 1.15]
Heterogeneity P−value= 0.9807
0.00
0.50
1.00
1.50
2.00
2.50
Odds Ratio

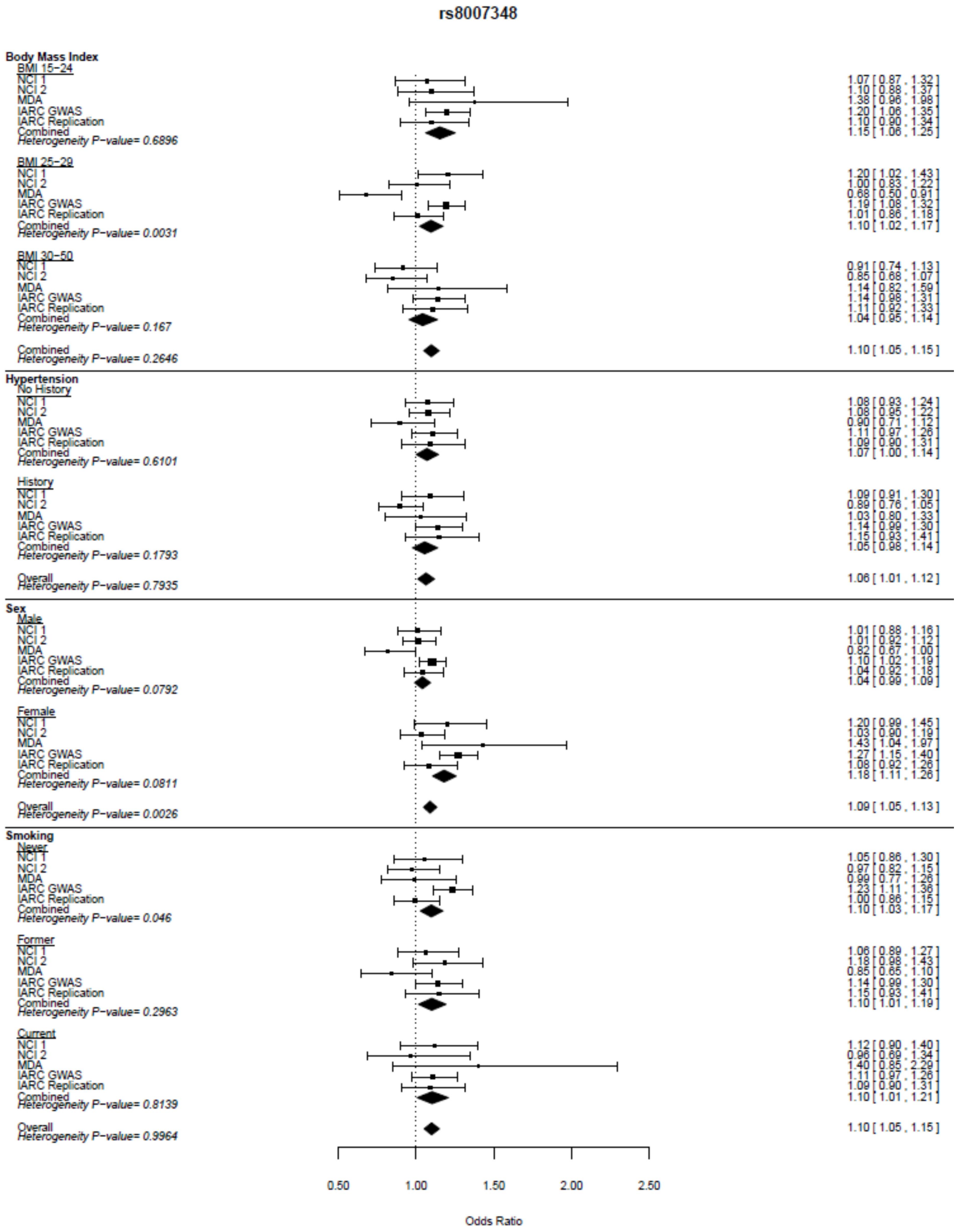

rs8007348
Body Mass Index
BMI 15−24
NCI 1
NCI 2
MDA
IARC GWAS
IARC Replication
Combined
Heterogeneity P−value= 0.6896
1.07 [0.87 , 1.32]
1.10 [0.88 , 1.37]
1.38 [0.96 , 1.98]
1.20 [1.06 , 1.35]
1.10 [0.90 , 1.34]
1.15 [1.06 , 1.25]
BMI 25−29
NCI 1
NCI 2
MDA
IARC GWAS
IARC Replication
Combined
Heterogeneity P−value= 0.0031
1.20 [1.02 , 1.43]
1.00 [0.83 , 1.22]
0.68 [0.50 , 0.91]
1.19 [1.08 , 1.32]
1.01 [0.86 , 1.18]
1.10 [1.02 , 1.17]
BMI 30−50
NCI 1
NCI 2
MDA
IARC GWAS
IARC Replication
Combined
Heterogeneity P−value= 0.167
0.91 [0.74 , 1.13]
0.85 [0.68 , 1.07]
1.14 [0.82 , 1.59]
1.14 [0.98 , 1.31]
1.11 [0.92 , 1.33]
1.04 [0.95 , 1.14]
Combined
Heterogeneity P−value= 0.2646
1.10 [1.05 , 1.15]
Hypertension
No History
NCI 1
NCI 2
MDA
IARC GWAS
IARC Replication
Combined
Heterogeneity P−value= 0.6101
1.08 [0.93 , 1.24]
1.08 [0.95 , 1.22]
0.90 [0.71 , 1.12]
1.11 [0.97 , 1.26]
1.09 [0.90 , 1.31]
1.07 [1.00 , 1.14]
History
NCI 1
NCI 2
MDA
IARC GWAS
IARC Replication
Combined
Heterogeneity P−value= 0.1793
1.09 [0.91 , 1.30]
0.89 [0.76 , 1.05]
1.03 [0.80 , 1.33]
1.14 [0.99 , 1.30]
1.15 [0.93 , 1.41]
1.05 [0.98 , 1.14]
Overall
Heterogeneity P−value= 0.7935
1.06 [1.01 , 1.12]
Sex
Male
NCI 1
NCI 2
MDA
IARC GWAS
IARC Replication
Combined
Heterogeneity P−value= 0.0792
1.01 [0.88 , 1.16]
1.01 [0.92 , 1.12]
0.82 [0.67 , 1.00]
1.10 [1.02 , 1.19]
1.04 [0.92 , 1.18]
1.04 [0.99 , 1.09]
Female
NCI 1
NCI 2
MDA
IARC GWAS
IARC Replication
Combined
Heterogeneity P−value= 0.0811
1.20 [0.99 , 1.45]
1.03 [0.90 , 1.19]
1.43 [1.04 , 1.97]
1.27 [1.15 , 1.40]
1.08 [0.92 , 1.26]
1.18 [1.11 , 1.26]
Overall
Heterogeneity P−value= 0.0026
1.09 [1.05 , 1.13]
Smoking
Never
NCI 1
NCI 2
MDA
IARC GWAS
IARC Replication
Combined
Heterogeneity P−value= 0.046
1.05 [0.86 , 1.30]
0.97 [0.82 , 1.15]
0.99 [0.77 , 1.26]
1.23 [1.11 , 1.36]
1.00 [0.86 , 1.15]
1.10 [1.03 , 1.17]
Former
NCI 1
NCI 2
MDA
IARC GWAS
IARC Replication
Combined
Heterogeneity P−value= 0.2963
1.06 [0.89 , 1.27]
1.18 [0.98 , 1.43]
0.85 [0.65 , 1.10]
1.14 [0.99 , 1.30]
1.15 [0.93 , 1.41]
1.10 [1.01 , 1.19]
Current
NCI 1
NCI 2
MDA
IARC GWAS
IARC Replication
Combined
Heterogeneity P−value= 0.8139
1.12 [0.90 , 1.40]
0.96 [0.69 , 1.34]
1.40 [0.85 , 2.29]
1.11 [0.97 , 1.26]
1.09 [0.90 , 1.31]
1.10 [1.01 , 1.21]
Overall
Heterogeneity P−value= 0.9964
1.10 [1.05 , 1.15]
0.50
1.00
1.50
2.00
2.50
Odds Ratio

rs4903064

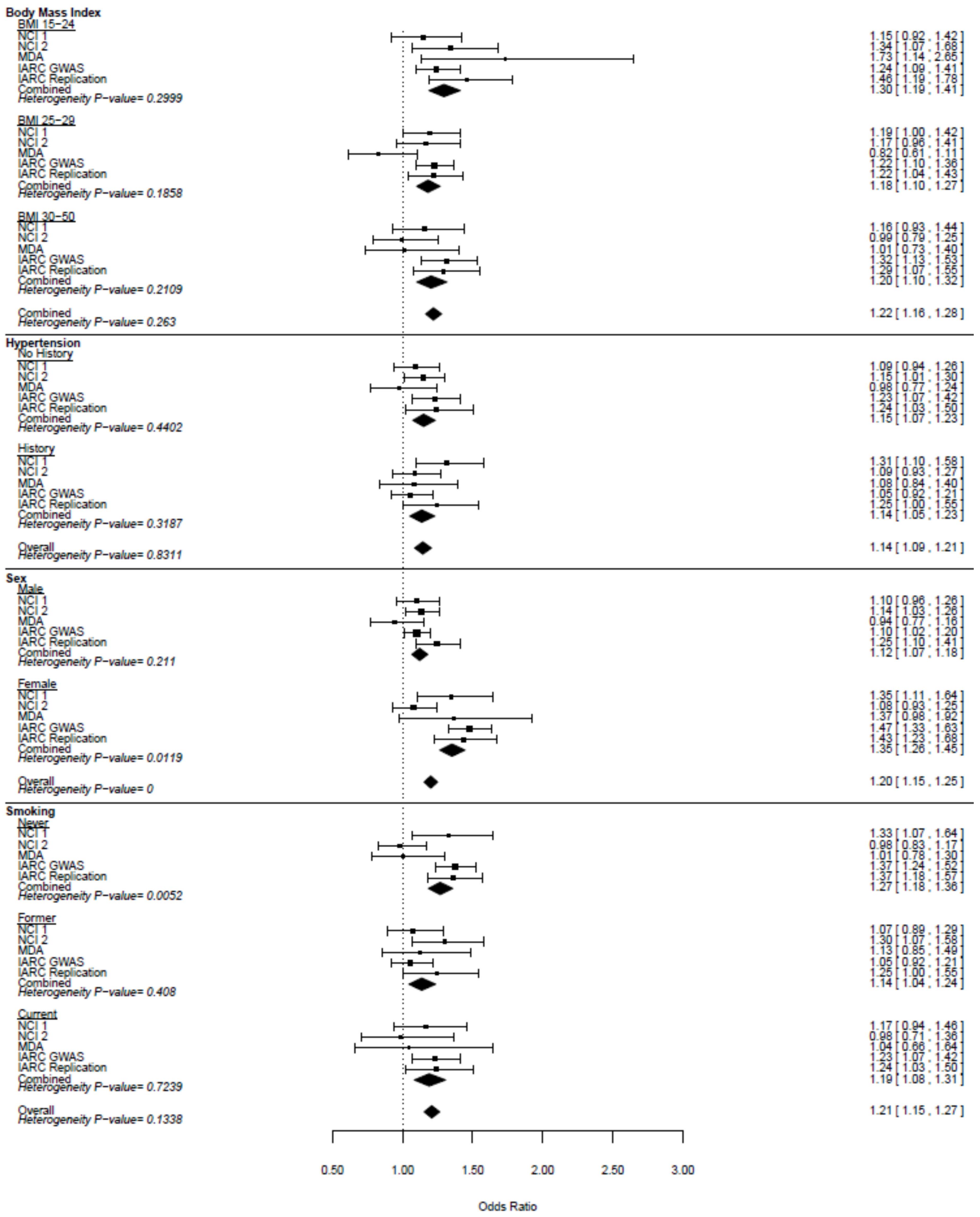
Body Mass Index
BMI 15−24
NCI 1 1.15 [0.92 , 1.42]
NCI 2 1.34 [1.07 , 1.68]
MDA 1.73 [1.14 , 2.65]
IARC GWAS 1.24 [1.09 , 1.41]
IARC Replication 1.46 [1.19 , 1.78]
Combined 1.30 [1.19 , 1.41]
Heterogeneity P−value= 0.2999
BMI 25−29
NCI 1 1.19 [1.00 , 1.42]
NCI 2 1.17 [0.96 , 1.41]
MDA 0.82 [0.61 , 1.11]
IARC GWAS 1.22 [1.10 , 1.36]
IARC Replication 1.22 [1.04 , 1.43]
Combined 1.18 [1.10 , 1.27]
Heterogeneity P−value= 0.1858
BMI 30−50
NCI 1 1.16 [0.93 , 1.44]
NCI 2 0.99 [0.79 , 1.25]
MDA 1.01 [0.73 , 1.40]
IARC GWAS 1.32 [1.13 , 1.53]
IARC Replication 1.29 [1.07 , 1.55]
Combined 1.20 [1.10 , 1.32]
Heterogeneity P−value= 0.2109
Combined 1.22 [1.16 , 1.28]
Heterogeneity P−value= 0.263
Hypertension
No History
NCI 1 1.09 [0.94 , 1.26]
NCI 2 1.15 [1.01 , 1.30]
MDA 0.98 [0.77 , 1.24]
IARC GWAS 1.23 [1.07 , 1.42]
IARC Replication 1.24 [1.03 , 1.50]
Combined 1.15 [1.07 , 1.23]
Heterogeneity P−value= 0.4402
History
NCI 1 1.31 [1.10 , 1.58]
NCI 2 1.09 [0.93 , 1.27]
MDA 1.08 [0.84 , 1.40]
IARC GWAS 1.05 [0.92 , 1.21]
IARC Replication 1.25 [1.00 , 1.55]
Combined 1.14 [1.05 , 1.23]
Heterogeneity P−value= 0.3187
Overall 1.14 [1.09 , 1.21]
Heterogeneity P−value= 0.8311
Sex
Male
NCI 1 1.10 [0.96 , 1.26]
NCI 2 1.14 [1.03 , 1.26]
MDA 0.94 [0.77 , 1.16]
IARC GWAS 1.10 [1.02 , 1.20]
IARC Replication 1.25 [1.10 , 1.41]
Combined 1.12 [1.07 , 1.18]
Heterogeneity P−value= 0.211
Female
NCI 1 1.35 [1.11 , 1.64]
NCI 2 1.08 [0.93 , 1.25]
MDA 1.37 [0.98 , 1.92]
IARC GWAS 1.47 [1.33 , 1.63]
IARC Replication 1.43 [1.23 , 1.68]
Combined 1.35 [1.26 , 1.45]
Heterogeneity P−value= 0.0119
Overall 1.20 [1.15 , 1.25]
Heterogeneity P−value= 0
Smoking
Never
NCI 1 1.33 [1.07 , 1.64]
NCI 2 0.98 [0.83 , 1.17]
MDA 1.01 [0.78 , 1.30]
IARC GWAS 1.37 [1.24 , 1.52]
IARC Replication 1.37 [1.18 , 1.57]
Combined 1.27 [1.18 , 1.36]
Heterogeneity P−value= 0.0052
Former
NCI 1 1.07 [0.89 , 1.29]
NCI 2 1.30 [1.07 , 1.58]
MDA 1.13 [0.85 , 1.49]
IARC GWAS 1.05 [0.92 , 1.21]
IARC Replication 1.25 [1.00 , 1.55]
Combined 1.14 [1.04 , 1.24]
Heterogeneity P−value= 0.408
Current
NCI 1 1.17 [0.94 , 1.46]
NCI 2 0.98 [0.71 , 1.36]
MDA 1.04 [0.66 , 1.64]
IARC GWAS 1.23 [1.07 , 1.42]
IARC Replication 1.24 [1.03 , 1.50]
Combined 1.19 [1.08 , 1.31]
Heterogeneity P−value= 0.7239
Overall 1.21 [1.15 , 1.27]
Heterogeneity P−value= 0.1338
0.50
1.00
1.50
2.00
2.50
3.00
Odds Ratio

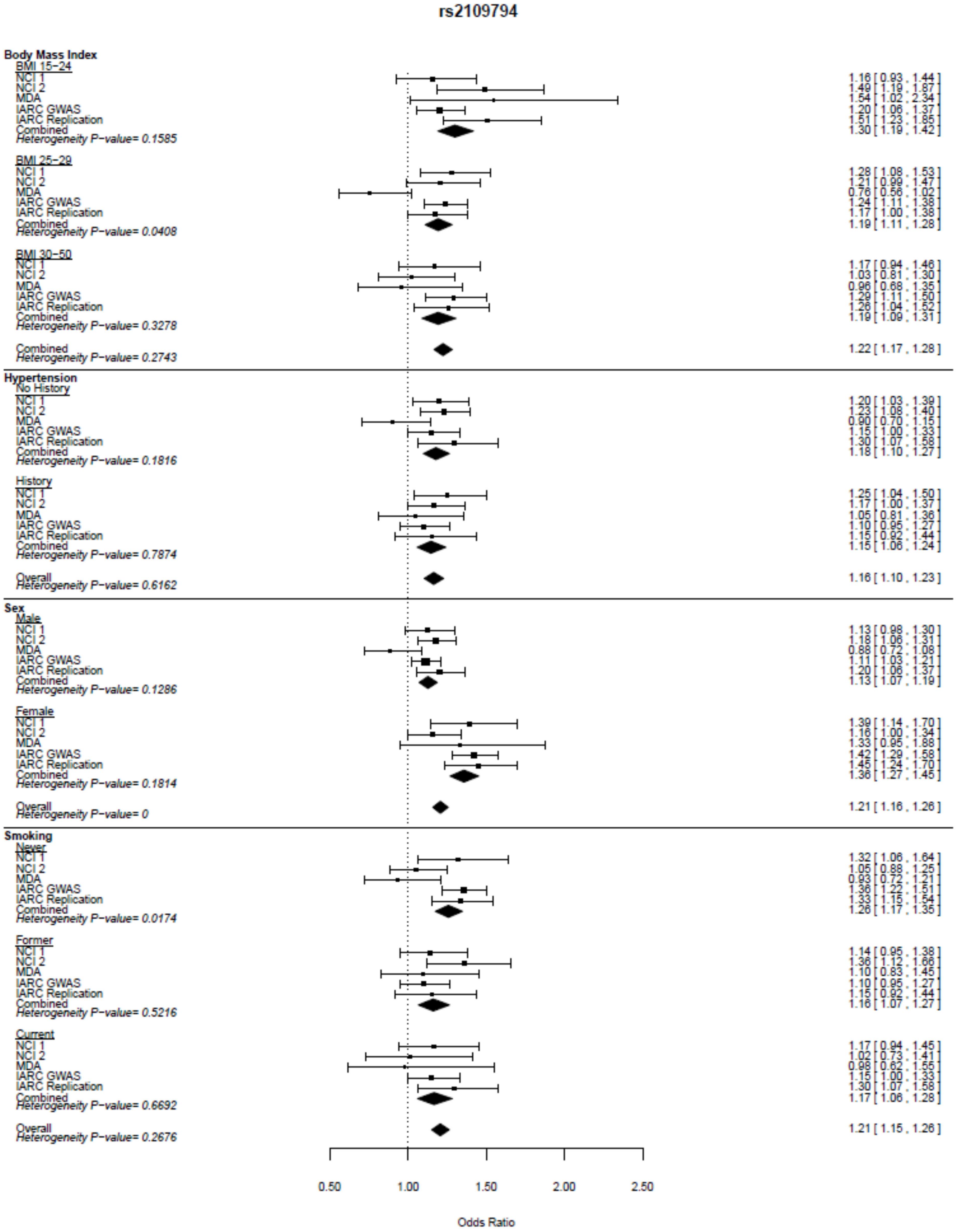
rs2109794
Body Mass Index
BMI 15–24
NCI 1
NCI 2
MDA
IARC GWAS
IARC Replication
Combined
Heterogeneity P–value= 0.1585
BMI 25–29
NCI 1
NCI 2
MDA
IARC GWAS
IARC Replication
Combined
Heterogeneity P–value= 0.0408
BMI 30–50
NCI 1
NCI 2
MDA
IARC GWAS
IARC Replication
Combined
Heterogeneity P–value= 0.3278
Combined
Heterogeneity P–value= 0.2743
Hypertension
No History
NCI 1
NCI 2
MDA
IARC GWAS
IARC Replication
Combined
Heterogeneity P–value= 0.1816
History
NCI 1
NCI 2
MDA
IARC GWAS
IARC Replication
Combined
Heterogeneity P–value= 0.7874
Overall
Heterogeneity P–value= 0.6162
Sex
Male
NCI 1
NCI 2
MDA
IARC GWAS
IARC Replication
Combined
Heterogeneity P–value= 0.1286
Female
NCI 1
NCI 2
MDA
IARC GWAS
IARC Replication
Combined
Heterogeneity P–value= 0.1814
Overall
Heterogeneity P–value= 0
Smoking
Never
NCI 1
NCI 2
MDA
IARC GWAS
IARC Replication
Combined
Heterogeneity P–value= 0.0174
Former
NCI 1
NCI 2
MDA
IARC GWAS
IARC Replication
Combined
Heterogeneity P–value= 0.5216
Current
NCI 1
NCI 2
MDA
IARC GWAS
IARC Replication
Combined
Heterogeneity P–value= 0.6692
Overall
Heterogeneity P–value= 0.2676
1.16 [0.93 , 1.44]
1.49 [1.19 , 1.87]
1.54 [1.02 , 2.34]
1.20 [1.06 , 1.37]
1.51 [1.23 , 1.85]
1.30 [1.19 , 1.42]
1.28 [1.08 , 1.53]
1.21 [0.99 , 1.47]
0.76 [0.56 , 1.02]
1.24 [1.11 , 1.38]
1.17 [1.00 , 1.38]
1.19 [1.11 , 1.28]
1.17 [0.94 , 1.46]
1.03 [0.81 , 1.30]
0.96 [0.68 , 1.35]
1.29 [1.11 , 1.50]
1.26 [1.04 , 1.52]
1.19 [1.09 , 1.31]
1.22 [1.17 , 1.28]
1.20 [1.03 , 1.39]
1.23 [1.08 , 1.40]
0.90 [0.70 , 1.15]
1.15 [1.00 , 1.33]
1.30 [1.07 , 1.58]
1.18 [1.10 , 1.27]
1.25 [1.04 , 1.50]
1.17 [1.00 , 1.37]
1.05 [0.81 , 1.36]
1.10 [0.95 , 1.27]
1.15 [0.92 , 1.44]
1.15 [1.06 , 1.24]
1.16 [1.10 , 1.23]
1.13 [0.98 , 1.30]
1.18 [1.06 , 1.31]
0.88 [0.72 , 1.08]
1.11 [1.03 , 1.21]
1.20 [1.06 , 1.37]
1.13 [1.07 , 1.19]
1.39 [1.14 , 1.70]
1.16 [1.00 , 1.34]
1.33 [0.95 , 1.88]
1.42 [1.29 , 1.58]
1.45 [1.24 , 1.70]
1.36 [1.27 , 1.45]
1.21 [1.16 , 1.26]
1.32 [1.06 , 1.64]
1.05 [0.88 , 1.25]
0.93 [0.72 , 1.21]
1.36 [1.22 , 1.51]
1.33 [1.15 , 1.54]
1.26 [1.17 , 1.35]
1.14 [0.95 , 1.38]
1.36 [1.12 , 1.66]
1.10 [0.83 , 1.45]
1.10 [0.95 , 1.27]
1.15 [0.92 , 1.44]
1.16 [1.07 , 1.27]
1.17 [0.94 , 1.45]
1.02 [0.73 , 1.41]
0.98 [0.62 , 1.55]
1.15 [1.00 , 1.33]
1.30 [1.07 , 1.58]
1.17 [1.06 , 1.28]
1.21 [1.15 , 1.26]
0.50
1.00
1.50
2.00
2.50
Odds Ratio

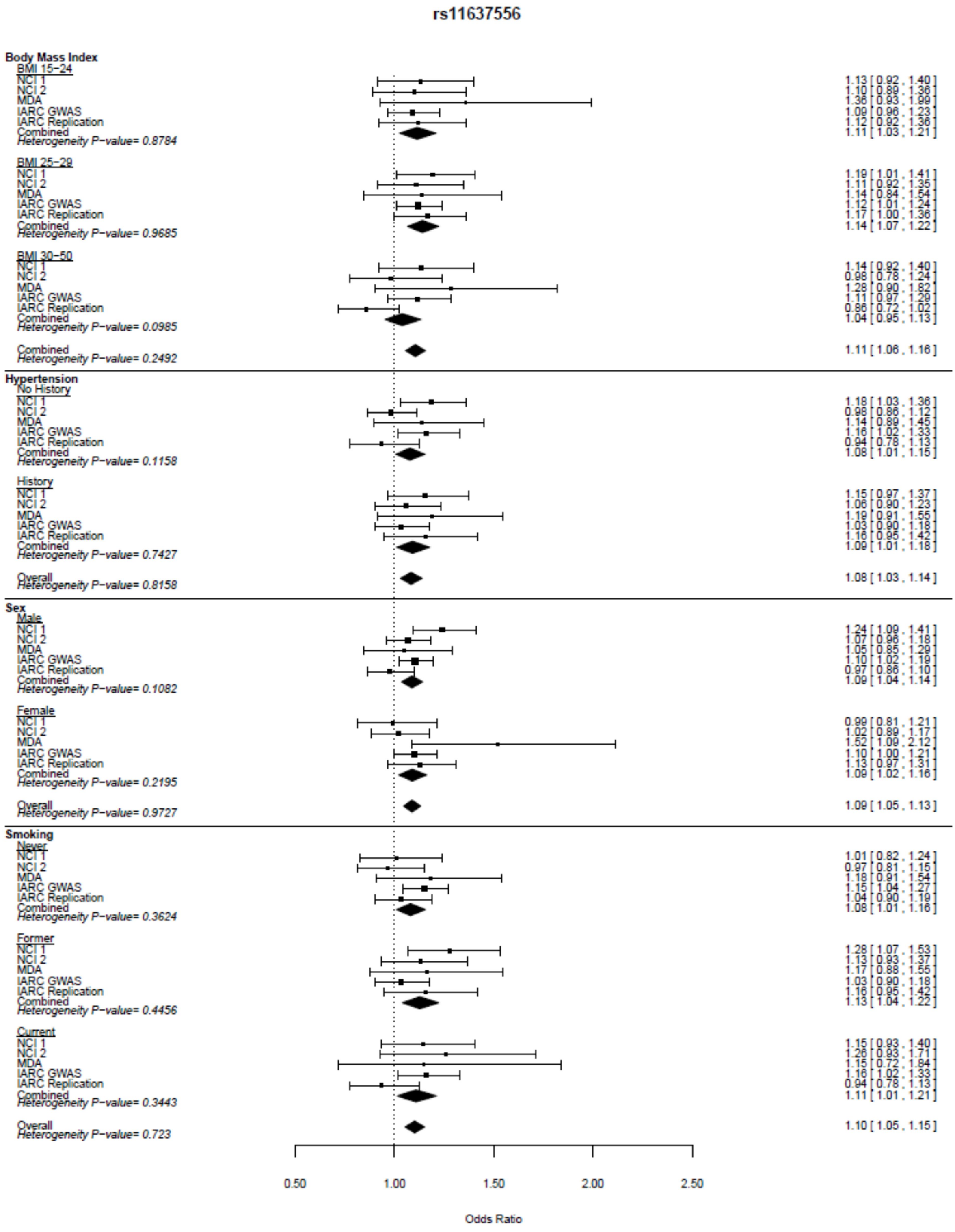
rs11637556
Body Mass Index
BMI 15−24
NCI 1 1.13 [0.92 , 1.40]
NCI 2 1.10 [0.89 , 1.36]
MDA 1.36 [0.93 , 1.99]
IARC GWAS 1.09 [0.96 , 1.23]
IARC Replication 1.12 [0.92 , 1.36]
Combined 1.11 [1.03 , 1.21]
Heterogeneity P−value= 0.8784
BMI 25−29
NCI 1 1.19 [1.01 , 1.41]
NCI 2 1.11 [0.92 , 1.35]
MDA 1.14 [0.84 , 1.54]
IARC GWAS 1.12 [1.01 , 1.24]
IARC Replication 1.17 [1.00 , 1.36]
Combined 1.14 [1.07 , 1.22]
Heterogeneity P−value= 0.9685
BMI 30−50
NCI 1 1.14 [0.92 , 1.40]
NCI 2 0.98 [0.78 , 1.24]
MDA 1.28 [0.90 , 1.82]
IARC GWAS 1.11 [0.97 , 1.29]
IARC Replication 0.86 [0.72 , 1.02]
Combined 1.04 [0.95 , 1.13]
Heterogeneity P−value= 0.0985
Combined 1.11 [1.06 , 1.16]
Heterogeneity P−value= 0.2492
Hypertension
No History
NCI 1 1.18 [1.03 , 1.36]
NCI 2 0.98 [0.86 , 1.12]
MDA 1.14 [0.89 , 1.45]
IARC GWAS 1.16 [1.02 , 1.33]
IARC Replication 0.94 [0.78 , 1.13]
Combined 1.08 [1.01 , 1.15]
Heterogeneity P−value= 0.1158
History
NCI 1 1.15 [0.97 , 1.37]
NCI 2 1.06 [0.90 , 1.23]
MDA 1.19 [0.91 , 1.55]
IARC GWAS 1.03 [0.90 , 1.18]
IARC Replication 1.16 [0.95 , 1.42]
Combined 1.09 [1.01 , 1.18]
Heterogeneity P−value= 0.7427
Overall 1.08 [1.03 , 1.14]
Heterogeneity P−value= 0.8158
Sex
Male
NCI 1 1.24 [1.09 , 1.41]
NCI 2 1.07 [0.96 , 1.18]
MDA 1.05 [0.85 , 1.29]
IARC GWAS 1.10 [1.02 , 1.19]
IARC Replication 0.97 [0.86 , 1.10]
Combined 1.09 [1.04 , 1.14]
Heterogeneity P−value= 0.1082
Female
NCI 1 0.99 [0.81 , 1.21]
NCI 2 1.02 [0.89 , 1.17]
MDA 1.52 [1.09 , 2.12]
IARC GWAS 1.10 [1.00 , 1.21]
IARC Replication 1.13 [0.97 , 1.31]
Combined 1.09 [1.02 , 1.16]
Heterogeneity P−value= 0.2195
Overall 1.09 [1.05 , 1.13]
Heterogeneity P−value= 0.9727
Smoking
Never
NCI 1 1.01 [0.82 , 1.24]
NCI 2 0.97 [0.81 , 1.15]
MDA 1.18 [0.91 , 1.54]
IARC GWAS 1.15 [1.04 , 1.27]
IARC Replication 1.04 [0.90 , 1.19]
Combined 1.08 [1.01 , 1.16]
Heterogeneity P−value= 0.3624
Former
NCI 1 1.28 [1.07 , 1.53]
NCI 2 1.13 [0.93 , 1.37]
MDA 1.17 [0.88 , 1.55]
IARC GWAS 1.03 [0.90 , 1.18]
IARC Replication 1.16 [0.95 , 1.42]
Combined 1.13 [1.04 , 1.22]
Heterogeneity P−value= 0.4456
Current
NCI 1 1.15 [0.93 , 1.40]
NCI 2 1.26 [0.93 , 1.71]
MDA 1.15 [0.72 , 1.84]
IARC GWAS 1.16 [1.02 , 1.33]
IARC Replication 0.94 [0.78 , 1.13]
Combined 1.11 [1.01 , 1.21]
Heterogeneity P−value= 0.3443
Overall 1.10 [1.05 , 1.15]
Heterogeneity P−value= 0.723
0.50
1.00
1.50
2.00
2.50
Odds Ratio

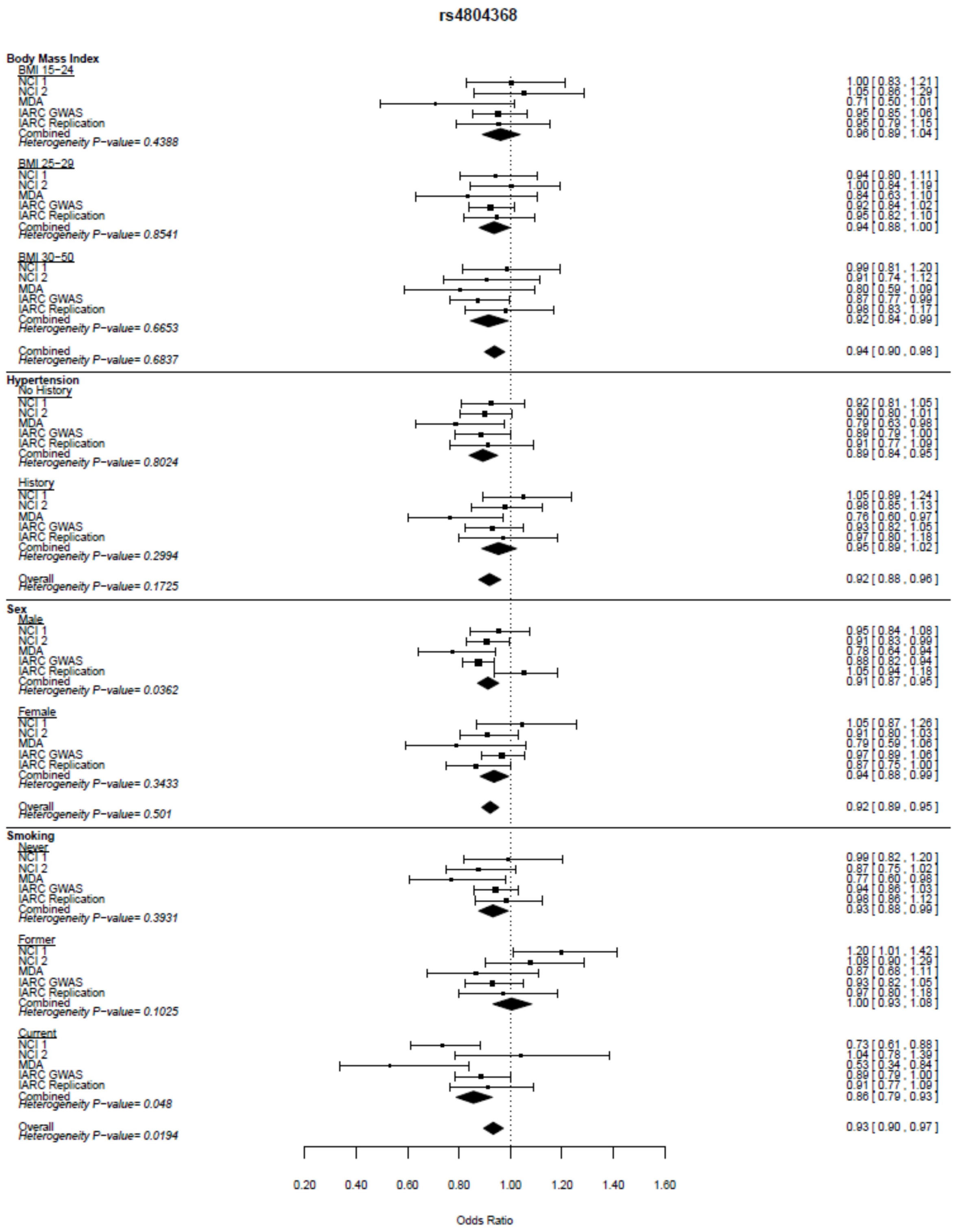
rs4804368
Body Mass Index
BMI 15-24
NCI 1
NCI 2
MDA
IARC GWAS
IARC Replication
Combined
Heterogeneity P-value= 0.4388
1.00 [0.83 , 1.21]
1.05 [0.86 , 1.29]
0.71 [0.50 , 1.01]
0.95 [0.85 , 1.06]
0.95 [0.79 , 1.15]
0.96 [0.89 , 1.04]
BMI 25-29
NCI 1
NCI 2
MDA
IARC GWAS
IARC Replication
Combined
Heterogeneity P-value= 0.8541
0.94 [0.80 , 1.11]
1.00 [0.84 , 1.19]
0.84 [0.63 , 1.10]
0.92 [0.84 , 1.02]
0.95 [0.82 , 1.10]
0.94 [0.88 , 1.00]
BMI 30-50
NCI 1
NCI 2
MDA
IARC GWAS
IARC Replication
Combined
Heterogeneity P-value= 0.6653
0.99 [0.81 , 1.20]
0.91 [0.74 , 1.12]
0.80 [0.59 , 1.09]
0.87 [0.77 , 0.99]
0.98 [0.83 , 1.17]
0.92 [0.84 , 0.99]
Combined
Heterogeneity P-value= 0.6837
0.94 [0.90 , 0.98]
Hypertension
No History
NCI 1
NCI 2
MDA
IARC GWAS
IARC Replication
Combined
Heterogeneity P-value= 0.8024
0.92 [0.81 , 1.05]
0.90 [0.80 , 1.01]
0.79 [0.63 , 0.98]
0.89 [0.79 , 1.00]
0.91 [0.77 , 1.09]
0.89 [0.84 , 0.95]
History
NCI 1
NCI 2
MDA
IARC GWAS
IARC Replication
Combined
Heterogeneity P-value= 0.2994
1.05 [0.89 , 1.24]
0.98 [0.85 , 1.13]
0.76 [0.60 , 0.97]
0.93 [0.82 , 1.05]
0.97 [0.80 , 1.18]
0.95 [0.89 , 1.02]
Overall
Heterogeneity P-value= 0.1725
0.92 [0.88 , 0.96]
Sex
Male
NCI 1
NCI 2
MDA
IARC GWAS
IARC Replication
Combined
Heterogeneity P-value= 0.0362
0.95 [0.84 , 1.08]
0.91 [0.83 , 0.99]
0.78 [0.64 , 0.94]
0.88 [0.82 , 0.94]
1.05 [0.94 , 1.18]
0.91 [0.87 , 0.95]
Female
NCI 1
NCI 2
MDA
IARC GWAS
IARC Replication
Combined
Heterogeneity P-value= 0.3433
1.05 [0.87 , 1.26]
0.91 [0.80 , 1.03]
0.79 [0.59 , 1.06]
0.97 [0.89 , 1.06]
0.87 [0.75 , 1.00]
0.94 [0.88 , 0.99]
Overall
Heterogeneity P-value= 0.501
0.92 [0.89 , 0.95]
Smoking
Never
NCI 1
NCI 2
MDA
IARC GWAS
IARC Replication
Combined
Heterogeneity P-value= 0.3931
0.99 [0.82 , 1.20]
0.87 [0.75 , 1.02]
0.77 [0.60 , 0.98]
0.94 [0.86 , 1.03]
0.98 [0.86 , 1.12]
0.93 [0.88 , 0.99]
Former
NCI 1
NCI 2
MDA
IARC GWAS
IARC Replication
Combined
Heterogeneity P-value= 0.1025
1.20 [1.01 , 1.42]
1.08 [0.90 , 1.29]
0.87 [0.68 , 1.11]
0.93 [0.82 , 1.05]
0.97 [0.80 , 1.18]
1.00 [0.93 , 1.08]
Current
NCI 1
NCI 2
MDA
IARC GWAS
IARC Replication
Combined
Heterogeneity P-value= 0.048
0.73 [0.61 , 0.88]
1.04 [0.78 , 1.39]
0.53 [0.34 , 0.84]
0.89 [0.79 , 1.00]
0.91 [0.77 , 1.09]
0.86 [0.79 , 0.93]
Overall
Heterogeneity P-value= 0.0194
0.93 [0.90 , 0.97]
0.20
0.40
0.60
0.80
1.00
1.20
1.40
1.60
Odds Ratio

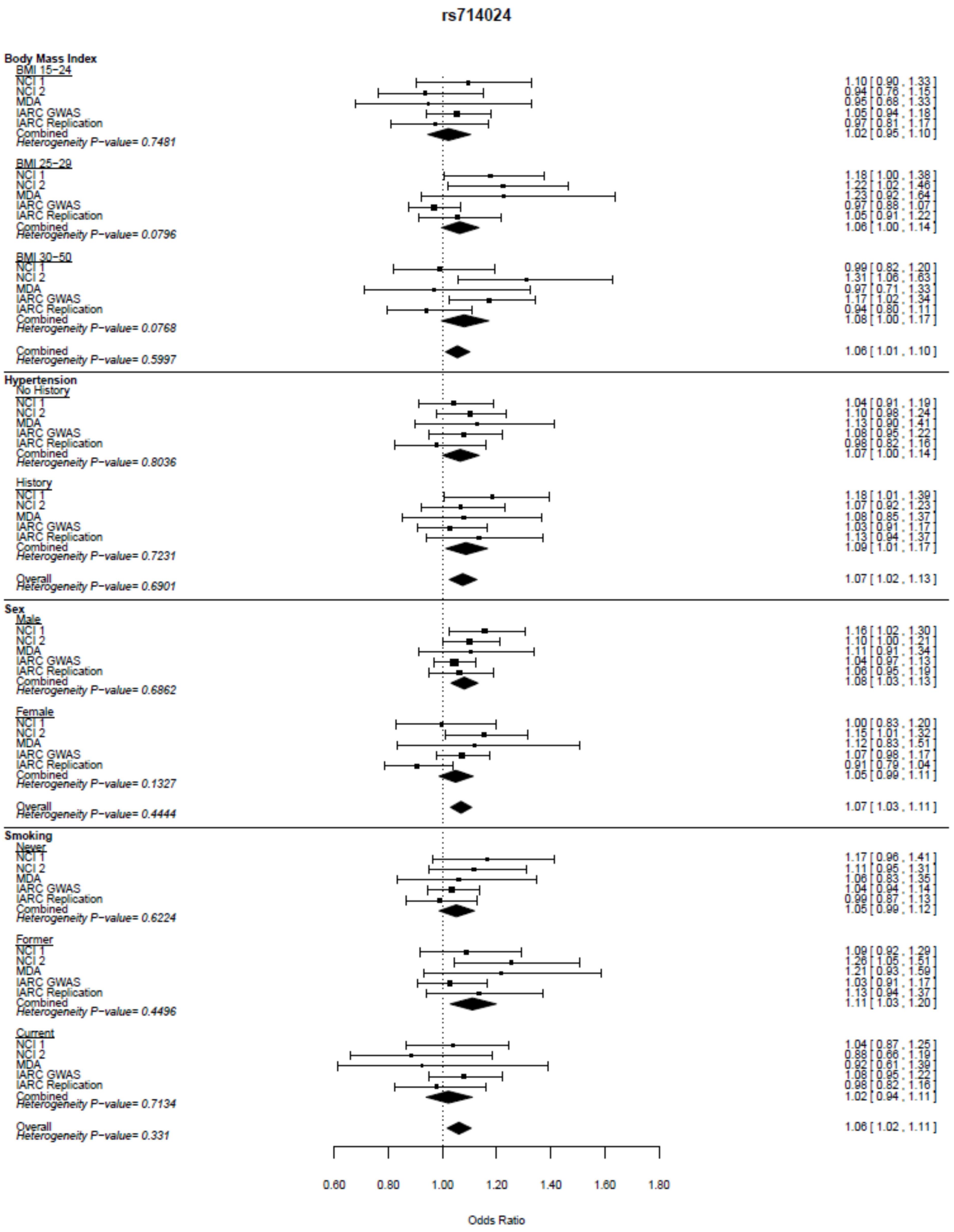
rs714024
Body Mass Index
BMI 15−24
NCI 1 1.10 [0.90 , 1.33]
NCI 2 0.94 [0.76 , 1.15]
MDA 0.95 [0.68 , 1.33]
IARC GWAS 1.05 [0.94 , 1.18]
IARC Replication 0.97 [0.81 , 1.17]
Combined 1.02 [0.95 , 1.10]
Heterogeneity P−value= 0.7481
BMI 25−29
NCI 1 1.18 [1.00 , 1.38]
NCI 2 1.22 [1.02 , 1.46]
MDA 1.23 [0.92 , 1.64]
IARC GWAS 0.97 [0.88 , 1.07]
IARC Replication 1.05 [0.91 , 1.22]
Combined 1.06 [1.00 , 1.14]
Heterogeneity P−value= 0.0796
BMI 30−50
NCI 1 0.99 [0.82 , 1.20]
NCI 2 1.31 [1.06 , 1.63]
MDA 0.97 [0.71 , 1.33]
IARC GWAS 1.17 [1.02 , 1.34]
IARC Replication 0.94 [0.80 , 1.11]
Combined 1.08 [1.00 , 1.17]
Heterogeneity P−value= 0.0768
Combined 1.06 [1.01 , 1.10]
Heterogeneity P−value= 0.5997
Hypertension
No History
NCI 1 1.04 [0.91 , 1.19]
NCI 2 1.10 [0.98 , 1.24]
MDA 1.13 [0.90 , 1.41]
IARC GWAS 1.08 [0.95 , 1.22]
IARC Replication 0.98 [0.82 , 1.16]
Combined 1.07 [1.00 , 1.14]
Heterogeneity P−value= 0.8036
History
NCI 1 1.18 [1.01 , 1.39]
NCI 2 1.07 [0.92 , 1.23]
MDA 1.08 [0.85 , 1.37]
IARC GWAS 1.03 [0.91 , 1.17]
IARC Replication 1.13 [0.94 , 1.37]
Combined 1.09 [1.01 , 1.17]
Heterogeneity P−value= 0.7231
Overall 1.07 [1.02 , 1.13]
Heterogeneity P−value= 0.6901
Sex
Male
NCI 1 1.16 [1.02 , 1.30]
NCI 2 1.10 [1.00 , 1.21]
MDA 1.11 [0.91 , 1.34]
IARC GWAS 1.04 [0.97 , 1.13]
IARC Replication 1.06 [0.95 , 1.19]
Combined 1.08 [1.03 , 1.13]
Heterogeneity P−value= 0.6862
Female
NCI 1 1.00 [0.83 , 1.20]
NCI 2 1.15 [1.01 , 1.32]
MDA 1.12 [0.83 , 1.51]
IARC GWAS 1.07 [0.98 , 1.17]
IARC Replication 0.91 [0.79 , 1.04]
Combined 1.05 [0.99 , 1.11]
Heterogeneity P−value= 0.1327
Overall 1.07 [1.03 , 1.11]
Heterogeneity P−value= 0.4444
Smoking
Never
NCI 1 1.17 [0.96 , 1.41]
NCI 2 1.11 [0.95 , 1.31]
MDA 1.06 [0.83 , 1.35]
IARC GWAS 1.04 [0.94 , 1.14]
IARC Replication 0.99 [0.87 , 1.13]
Combined 1.05 [0.99 , 1.12]
Heterogeneity P−value= 0.6224
Former
NCI 1 1.09 [0.92 , 1.29]
NCI 2 1.26 [1.05 , 1.51]
MDA 1.21 [0.93 , 1.59]
IARC GWAS 1.03 [0.91 , 1.17]
IARC Replication 1.13 [0.94 , 1.37]
Combined 1.11 [1.03 , 1.20]
Heterogeneity P−value= 0.4496
Current
NCI 1 1.04 [0.87 , 1.25]
NCI 2 0.88 [0.66 , 1.19]
MDA 0.92 [0.61 , 1.39]
IARC GWAS 1.08 [0.95 , 1.22]
IARC Replication 0.98 [0.82 , 1.16]
Combined 1.02 [0.94 , 1.11]
Heterogeneity P−value= 0.7134
Overall 1.06 [1.02 , 1.11]
Heterogeneity P−value= 0.331
0.60
0.80
1.00
1.20
1.40
1.60
1.80
Odds Ratio

Supplementary Tables.

Supplementary Table 1. Description of the studies participating in the RCC GWAS meta-analysis.

			Genotyped Samples		Samples Used in Final Analysis		Genotyping Array	
**Study Name (Abbreviation)**	**Design; Location**	**Study Population**	**Cases**	**Controls**	**Cases**	**Controls**	**Cases**	**Controls**
**NEW SAMPLES**			**5,722**	**7,439**	**5,198**	**7,331**		
**IARC-2 scan**								
The European Prospective Investigation into Cancer and Nutrition (EPIC)	Prospective cohort 10 European countries	Adults aged 35-74 enrolled between 1992 and 2000 (n=521,468) Incident cancers identified through health insurance records, cancer and pathology registries and active follow-up (subjects and next of skin) Genomic DNA extracted from buffy coat	127	138	127	138	Omni 5	Omni 5
CeRePP	Case series France	Pathologically confirmed RCC recruited through the CeRePP network. Controls recruited from a systematic urologic screening program, which includes men or women who had no history or symptoms of kidney. diseases and with a normal morphologic status of kidneys at ultrasonographic examination. Genomic DNA extracted from blood and saliva samples.	411	429	402	422	Omni 5	Omni 5
Umea	Prospective cohort Sweden	Population-based cohort, recruiting since 1985; resident from the Västerbotten county in northern Sweden. Incident cases identified through linkage to national death and cancer registries. Genomic DNA extracted from blood.	306	312	297	301	Omni 5	Omni 5
Karolinska	2 Prospective cohorts Sweden	COSM: Cohort of Swedish Men (45,306 men enrolled in 1997) and SMC: Swedish Mammography Cohort (61,433 women enrolled between 1987 and 1990), in central Sweden. Incident cases identified through linkage to national death and cancer registries. Genomic DNA extracted from saliva.	131	298	131	295	Omni 5	Omni 5
Consortium For the Investigation of Renal Malignancies/ Melbourne Collaborative Cohort Study (ConFIRM/MCCS)	Case-control and cohort studies; Australia	Incident cases diagnosed in Victoria and Queensland in the period 2011-2014 and aged 18-74 years (ConFIRM), and from within the MCCS when fresh blood collected; Controls selected from healthy participants in the MCCS cohort; Genomic DNA extracted from whole blood (ConFIRM) and buffy coat (MCCS).	188	398	184	396	Omni 5	Omni 5
The IARC K2 study	Case-control study 4 central/eastern Europe countries	Multicentric study conducted in 2007-2013 in Russia, Czech Republic, Romania, and Serbia. Hospital or population-based controls depending on recruiting centers.	1,675	1,458	1,640	1,388	Omni 5	Omni 5/ Omni Express
**IARC-2 Scan Total**			**2,838**	**3,033**	**2,781**	**2,940**		
**NCI-2 Scan:**								
Agricultural Health Study (AHS)	Prospective cohort; USA (Iowa, North Carolina)	Private and commercial pesticide applicators and spouses of private applicators, enrolled between 1993 and 1997 (n = 89,655). Incident cancers identified through linkage with state cancer registries. Germline DNA extracted from buccal cell samples.	78	-	7	-	Omni Express	-
Alpha-Tocopherol, Beta-Carotene Cancer Prevention Study (ATBC)	Prospective cohort / randomized trial; Finland	Male smokers aged 50-69 at entry, enrolled between 1985 and 1988 (n = 29,133). Incident cancers identified through linkage to Finnish Cancer Registry. Germline DNA extracted from whole blood samples.	41	221*	39	221*	Omni Express	Omni 2.5

BioVU	Hospital biorepository; USA (Nashville)	Renal cancer patients treated at Vanderbilt University Medical Center between 2006 and 2012. Controls selected from among patients admitted for non-cancer condition, frequency matched by age and sex.	714	222	629	207	Omni Express	Omni Express
American Cancer Society Cancer Prevention Study II Nutrition Cohort (CPS-II)	Prospective cohort; USA	Men and women aged 50-74 at entry, enrolled between 1992 and 1993 (n = 184,194). Incident cancers identified from biannual follow-up questionnaires or linkage with state cancer registries. Germline DNA extracted from blood and buccal cell samples.	85	212*	70	212*	Omni Express	Omni 2.5
Dana-Farber/Harvard Cancer Center (DFHCC)	Case series; USA (Boston)	Patients with localized renal cancer treated at DFHCC between 2002 and 2010. Genomic DNA extracted from blood samples.	234	-	206	-	Omni Express	-
Health Professionals Follow-up Study (HPFS)	Prospective cohort; USA	Men in selected health professions, enrolled in 1986 (n = 51,529). Incident cancers identified from follow-up questionnaires and subsequent medical record review. Germline DNA extracted from blood samples.	50	85*	38	85*	Omni Express	Omni Express
Nurses' Health Study (NHS)	Prospective cohort; USA	Female registered nurses aged 30-55, enrolled in 1976 (n = 121,700). Incident cancers identified by mailed follow-up questionnaire and subsequent medical record review. Germline DNA extracted from blood samples.	66	434*	57	434*	Omni Express	Omni Express
Physicians Health Study (PHS)	Prospective cohort / randomized trial; USA	Male physicians aged 50+ enrolled between 1982 and 1984 (n = 22,071). Incident cancers identified by mailed follow-up questionnaire. Germline DNA extracted from blood samples.	27	-	21	-	Omni Express	-
Prostate, Lung, Colorectal and Ovarian Cancer Screening Trial (PLCO)	Prospective cohort / cancer screening trial; USA	Men and women aged 55-74 at entry, enrolled between 1993 and 2001 (n = 155,000). Incident cancers identified from annual questionnaires, with confirmation through medical records. Germline DNA extracted from buffy coat and buccal cell samples.	118	3,003*	93	3,003*	Omni Express	Omni 2.5
Van Andel Research Institute (VARI)	Case series; USA	Renal cancer patients treated at the Spectrum Health Hospital in Grand Rapids, MI, and in hospitals affiliated with the Cooperative Human Tissue Network (CHTN). Germline DNA extracted from adjacent normal kidney tissue.	1,050	-	920	-	Omni Express	-
Vitamins and Lifestyle Study (VITAL)	Prospective cohort; USA (Washington State)	Men and women aged 50-76, enrolled between 2000 and 2002. Incident cancers identified by linkage to the western Washington SEER cancer registry. Germline DNA extracted from buccal cell samples.	112	-	91	-	Omni Express	-
Women's Health Initiative (WHI)	Prospective cohort / randomized trial; USA	Women aged 50-79, enrolled between 1993 and 1998. Incident cancers were identified by mailed follow-up questionnaires, and verified by medical records. Genomic DNA extracted from buffy coat samples.	300	229*	239	229*	Omni Express	Omni Express
Women's Health Study (WHS)	Prospective cohort / randomized trial; USA	Female health professionals aged 45 years or older, enrolled in 1993. Incident cancers identified by mailed follow-up questionnaire. Genomic DNA extracted from blood samples.	9	-	7	-	Omni Express	-
**NCI-2 Scan Total**			**2,884**	**4,406**	**2,417**	**4,391**		
**PREVIOUSLY AVAILABLE SAMPLES**			**6,031**	**13,436**	**5,585**	**13,075**		
**IARC-1 scan**								
The European Prospective Investigation into Cancer and Nutrition (EPIC) [additional to above]	Prospective cohort 10 European countries	Adults aged 35-74 enrolled between 1992 and 2000 (n=521,468) Incident cancers identified through health insurance records, cancer and pathology registries and active follow-up (subjects and next of skin) Genomic DNA extracted from buffy coat	319	434	275	414	610Q	550K

The Nord-Trøndelag Health (HUNT2) and Tromsø Studies (Tromsø IV)	2 Prospective cohorts<br>Norway	HUNT2: Adults aged 20+ enrolled between 1995 and 1997 (N=65,285)<br>Tromsø IV: Adults aged 25+ enrolled between 1994-1995 (N=27,158)<br>Cases identified through Norwegian Cancer Registries<br>Genomic DNA extracted from buffy coat	145	433	133	388	610Q	317K
The NCI/IARC study in central Europe (CE)	Case-control study<br>4 European countries	Hospital based study conducted between 1999-2003 (except Poland) 2004-2007 (Poland). Controls matched to lung cancer and included in previous GWAS of lung cancer<br>Genomic DNA extracted from buffy coat and RBC	1,152	2,179	1,096	2,058	317K	317K
Arsenic Health Risk Assessment and Molecular Epidemiology study in Central Europe (ASHRAM)	Case-control study<br>Slovakia and Hungary	Cases recruited in 6 counties between 2002-2004<br>Controls matched to lung cancer and included in previous GWAS of lung cancer<br>Genomic DNA extracted from whole blood	89	425	84	399	610Q	317K
CeRePP<br>[additional to above]	Case series<br>France	Pathologically confirmed RCC involved in PROGENE-CeRePP cohort<br>French controls originally participated in a hospital based case-control study of UADT cancer<br>Genomic DNA extracted from blood	79	166	58	146	610Q	317K
The Leeds cohort	Case series<br>UK	RCC from the Leeds cohort recruited between 1998-2007<br>Genomic DNA extracted from blood	363	-	348	-	610Q	-
SEARCH Kidney cancer study (SEARCH)	Case series<br>UK	Population based case series including all patients with a kidney cancer aged 70- and diagnosed between 2002-2006<br>Genomic DNA extracted from blood	187	-	181	-	610Q	-
WTCCC	Controls<br>UK	Dataset generated by the Wellcome Trust Sanger Institute in collaboration with the 1958 Birth cohort<br>Genomic DNA extracted from blood	-	1,438	-	1,361	-	550K
Moscow	Case-control study<br>Moscow	Incident cases were recruited between 2007-2009. Hospital based controls were match and recruited during same period<br>Genomic DNA extracted from blood	305	323	263	305	610Q	610Q
**IARC -1 Scan Total**			**2,639**	**5,398**	**2,438**	**5,071**		
<u>**NCI-1 Scan:**</u>								
Alpha-Tocopherol, Beta-Carotene Cancer Prevention Study (ATBC)	See above	See above	177	1,288*	163	1,288*	660W	610K
American Cancer Society Cancer Prevention Study II Nutrition Cohort (CPS-II)	See above	See above	226	730*	202	730*	660W	610K
Prostate, Lung, Colorectal and Ovarian Cancer Screening Trial (PLCO)	See above	See above	330	845*	284	845*	660W	550/610K
National Cancer Institute U.S. Kidney Cancer Study (USKC)	Case-control;<br>USA (Chicago, Detroit)	Cases: 1,227 men and women aged 20-79 at diagnoses, identified from Chicago hospital records and Metropolitan Detroit Cancer Surveillance System between 2003 and 2007.<br>Controls: 1,235 men and women recruited from state Department of Motor Vehicle records (ages 30-64) and Medicare beneficiary records (ages 65+), frequency matched to cases on age, race, sex, region.<br>Genomic DNA extracted from blood and buccal cell samples.	720	595	662	561	610K	610K
**NCI-1 Scan Total:**			**1,453**	**3,458**	**1,311**	**3,424**		

**MD Anderson Scan**:	Case-control; USA (Texas)	Cases: 894 Caucasian men and women treated for renal cancer at the University of Texas MD Anderson Cancer Center. Controls:556 Caucasian men and women identified through random-digit dialing. Genomic DNA extracted from blood samples.	**894**	**556**	**893**	**556**	660W	660W
**UK Scan:**	Case series; UK	Cases: adult renal cancer patients identified from UK clinical oncology centers and the Institute of Cancer Research and Royal Marsden NHS Hospitals Trust. Controls: previously genotyped samples from the Wellcome Trust Case Control Consortium 2 1958 birth cohort and UK Blood Service Control Group (WTCC controls included in the IARC-1 scan were excluded). Genomic DNA extracted from blood samples.	**1,045**	**4,024**[*]	**944**	**4,024**[*]	Omni Express	Hap 1.2M Duo

[*]Previously scanned controls

Supplementary Table 2. Meta-analysis results for previously reported RCC GWAS loci

		Previously Reported GWAS				Current Meta-Analysis				
**SNP**	**Locus**	**Study**	**OR**	**(95% CI)**	***P***	**OR**	**(95% CI)**	***P***	$P_{Heterogeneity}$	**MAF**
rs3845536	1q24.1	Henrion et al.[1]	1.21	(1.13-1.30)	2.3×10^{-8}	1.05	(1.01-1.09)	0.0062	3.0×10^{-5}	0.36
rs7579899	2p21	Purdue et al.[2]	1.15	(1.10-1.21)	2.3×10^{-9}	1.15	(1.11-1.20)	5.3×10^{-15}	0.31	0.49
rs11894252	2p21	Purdue et al.[2]	1.14	(1.09-1.20)	1.8×10^{-8}	1.16	(1.12-1.20)	2.7×10^{-15}	0.28	0.49
rs12105918	2q22.3	Henrion et al.[3]	1.29	(1.18-1.41)	1.8×10^{-8}	1.25	(1.16-1.34)	3.9×10^{-9}	0.14	0.06
rs35252396*	8q24	Gudmundsson et al.[4]	1.27	(1.18-1.37)	5.4×10^{-11}	1.13	(1.10-1.18)	2.8×10^{-12}	0.17	0.44
rs7105934	11q13	Purdue et al.[2]	0.69	(0.62-0.76)	7.8×10^{-14}	0.70	(0.65-0.75)	4.9×10^{-22}	0.20	0.08
rs718314	12p11.23	Wu et al.[5]	1.19	(1.13-1.26)	8.9×10^{-10}	1.18	(1.14-1.23)	2.2×10^{-16}	0.45	0.26
rs1049380	12p11.23	Wu et al.[5]	1.18	(1.12-1.25)	6.1×10^{-9}	1.15	(1.11-1.19)	2.0×10^{-12}	0.02	0.44
rs4765623	12q24	Purdue et al.[2]	1.15	(1.09-1.20)	2.6×10^{-8}	1.14	(1.10-1.18)	2.8×10^{-12}	0.22	0.34

ORs computed for minor allele vs. common allele.
*Current meta-analysis based on rs64070588, in complete LD with rs35252396.

Supplementary Table 3. Description of the studies included in replication stage.

Study Name (Abbreviation)	Design; Location	Study Population	Genotyped Samples Cases	Controls
IARC Replication	Case-control and cohort studies; Europe and Australia	The sample set was composed of additional cases and controls selected from the following studies described in Supp table 1: IARC K2, CE, Umea, and ConFIRM/MCCS	1,674	4,222
Mayo Clinic (Mayo)	Case-control; USA	Cases: Caucasian men and women treated for RCC at Mayo. Controls: patients admitted to General Medicine Clinics at Mayo	909	1,479
MD Anderson (MDA)	Case-control; Houston, USA	Cases: Caucasian men and women treated for renal cancer at MDA. Controls: Caucasian men and women identified through random-digit dialing. (additional samples to these described in Supp table 1).	599	600

Supplementary Table 4. Technical validation of top SNPs; concordance rates between genome-wide-scanned or imputed SNPs and Taqman genotyping.

	IARC-1/2		NCI-2	
**SNP**	**% concordance**	**Pearson correlation coefficient (r^2)**	**% concordance**	**Pearson correlation coefficient (r^2)**
rs10936602 [a]	99.6	0.995	100.00	1.000
rs11637556 [b]	99.8	0.998	100.00	0.999
rs11813268 [b]	97.8	0.958	99.82	0.994
rs1266819 [b]	99.7	0.997	99.82	0.996
rs13376700 [b]	99.7	0.997	99.46	0.985
rs17050872 [a]	98.3	0.979	100.00	1.000
rs1800057 [b]	98.8	0.824	98.89	0.817
rs2109794 [b]	99.3	0.990	99.81	0.989
rs2203002 [a]	99.3	0.971	99.05	0.878
rs2241261 [a]	99.6	0.995		
rs234043 [a]	99.2	0.990	99.82	0.996
rs2889 [a]	99.1	0.990	99.64	0.991
rs4381241 [a]	99.7	0.997	99.82	0.991
rs4662750 [a]	97.0	0.967	97.90	0.909
rs4804368 [a]	99.8	0.997	99.82	0.993
rs4903064 [a]	98.9	0.985	99.05	0.948
rs59294613 [b]	99.9	0.999	100.00	1.000
rs6706003 [b]	98.1	0.980	98.23	0.944
rs67311347 [b]	98.9	0.985	98.68	0.863
rs6755594 [b]	98.1	0.979	99.26	0.967
rs714024 [b]	98.8	0.986	98.64	0.926
rs72730336 [b]	99.8	0.997	99.82	0.991
rs72851889 [b]	97.0	0.781	96.29	0.591
rs72855540 [b]	99.1	0.933	98.85	0.825
rs73149977 [a]	99.4	0.992	99.82	0.992
rs74911261 [a]	99.3	0.905	99.63	0.899
rs76912165 [b]	96.6	0.851	96.61	0.491
rs7697932 [b]	99.1	0.990	98.57	0.906
rs77736197 [a]	99.7	0.985	99.25	0.895
rs77774900 [a]	99.0	0.970	99.42	0.906
rs7913447 [b]	98.3	0.980	98.85	0.941
rs8007348 [b]	99.8	0.997	100.00	1.000

[a]Imputed or genotyped across scans; [b]imputed across all scans.

Supplementary Table 5. Associations with 13-SNP RCC polygenic risk score (PRS): overall, and by histology, age at onset and stage

Analysis		OR	95% CI	P
All RCC				
Across PRS deciles				
	1	1.00		
	2	1.34	(1.16-1.54)	3.60E-05
	3	1.41	(1.23-1.62)	8.30E-07
	4	1.56	(1.36-1.79)	1.00E-10
	5	1.71	(1.50-1.96)	1.98E-15
	6	1.93	(1.69-2.20)	8.40E-23
	7	1.93	(1.69-2.20)	7.20E-23
	8	2.11	(1.85-2.40)	1.20E-29
	9	2.52	(2.22-2.86)	8.40E-46
	10	3.24	(2.86-3.67)	1.20E-76
Continuous (per PRS unit increase)		2.85	(2.62-3.11)	2.30E-125
By histology (per PRS unit increase)				
Clear cell RCC vs. controls		3.24	(2.91-3.62)	3.40E-100
Papillary RCC vs. controls		1.83	(1.44-2.32)	5.30E-07
Chromophobe RCC vs. controls		2.34	(1.58-3.46)	2.40E-05
By RCC age at onset (per PRS unit increase)				
<60 vs. 60+		1.01	(0.88-1.15)	9.10E-01
By stage (per PRS unit increase)				
2 vs. 1		0.92	(0.63-1.36)	6.90E-01
3 vs. 1		1.24	(0.91-1.70)	1.80E-01
4 vs. 1		1.36	(0.97-1.93)	8.00E-02

Supplementary Note

Additional acknowledgement:

AHS

This research was supported by the Intramural Research Program of the NIH, National Cancer Institute, Division of Cancer Epidemiology and Genetics (Z01CP010119).

ATBC

The ATBC Study was supported by funding provided by the Intramural Research Program of the NCI, NIH, and through U.S. Public Health Service contracts (N01-CN-45165, N01-RC-45035 and N01-RC-37004) from the NCI.

BioVU

The dataset used in the analyses described were obtained from the Vanderbilt University Medical Center resource BioVU, which is supported by institutional funding, the 1S10RR025141-01 instrumentation award, and by the Vanderbilt CTSA grant UL1 TR000445 from NCATS/NIH. Sample processing and phenotyping algorithm development was supported by institutional funding for TLE.

Center 'Bioengineering' of the Russian Academy of Sciences/Kurchatov Scientific Center

The work conducted for this study was supported by the grant Russian Scientific Fund 14-14-01202.

Centre National de Genotypage, France

We thank Jean Guillaume Garnier and Delphine Bacq-Daian for their work on the IARC-2 scan.

ConFIRM/MCCS

The ConFIRM study, also known as CARES, was supported by the Victorian Cancer Agency (PTCB08_05), the Australian National Health and Medical Research Council (Project Grant 1011626). We acknowledge the contribution of Professor Graham Giles in supporting this work and of Ms Olive Schmid and Ms Jennifer Walsh for the project management.

The Melbourne Collaborative Cohort Study (MCCS) recruitment was funded by VicHealth and Cancer Council Victoria. The MCCS was further supported by Australian NHMRC grants 209057, 251553 and 504711 and by infrastructure provided by Cancer Council Victoria.

CPS-II

The Cancer Prevention Study II Nutrition Cohort is supported by the American Cancer Society. The authors thank all of the men and women in the Cancer Prevention Study II Nutrition Cohort for their many years of dedicated participation in the study.

Health Professionals Follow-up Study (HPFS) and Nurses' Health Study (NHS)

The HPFS is supported by National Institutes of Health, National Cancer Institute grant UM1 CA167552. The NHS is supported by National Institutes of Health, National Cancer Institute grants UM1 CA186107 and P01 CA87969. We would like to thank the following state cancer registries for their help: AL, AZ, AR, CA, CO, CT, DE, FL, GA, ID, IL, IN, IA, KY, LA, ME, MD, MA, MI, NE, NH, NJ, NY, NC, ND, OH, OK, OR, PA, RI, SC, TN, TX, VA, WA, WY. The authors assume full responsibility for analyses and interpretation of these data.

K2 study

This study was supported by the EU FP7 under grant agreement number 241669 (the CAGEKID project). In Czech Republic, this work was also supported by MH CZ—DRO (MMCI, 00209805) and by the project MEYS – NPS I – LO1413, Czech Republic.

Leeds Cohort

The infrastructure support from Cancer Research UK as part of the Leeds Centre and Experimental Cancer Medicine Centre funding is gratefully acknowledged together with the Leeds Multidisciplinary Research Tissue Bank, all patients who consented to take part in the research studies and the staff of the Urology and Oncology Departments in Leeds Teaching Hospitals Trust.

Mayo Clinic

This study was partially supported by National Institutes of Health: R21CA176422 (JEEP) and R01CA134466 (ASP). The authors acknowledge the Mayo Clinic Comprehensive Cancer Center Biospecimens Accessioning and Processing Shared Resource and the Pathology Research Core Shared Resource.

MD Anderson

This work was supported in part by the NIH (grant R01 CA170298) and the Center for Translational and Public Health Genomics, Duncan Family Institute for Cancer Prevention and Risk Assessment, The University of Texas MD Anderson Cancer Center.

NCI/IARC RCC Study in Central Europe (CE)

This project was supported by the Intramural Research Program of the NIH and the National Cancer Institute.

Physicians' Health Study (PHS)

This study was supported by grants CA 097193, CA 34944, CA 40360, HL 26490, and HL 34595 from the National Institutes of Health, Bethesda, MD.

PLCO

This research was supported by the Intramural Research Program of the National Cancer Institute and by contracts from the Division of Cancer Prevention, National Cancer Institute, NIH, DHHS.

SEARCH

This study is funded by Cancer Research UK (C490/A16651).

UK GWAS

We acknowledge support from the Medical Research Council (MRC), Cancer Research UK, an educational grant from Bayer and NHS funding for the Royal Marsden Biomedical Research Centre and Cambridge University Health Partners. JL is supported by the NIHR RM/CR Biomedical Research Centre for Cancer.

US Kidney Cancer Study

The NCI United Stated Kidney Cancer Study was supported by the Intramural Research Program of the National Institutes of Health and the National Cancer Institute under the following

contracts: N02-CP-10128 (Westat, Inc.), N02-CP-11004 (Wayne State University), and N02-CP-11161 (University of Illinois at Chicago).

Van Andel Research Institute (VARI)

The authors would like to thank Dr. Kyle Furge for his role in this project as well as Drs. Anthony Avallone, John Ludlow and Philip Wise for contributing samples for this project.

Women's Health Initiative (WHI)

The WHI program is funded by the National Heart, Lung, and Blood Institute, National Institutes of Health, U.S. Department of Health and Human Services through contracts

HHSN268201100046C, HHSN268201100001C, HHSN268201100002C, HHSN268201100003C, HHSN268201100004C, and HHSN271201100004C. For a list of all the investigators who have contributed to WHI science, please visit: https://www.whi.org/researchers/Documents%20%20Write%20a%20Paper/WHI%20Investigato r %20Long%20List.pdf".

Women's Health Study (WHS)

The study is supported by grants CA-047988, HL-043851, HL-080467, HL-099355, and UM1 CA182913 from the National Institutes of Health, Bethesda, MD.

Supplementary References

1. Henrion, M.Y. *et al.* Common variation at 1q24.1 (ALDH9A1) is a potential risk factor for renal cancer. *PLoS One* **10**, e0122589 (2015).
2. Purdue, M.P. *et al.* Genome-wide association study of renal cell carcinoma identifies two susceptibility loci on 2p21 and 11q13.3. *Nat Genet* **43**, 60-5 (2011).
3. Henrion, M. *et al.* Common variation at 2q22.3 (ZEB2) influences the risk of renal cancer. *Hum Mol Genet* **22**, 825-31 (2013).
4. Gudmundsson, J. *et al.* A common variant at 8q24.21 is associated with renal cell cancer. *Nat Commun* **4**, 2776 (2013).
5. Wu, X. *et al.* A genome-wide association study identifies a novel susceptibility locus for renal cell carcinoma on 12p11.23. *Hum Mol Genet* **21**, 456-62 (2012).
